# Effects of neonatal nutrition interventions on neonatal mortality and child health and development outcomes: A systematic review

**DOI:** 10.1002/cl2.1141

**Published:** 2021-03-05

**Authors:** Aamer Imdad, Faseeha Rehman, Evans Davis, Deepika Ranjit, Gamael S. S. Surin, Suzanna L. Attia, Sarah Lawler, Abigail A. Smith, Zulfiqar A. Bhutta

**Affiliations:** ^1^ Department of Pediatrics, Division of Pediatric Gastroenterology, Hepatology and Nutrition SUNY Upstate Medical University Syracuse New York USA; ^2^ Department of Medicine Raritan Bay Medical Center Perth Amboy New York USA; ^3^ Roswell Park Comprehensive Cancer Center, Department of Cancer Prevention and Control University of Buffalo Buffalo New York USA; ^4^ College of Medicine SUNY Upstate Medical University Syracuse New York USA; ^5^ Department of Pediatrics, Division of Pediatric Gastroenterology, Hepatology and Nutrition University of Kentucky Lexington Kentucky USA; ^6^ Health Science Library SUNY Upstate Medical University Syracuse New York USA; ^7^ Health Science Libraray SUNY Upstate Medical University Syracuse New York USA; ^8^ Centre for Global Child Health The Hospital for Sick Children Toronto Ontario Canada

## Abstract

**Background:**

The last two decades have seen a significant decrease in mortality for children <5 years of age in low and middle‐income countries (LMICs); however, neonatal (age, 0–28 days) mortality has not decreased at the same rate. We assessed three neonatal nutritional interventions that have the potential of reducing morbidity and mortality during infancy in LMICs.

**Objectives:**

To determine the efficacy and effectiveness of synthetic vitamin A, dextrose oral gel, and probiotic supplementation during the neonatal period.

**Search Methods:**

We conducted electronic searches for relevant studies on the following databases: PubMed, CINAHL, LILACS, SCOPUS, and CENTRAL, Cochrane Central Register for Controlled Trials, up to November 27, 2019.

**Selection Criteria:**

We aimed to include randomized and quasi‐experimental studies. The target population was neonates in LMICs. The interventions included synthetic vitamin A supplementation, oral dextrose gel supplementation, and probiotic supplementation during the neonatal period. We included studies from the community and hospital settings irrespective of the gestational age or birth weight of the neonate.

**Data Collection and Analysis:**

Two authors screened the titles and extracted the data from selected studies. The risk of bias (ROB) in the included studies was assessed according to the Cochrane Handbook of Systematic Reviews. The primary outcome was all‐cause mortality. The secondary outcomes were neonatal sepsis, necrotizing enterocolitis (NEC), prevention and treatment of neonatal hypoglycaemia, adverse events, and neurodevelopmental outcomes. Data were meta‐analyzed by random effect models to obtain relative risk (RR) and 95% confidence interval (CI) for dichotomous outcomes and mean difference with 95% CI for continuous outcomes. The overall rating of evidence was determined by the Grading of Recommendations Assessment, Development, and Evaluation (GRADE) approach.

**Main Results:**

Sixteen randomized studies (total participants 169,366) assessed the effect of vitamin A supplementation during the neonatal period. All studies were conducted in low‐ and middle‐income (LMIC) countries. Thirteen studies were conducted in the community setting and three studies were conducted in the hospital setting, specifically in neonatal intensive care units. Studies were conducted in 10 different countries including India (four studies), Guinea‐Bissau (three studies), Bangladesh (two studies), and one study each in China, Ghana, Indonesia, Nepal, Pakistan, Tanzania, and Zimbabwe. The overall ROB was low in most of the included studies for neonatal vitamin A supplementation. The pooled results from the community based randomized studies showed that there was no significant difference in all‐cause mortality in the vitamin A (intervention) group compared to controls at 1 month (RR, 0.99; 95% CI, 0.90–1.08; six studies with 126,548 participants, statistical heterogeneity *I* ^2^ 0%, funnel plot symmetrical, grade rating high), 6 months (RR, 0.98; 95% CI, 0.89–1.07; 12 studies with 154,940 participants, statistical heterogeneity *I* ^2^ 43%, funnel plot symmetrical, GRADE quality high) and 12 months of age (RR, 1.04; 95% CI, 0.94–1.14; eight studies with 118,376 participants, statistical heterogeneity *I* ^2^ 46%, funnel plot symmetrical, GRADE quality high). Neonatal vitamin A supplementation increased the incidence of bulging fontanelle by 53% compared to control (RR, 1.53; 95% CI, 1.12–2.09; six studies with 100,256 participants, statistical heterogeneity *I* ^2^ 65%, funnel plot symmetrical, GRADE quality high). We did not identify any experimental study that addressed the use of dextrose gel for the prevention and/or treatment of neonatal hypoglycaemia in LMIC. Thirty‐three studies assessed the effect of probiotic supplementation during the neonatal period (total participants 11,595; probiotics: 5854 and controls: 5741). All of the included studies were conducted in LMIC and were randomized. Most of the studies were done in the hospital setting and included participants who were preterm (born < 37 weeks gestation) and/or low birth weight (<2500 g birth weight). Studies were conducted in 13 different countries with 10 studies conducted in India, six studies in Turkey, three studies each in China and Iran, two each in Mexico and South Africa, and one each in Bangladesh, Brazil, Colombia, Indonesia, Nepal, Pakistan, and Thailand. Three studies were at high ROB due to lack of appropriate randomization sequence or allocation concealment. Combined data from 25 studies showed that probiotic supplementation reduced all‐cause mortality by 20% compared to controls (RR, 0.80; 95% CI, 0.66–0.96; total number of participants 10,998, number needed to treat 100, statistical heterogeneity *I* ^2^ 0%, funnel plot symmetrical, GRADE quality high). Twenty‐nine studies reported the effect of probiotics on the incidence of NEC, and the combined results showed a relative reduction of 54% in the intervention group compared to controls (RR, 0.46; 95% CI, 0.35–0.59; total number of participants 5574, number needed to treat 17, statistical heterogeneity *I* ^2^ 24%, funnel plot symmetrical, GRADE quality high). Twenty‐one studies assessed the effect of probiotic supplementation during the neonatal period on neonatal sepsis, and the combined results showed a relative reduction of 22% in the intervention group compared to controls (RR, 0.78; 95% CI, 0.70–0.86; total number of participants 9105, number needed to treat 14, statistical heterogeneity *I* ^2^ 23%, funnel plot symmetrical, GRADE quality high).

**Authors' Conclusions:**

Vitamin A supplementation during the neonatal period does not reduce all‐cause neonatal or infant mortality in LMICs in the community setting. However, neonatal vitamin A supplementation increases the risk of Bulging Fontanelle. No experimental or quasi‐experimental studies were available from LMICs to assess the effect of dextrose gel supplementation for the prevention or treatment of neonatal hypoglycaemia. Probiotic supplementation during the neonatal period seems to reduce all‐cause mortality, NEC, and sepsis in babies born with low birth weight and/or preterm in the hospital setting. There was clinical heterogeneity in the use of probiotics, and we could not recommend any single strain of probiotics for wider use based on these results. There was a lack of studies on probiotic supplementation in the community setting. More research is needed to assess the effect of probiotics administered to neonates in‐home/community setting in LMICs.

## 1 PLAIN LANGUAGE SUMMARY

### 1.1 Neonatal probiotic supplementation can improve infant illness and reduce death, but vitamin A does not, and may have adverse effects

Nutritional support during the 1st month of life is vital for the short‐ and long‐term survival of the newborn. Neonatal nutrition interventions have the potential to decrease death and illness in young infants in LMICs.

#### 1.1.1 What is this review about?

This review assesses the efficacy of synthetic vitamin A, dextrose, and probiotic supplementation during the neonatal period. These interventions were assessed separately and not in combination with each other.

**What is the aim of this review?**

This Campbell systematic review assesses the efficacy of three neonatal nutritional interventions that have the potential of reducing morbidity and mortality during infancy in LMICs: synthetic vitamin A, dextrose, and probiotic supplementation.

#### 1.1.2 What studies are included?

Sixteen studies that assessed the effect of vitamin A supplementation during the neonatal period were included. Thirteen of these studies were conducted in the community setting and three studies were conducted in the hospital setting. All the included studies on neonatal vitamin A supplementation were conducted in LMICs. Most of the studies had a low ROB.

No experimental studies were found that evaluated the use of dextrose for the prevention or treatment of low blood sugar during the neonatal period.

Thirty‐three studies assessed the use of probiotics during the 1st month of life. All included studies on probiotic supplementation were randomized and conducted in LMICs. Most of the included studies had a low ROB. The probiotics studies mainly included babies born early and/or with low birth weight, and these studies were mostly conducted in hospital settings.

#### 1.1.3 Key results

Combined results from thirteen vitamin A studies conducted in the community settings showed that there was no significant effect of vitamin A supplementation for reduction of death in young infants at 1, 6, or 12 months of age. Neonatal vitamin A supplementation increases the risk of bulging fontanelle by 53%. The pooled data from probiotics studies showed that this intervention reduced the risk of death by 20% compared to controls. Further analysis showed that compared to controls, probiotic supplementation reduced the risk of a severe form of gastrointestinal illness in neonates called NEC by 54%. Probiotic supplementation also reduced the risk of blood infection called sepsis by 22% compared to controls. The quality grade ratings for these outcomes were “high.”

#### 1.1.4 What are the main findings of the review?

Combined results from thirteen vitamin A studies conducted in the community settings showed that there was no significant effect of vitamin A supplementation for reduction of death in young infants at 1, 6, or 12 months of age. However, neonatal vitamin A supplementation increased the incidence of bulging fontanelle by 53%.

The pooled data from probiotics studies showed that this intervention reduced the risk of death by 20% compared to controls. Further analysis showed that compared to controls, probiotic supplementation reduced the risk of a severe form of gastrointestinal illness in neonates called NEC by 54%. Probiotic supplementation also reduced the risk of blood infection called sepsis by 22% compared to controls. The quality grade ratings for these outcomes were “high.”

#### 1.1.5 What do the findings of this review mean?

Vitamin A supplementation during the 1st month of life does not reduce the risk of death during the 1st year of life in LMICs. However, neonatal vitamin A supplementation increases the risk of bulging fontanelle, which may cause damage to the brain.

We did not find any experimental studies from LMICs that assessed the use of dextrose gel supplementation during the 1st month of life for the prevention or treatment of low blood sugar.

Probiotic supplementation during the 1st month of life to babies born preterm and/or low birthweight can reduce the risk of death, blood infection and bowel sickness (NEC).

There was clinical heterogeneity in the use of probiotics and we could not recommend any single strain or combination of probiotics for wider use based of these results.

There is a lack of studies on probiotic supplementation in the 1st month of life in community settings. More research is needed to assess the effect of probiotics administered to neonates in home/community settings in LMICs.

#### 1.1.6 How up‐to‐date is this review?

The review authors searched for studies published up to November 2019.

## 2 BACKGROUND

The decline in rates of neonatal (age, 0–28 days) mortality has been slower than the decline in child mortality between 1990 and 2016 (Alkema et al., 2014; Bhutta et al., 2015). Neonatal mortality accounted for 46% of child mortality in 2016 compared to 40% of all under‐five mortality rates in 1990 (WHO, 2017a). Globally, the percentage of neonatal mortality is the highest in South Asia and Sub‐Saharan Africa (Alkema et al., 2014). Optimal nutritional support during the neonatal period is vital to the short and long term survival of the newborn (Bhutta et al., 2013; WHO, 2017b). Poor nutritional status of neonates is a major cause of illness and can lead to poor growth, increased risk of infection, bleeding, and neonatal death (Bhutta et al., 2013; WHO, 2017b). The risk of morbidity and mortality during the neonatal period is higher in LMICs where many births happen at home and the prevalence of maternal malnutrition and incidence of low birth weight (birth weight <2500 g) and preterm birth (gestational age <37 weeks) is high (Bhutta et al., 2013; Lee et al., 2017; WHO, 2017b). This review focused on three nutritional interventions during neonatal periods that have the potential to reduce illness and death during infancy in LMIC.

### 2.1 Description of the condition

The approach to nutritional management of newborn depends on maternal nutritional status, comorbidities during pregnancy (such as gestational diabetes), pregnancy duration (term vs. preterm birth), events at birth (such as birth asphyxia), birth weight (low birth weight vs. normal birth weight) and available resources for postpartum care of the mother and the baby (such as skilled birth attendant, home vs. facility birth, availability of neonatal intensive care) (Bhutta et al., 2013; WHO, 2015, 2017a, 2017b). The most important nutritional intervention after birth is breastfeeding, which is covered in a separate Campbell review of this series. There are a number of other nutritional interventions that have been proposed in addition to breastfeeding. It is beyond the scope of this review to comprehensively evaluate all the possible nutritional interventions during the neonatal period. We limited our review to the following three interventions: neonatal synthetic vitamin A supplementation, oral dextrose gel supplementation, and probiotic supplementation during the neonatal period in LMIC. Below in this section and in the rest of the introduction, we describe the rationale and importance of reviewing these interventions.

#### 2.1.1 Neonatal vitamin A deficiency (VAD)

Globally, about 190 million children and 19.1 million pregnant women are vitamin A deficient based on serum retinol levels (i.e., serum retinol <0.70 μmol/L) (WHO, 2009a). VAD is most prevalent in South Asia and Africa (Stevens et al., 2015). VAD is associated with increased risk of blindness, infections, and mortality (Imdad et al., 2017). Most of the newborns are vitamin A deficient and rely on supplementation from maternal breast milk (Haider et al., 2017). High prevalence of maternal VAD in LMICs increases the risk of neonatal VAD. There has been interest in vitamin A supplementation during neonatal period to assess if it reduces risk of illness and death (Haider et al., 2017; WHO, 2009b), as it has been shown to reduce morbidity and mortality in children 6–59 months of age (Imdad et al., 2017).

#### 2.1.2 Hypoglycemia during the neonatal period

Hypoglycemia (low blood sugar) is common during the immediate neonatal period. The definition of neonatal hypoglycaemia varies. The American Academy of Pediatrics defines neonatal hypoglycaemia as blood glucose below 47 mg/dl (2.61 mmol/L); however, other societies such as the Pediatric Endocrine Society define neonatal hypoglycaemia as blood glucose <50 mg/dl (2.77 mmol/L; Thompson‐Branch & Havranek, 2017; Thornton et al., 2015). Recurrent, severe, and/or persistent hypoglycaemia can lead to complications such as death; there is limited evidence to show that blood sugars below a certain level leads to long‐term brain damage (Kaiser et al., 2015; McKinlay et al., 2017; Thornton et al., 2015). About 10–15% of otherwise healthy newborns have low blood sugar, and the rate is much higher among infants with additional risk factors such as large for gestational age, small for gestational age, low birth weight, preterm birth, infant of diabetic mother, and newborns with perinatal asphyxia (Thompson‐Branch & Havranek, 2017). Additional risk factors for neonatal hypoglycaemia include neonatal sepsis, prolonged labor, and maternal medication use such as use of β‐agonists and β‐blockers (Thompson‐Branch & Havranek, 2017). The recommended initial intervention to treat neonatal hypoglycaemia is to offer feeding in the form of breastfeeding followed by formula feeding if breastfeeding is unsuccessful. Persistent hypoglycaemia may require IV dextrose supplementation and admission to a neonatal intensive care unit (Thompson‐Branch & Havranek, 2017; Thornton et al., 2015). In LMIC, where a significant proportion of births happen at home and incidence of low birth weight and preterm birth is high, prevention and treatment of hypoglycaemia encounters additional challenges (Singhal et al., 1991, 1992; WHO, 2017b; Williams, 1997). The instruments to test blood sugar might not be available in low‐resource settings; In addition, formula, IV dextrose, and intensive care units might not be available to treat persistent and/or severe hypoglycaemia. Recent studies have tested simple interventions such as oral dextrose gel to treat neonatal hypoglycaemia and to prevent hypoglycaemia in high‐risk newborns (Hegarty et al., 2016; Weston et al., 2016).

#### 2.1.3 Neonatal sepsis and NEC

Neonatal sepsis and NEC are neonatal morbidities that can be fatal (Oza et al., 2015; WHO, 2017b). Neonatal sepsis is the presence of an infection leading to systemic illness. Bacterial sepsis is common in LMIC and is a significant risk factor of morbidity and mortality in these countries (WHO, 2017a). NEC is a condition that occurs in newborns and can lead to intestinal injury and death. The extent of injury may vary from mucosal injury to full thickness intestinal wall injury. NEC happens most commonly in preterm babies and especially in extremely preterm babies (<28 weeks gestational age; AlFaleh & Anabrees, 2014; Patel & Denning, 2015). Multiple factors lead to the development of NEC in preterm infants including altered bacterial gut flora affecting the protective intestinal barrier, decreased intestinal motility, and the increased susceptibility of preterm infants to inflammation and infections (Patel & Denning, 2015). Recent studies have shown that an imbalance between commensal bacteria and pathogenic bacteria (intestinal dysbiosis) makes babies vulnerable to pathogenic bacterial growth in the intestine which then causes inflammation that may contribute to neonatal sepsis and/or NEC (Arrieta et al., 2014; Deshmukh et al., 2014; Gewolb et al., 1999; Panigrahi et al., 2017). There is an increasing interest in correction of intestinal dysbiosis by probiotics to prevent NEC and neonatal sepsis. Data from early studies on probiotic use in neonates from LMIC is encouraging (AlFaleh & Anabrees, 2014; Rao et al., 2016).

### 2.2 Description of the intervention

#### 2.2.1 Neonatal vitamin A supplementation

Vitamin A is a term used for a subclass of the family of fat soluble compounds named retinoic acids. Vitamin A is found in nature in two forms, provitamin A carotenoids and preformed vitamin A, which is essential to human bodily function. Plant‐based foods are the main source of provitamin A carotenoids, of which β‐carotene is the most commonly known. Animal‐based foods are the main source of preformed vitamin A (Bates, 1995; Haider & Bhutta, 2011). Vitamin A from animal sources (retinol, retinal, retinoic acid, and retinyl esters) is the most active form, and synthetic vitamin A retinol has been used in most intervention trials in the past (Haider & Bhutta, 2011; Imdad et al., 2017). Plant‐based foods may not be an adequate source of vitamin A, as the gastrointestinal conversion ratio from carotenoid‐to‐retinol varies from 6:1 to 26:1 (US Institute of Medicine, Food and Nutrition Board). VAD may, therefore, exist in areas even when there is high consumption of plant‐based foods such as in South Asia and Africa (Imdad et al., 2017; Stevens et al., 2015).

#### 2.2.2 Oral dextrose gel supplementation during neonatal period

Dextrose gel is a thickened aqueous solution that contains the concentrated simple carbohydrate dextrose. It can be administered by direct application to oral, buccal, or sublingual mucosa and can increase blood sugars rapidly by absorption through the highly vascularized and thin mucus membranes of the oral mucosa (Hegarty et al., 2016). Detxrose gel is a low cost nonproprietary intervention, and the gel can be prepared in hospital pharmacies. The typical ingredients include water, glucose, a gelling agent, and preservatives (Hegarty et al., 2016). The decision to use dextrose gel in a neonate should be taken on individual basis and should be avoided in neonates with compromised neurological or respiratory status (Hegarty et al., 2016; Weston et al., 2016).

#### 2.2.3 Probiotic supplementation during neonatal period

Prebiotics are supplements that promote the growth of commensal bacteria (AlFaleh & Anabrees, 2014; Panigrahi et al., 2017). Probiotics contain live bacteria that enrich pools of commensal bacteria (AlFaleh & Anabrees, 2014; Millar et al., 2003; Panigrahi et al., 2017). Synbiotics are a combination of prebiotics and probiotics and might have synergistic effect (Johnson‐Henry et al., 2016, Nandhini et al., 2016; Panigrahi et al., 2017). These supplements are meant to optimise gut health and their hypothesized mechanisms of actions include enhanced gut barrier function, inhibition of gut colonization with pathogenic bacteria, improvement in colonization with healthy commensal bacteria that protect the infant from enteropathogenic infection through production of acetate, enhanced innate immunity, and increased maturation of the enteric nervous system (Rao et al., 2016). Recent data have shown that probiotic supplements can prevent the incidence of NEC in preterm babies (AlFaleh & Anabrees, 2014; Millar et al., 2003; Patel & Denning, 2015; van den Akker et al., 2018). There is also promising data on use of probiotocs/synbiotics for prevention of neonatal sepsis (Rao et al., 2016; Panigrahi et al., 2017). The most commonly used strains of probiotics include *Lactobacillus* and *Bifidobacterium* (Rao et al., 2016).

### 2.3 How the intervention might work

#### 2.3.1 Neonatal vitamin A supplementation

Vitamin A has an effect on cell differentiation and helps maintain normal functioning of epithelial cells (Bates, 1995; Bhutta et al., 2013; Haider & Bhutta, 2011). It is considered anti‐infective because it helps to maintain the protective epithelial barrier of the skin and mucosa, which protects the body from infections. Vitamin A helps in the regeneration of the epithelium and therefore maintains the integrity of the body's first line of defence. These mechanisms may help prevent infections in newborns (McCullough et al., 1999; Wolbach, 1933). Synthetic vitamin A supplementation has been shown to reduce morbidity and mortality in children 6–59 months of age (Imdad et al., 2017). The potential side effects of synthetic vitamin A supplementation include vomiting and bulging fontanelle (Imdad et al., 2016, 2017; Haider & Bhutta, 2011; Haider et al., 2017). Excess vitamin A supplementation can cause toxicity that presents in the form of a bulging fontanelle in children under 1 year, headaches, vomiting, diarrhea, loss of appetite, and irritability (Haider et al., 2017; Imdad et al., 2017).

#### 2.3.2 Oral dextrose gel supplementation during neonatal period

The absorption of dextrose gel through the oral mucosa leads to entry of glucose into lingual veins and into the internal jugular vein. This pathway provides almost immediate delivery of glucose to the systemic circulation and bypasses first pass liver metabolism through the portal circulation. If proven effective in preventing and treating hypoglycaemia, dextrose gel can avoid the need of intravenous glucose and reduce separation of baby from mother (Hegarty et al., 2016; Weston et al., 2016). The intervention is simple enough that it does not require special skills (such as IV placement) and can be administered by community, lay health workers, or the caregiver herself. Potential adverse effects include vomiting, choking, gagging, respiratory distress, and delay of treatment for severe hypoglycaemia (Hegarty et al., 2016; Weston et al., 2016).

### 2.4 Probiotics supplementation during neonatal period

Newborn, and especially preterm, babies have immature intestines free of normal commensal bacteria that would normally protect them from developing NEC and sepsis by inhibiting the growth of pathogenic bacteria in the intestines (AlFaleh & Anabrees, 2014; Patel & Denning, 2015; Rao et al., 2016). Probiotics are used to proactively colonize the intestines with beneficial bacteria such as *Lactobacillus* species (Millar et al., 2003; Patel & Denning, 2015). Probiotics therefore reduce the growth of pathogenic bacteria which would otherwise increase the risk of NEC and sepsis. Also probiotics promote gut immunity by increasing IgA levels and contributing to improved mucosal barrier function (Patel & Denning, 2015). These protective mechanisms reduce intestinal permeability by producing a protective mucosal barrier against bacteria and increase the production of anti‐inflammatory cytokines (Deshpande et al., 2017; Millar et al., 2003). Probiotics are especially protective in preterm babies with immature intestinal microbiomes and neonates on antibiotics; antibiotics may reduce bacterial diversity in the intestine and thus also dispose to colonization by pathogenic bacteria causing NEC. Prebiotics and probiotics can be given together in the form of a synbiotic to improve the gut flora and can potentially reduce all‐cause neonatal mortality (Johnson‐Henry et al., 2016; Panigrahi et al., 2017). Probiotics are considered safe; however, there are concerns regarding probiotic supplementation in extremely premature or immunocompromised neonates. A few cases of neonatal sepsis have been reported that were thought to be caused by probiotics (Dani et al., 2016).

### 2.5 Why it is important to do this review

#### 2.5.1 Neonatal vitamin A supplementation

Randomized trials on neonatal vitamin A supplementation have produced conflicting results with some studies (mostly from South Asia) showing a mortality benefit while no major benefit in other studies (mostly from Africa) (Haider et al., 2017) and some studies showing even an increased risk of infant mortality in certain populations (Smith et al., 2016).The exact reason for this difference in results is not clear. Previous reviews (Haider et al., 2017; Gogia & Sachdev, 2009) and a WHO technical consultation (WHO, 2009b) have hypothesized on what factors may explain these varied results. Our group has previously published a Cochrane review on the evidence on neonatal vitamin A supplementation (Haider & Bhutta, 2011), and we wanted to update the previous review. The previous review included studies conducted in the community setting. In this review, we considered studies conducted in both the community and the hospital setting in LMIC. We also included neurodevelopment outcomes for this review that were not covered in the previous Cochrane review.

#### 2.5.2 Oral dextrose gel supplementation during neonatal period

Oral dextrose gel has been studied in the prevention and treatment of neonatal hypoglycaemia in high‐income countries (Hegarty et al., 2017; Weston et al., 2016); however, it was not clear if similar studies were available from LMICs. Our objective was to consider both randomized and nonrandomized observational studies with a control arm. We hypothesized that the use of dextrose may be more beneficial in LMIC than in high‐income countries, as the incidence of neonatal hypoglycaemia might be higher in these countries due to an increased rate of preterm and low birth weight birth.

#### 2.5.3 Probiotics supplementation during neonatal period

The effect of probiotic supplementation for the prevention of NEC and neonatal sepsis has been assessed in previous reviews (AlFaleh & Anabrees, 2014; Rao et al., 2016; van den Akker et al., 2018). Most of these reviews included studies from both high‐ and LMICs. Deshpande et al. (2017) reviewed studies from LMIC where neonates were supplemented with probiotics. More studies (Amini et al., 2017; Chowdhury et al., 2016; Guney‐Varal et al., 2017; Hernández‐Enríquez et al., 2016; Hussain et al., 2016) have been published since the publication of Deshpande et al.'s (2017) review. Overall, our objective was to assess the current evidence for the effect of probiotic supplementation during the neonatal period in the hospital and community setting in LMIC.

## 3 OBJECTIVES

### 3.1 Primary objectives

To determine the efficacy of the following interventions on neonatal morbidity and mortality:

1. Synthetic vitamin A supplementation,
2. Oral dextrose gel supplementation, and
3. Oral probiotic supplementation.

A detailed description of background and methods for this review was published in the form of a protocol as Imdad et al. (2019).

## 4 METHODS

### 4.1 Criteria for considering studies for this review

#### 4.1.1 Types of studies

We included the following study designs:

- Randomized controlled trials (RCTs), where participants were randomly assigned either individually or in clusters to intervention and comparison groups. Cross‐over designs were also eligible for inclusion.
- Quasi‐experimental designs, which include:

a. Natural experiments: studies where nonrandom assignment was determined by factors that were out of the control of the investigator. One common type includes allocation based on exogenous geographical variation.
b. Controlled before‐after studies (CBA), in which measures were taken of an experimental group and a comparable control group both before and after the intervention. We also require that appropriate methods were used to control for confounding, such as statistical matching (e.g., propensity score matching or covariate matching) or regression adjustment (e.g., difference‐in‐differences, instrumental variables).
c. Regression discontinuity designs; here, allocation to intervention/control was based upon a cut‐off score.
d. Interrupted time series studies, in which outcomes were measured in the intervention group at at least three time points before the intervention and after the intervention.

#### 4.1.2 Types of participants

Participants for this review included neonates (aged 0–28 days) from LMICs. We included neonates regardless of their health status. This includes low birth weight and preterm babies. However, studies that focused on neonates with congenital anomalies were excluded. We considered studies that included older age population groups in addition to neonates only if we could disaggregate relevant data for the neonatal population. For example, a study might include infants up to 6 months of age. We included such a study if the disaggregated data were available for neonates (0–28 days). Even though we planned to assess later childhood outcomes, we did not plan to include studies that recruited participants after the neonatal period.

#### 4.1.3 Types of interventions

The following interventions were included in the review:

1. Neonatal vitamin A supplementation compared to no supplementation or placebo: we considered only oral synthetic vitamin A supplementation. There was no restriction on the dosage and frequency of the medicine. The comparison group could include a placebo or standard of care.
2. Oral dextrose gel supplementation during the neonatal period compared to no supplementation: we placed no limits on the dose or frequency of the dextrose supplementation. We only considered dextrose gel as the intervention and excluded dextrose given in other forms such as intravenous, nasogastric tube, or mixed with infant formula. The reason to exclude forms other than dextrose gel was that administration of dextrose in those forms may require special circumstances (like trained staff to place an IV) or special delivery vehicles, such as formula, that may not be available in LMIC. The comparison group included placebo or standard of care.
3. Neonatal oral probiotics/synbiotics compared to no probiotic supplementation: probiotics are live microbial organisms that are given to promote the growth of commensal gut bacteria and prevent the growth of pathogenic bacteria. Prebiotics are dietary supplements that promote the growth of commensal bacteria. Synbiotics are a combination of prebiotics and probiotics (Millar et al., 2003; Patel & Denning, 2015). We placed no limits on the dose or frequency of probiotics. We included studies that used probiotics and synbiotics supplementation and excluded studies that used only prebiotics. Comparison groups included placebo or standard of care.

Each of the above interventions (i.e., vitamin A, dextrose, or probiotics) was summarized separately, and the interventions were not compared to each other directly or indirectly.

#### 4.1.4 Types of outcome measures

##### Primary outcomes

The primary outcomes were:

1. All‐cause neonatal mortality (death between 0 and 28 days of life)
2. All cause infant mortality at 6 months (death between 0 days and 6 months of life)
3. All‐cause infant mortality at 12 months (death between 0 days and 12 months life).

We anticipated that studies might not report the outcomes in the follow‐up period mentioned above for the primary outcomes. If a study did not report mortality outcomes at day 28, 6 months, or 12 months, we contacted authors for data for the same. If segregated data were not available from authors, we included mortality data as follows: mortality in the first 6 weeks of life was included as neonatal mortality at day 28; between 3 and 6 months was included as 6 months, and between 9 and 12 months was included as 12 months. If the follow up was not clear, we included the mortality data at the longest follow‐up.

##### Secondary outcomes

The secondary outcomes included:

1. Sepsis‐specific mortality measured between 0 and 28 days, 0 days and 6 months, and 0 days and 12 months of life
2. Neonatal sepsis (as defined by authors) in the first 6 weeks of life
3. NEC as defined by authors
4. VAD
5. Prevention of Hypoglycemia (as defined by authors) during the neonatal period
6. Treatment of Hypoglycemia (recurrence of hypoglycaemia after the episode treated)
7. Any adverse reactions during the intervention period
8. Serious adverse events
9. Neurodevelopmental outcomes at 12 and 24 months and at the longest follow‐up.

The term neurodevelopment is a composite term that refers to cognitive, neurologic, and/or sensory outcomes. The term neurodevelopment may include intellectual disability as measured on the Mental Developmental Index of the Bayley Scales of Infant Development; gross motor delay measured on Gross Motor Function Classification System, and hearing and vision loss requiring amplification devices.

In order to be eligible for inclusion in the review, a study should have reported at least one of the primary or secondary outcomes. This was assessed at the full‐text review stage.

###### Duration of follow‐up

We included all participants in eligible studies that had outcomes of interest measured. There were no restrictions based on the duration of exposure, duration of follow‐up, or timing of the outcome measurement. If the duration of treatment exceeded the neonatal period (i.e., 28 days), we considered another 2 weeks maximum but did not include studies in which the treatment went beyond 6 weeks of supplementation. We included mortality outcomes measured at 28 days, 6 months, 12 months of life, and at the longest follow‐up as reported by authors.

###### Type of settings

We included studies conducted in LMIC. Low‐income countries were defined as those with a gross national income (GNI) per capita of USD 1005 or less in 2016, and middle‐income economies were those with a GNI per capita between USD 1006 and 3955 in 2016 (World Bank, 2017).

### 4.2 Search methods for identification of studies

The identification of studies included various methods, such as electronic and other sources. We did not exclude any based on the outcome at the screening stages.

#### 4.2.1 Electronic searches

The electronic search for relevant studies was done in the following databases: PubMed, CINAHL, LILACS, SCOPUS, and CENTRAL (Cochrane Central Register for Controlled Trials).

Appendix 1 gives the search strategy for PubMed, CINAHL, LILACS, SCOPUS, and CENTRAL. It includes keywords and MeSH terms as appropriate. This approach includes a search strategy for the population (neonates) and interventions of interest. We planned to run searches for each intervention separately. We first ran the search for the population, which is the same for each intervention. Then we ran the search for each intervention. We then combined both searches by using “AND” and kept the searches in a separate EndNote file.

An example of a search strategy for vitamin A for PubMed was as follows:

(((((“Vitamin A”[Mesh]) OR (Vitamin A[tiab] OR Aquasol A[tiab] OR Retinol[tiab] OR All Trans Retinol[tiab] OR All‐Trans‐Retinol[tiab] OR Vitamin A1[tiab] OR Vitamin A 1[tiab] OR 11‐cis‐Retinol[tiab] OR 11 cis Retinol[tiab] OR Tretinoin[tiab])AND Supplement*[tiab]))AND ((“Infant”[Mesh] OR “Premature Birth”[Mesh]) OR (Neonat*[tiab] OR neo nat*[tiab]) OR (newborn* OR new Born*[tiab] OR newly born*[tiab]) OR (preterm[tiab] OR preterms[tiab] OR pre term[tiab] OR pre terms[tiab]) OR (premature*[tiab] AND (birth*[tiab] OR born[tiab] OR deliver*[tiab])) OR (low[tiab] AND (birthweight*[tiab] OR birth weight*[tiab])) OR (lbw[tiab] OR vlbw[tiab] OR elbw[tiab]) OR infant*[tiab] OR (baby[tiab] OR babies[tiab])))) NOT (“Animals”[Mesh] NOT (“Animals”[Mesh] AND “Humans”[Mesh]))

We applied restriction of “humans” to searches. We did not apply any restrictions on searches based on outcomes, study design, or language. There was no restriction on date of publication.

The searches were conducted for vitamin A on December 10, 2018 (updated on November 13, 2019); probiotics on February 8, 2019 (updated on November 27, 2019); and dextrose on April 25, 2019 (updated on November 26, 2019).

#### 4.2.2 Searching other resources

Other resources included the search for ongoing trials at www.clinicaltrials.gov and WHO's ICTRP trials database. We also searched websites of international agencies such as WHO (including WHO's Reproductive Health Library), UNICEF, Global Alliance for Improved Nutrition (GAIN), International Food Policy Research Institute, International Initiative for Impact Evaluation (3ie), Nutrition International (NI), World Bank, USAID and USAID affiliates (e.g., FANTA, SPRING), and the World Food Programme.

Grey literature search sources included NI, GAIN, International Food Policy and Research Institute (IFPRI), and the WHO library database (WHOLIS).

We searched the reference lists of all included studies. We did citation searches of included studies in Google Scholar and Web of Science. We also searched the reference sections of previously published systematic reviews and the latest published studies. We contacted the experts and authors of the newest published studies to ask about any additional studies. Duplicates were removed.

### 4.3 Data collection and analysis

#### 4.3.1 Selection of studies

Two authors independently screened titles/abstracts using prespecified inclusion/exclusion criteria. A full text was reviewed for the studies selected in the initial screening, and the same inclusion/exclusion criteria were applied. If there was a conflict about the inclusion of a study between the two reviewers, a third reviewer (ZAB) was consulted. We used a web‐based software “Covidence” (Covidence, 2019) to do both title/abstract and full‐text screening. This software allows simultaneous independent screening of studies, and inter‐reviewer reliability can be assessed by checking the number of conflicts in the resolved conflict page following each stage of screening.

##### Description of methods used in primary research

We expected that the majority of the included studies would be randomized or cluster‐randomized. We extracted the information on study design explicitly and made a careful differentiation between experimental and observational studies. We aimed to analyze randomized and nonrandomized studies separately.

##### Criteria for determination of independent findings

We anticipated that authors might report the results of a study in multiple publications. We coded such trials as a single study to avoid double counting of the data and included all the relevant outcomes decided a priori for this review. If a pilot study was done before the larger study, we included the two studies separately unless the data from the pilot study was included in the main trial. When a clinical trial registration number was available for a study, we searched that number on PubMed to locate all the published studies linked to that trial number.

#### 4.3.2 Data extraction and management

##### Details of study coding categories

The data from included studies were abstracted into a standardized data abstraction form by two authors. We extracted data in duplicates, and any discrepancies were resolved by discussion firs. A third reviewer (ZAB) was consulted if the conflict existed after the initial discussion.

The data extraction sheet had the following information.

- General study information: authors, publication year, study design
- Study setting: World Bank region, country, World Bank income level, city/town, urban/urban slum/rural/mixed setting, duration of data collection, date of data collection
- Study population: sample size recruited, sample size analysed, female (%), description of participants (i.e., inclusion/exclusion criteria applied to recruitment)
- Intervention characteristics: type of intervention, duration of intervention, unit of randomization (where applicable), dose, frequency of provision, duration of follow up, attrition rate
- Quality assessment

Each quantitative outcome sheet contained the following:

- Subgroup (if applicable)
- Subgroup sample size
- Outcome type
- Outcome units
- Outcomes

a. Outcome measure treatment group
b. Outcome measure comparison group
c. Standard deviation

- Effect size:

a. Effect measure
b. 95% CI

#### 4.3.3 Assessment of ROB in included studies

We used the Cochrane ROB tool (Higgins & Green, 2011) for randomized studies. The Cochrane ROB tool includes the following items:

- Selection bias: random sequence generation and allocation concealment
- Performance bias: blinding of participants and personnel
- Detection bias: blinding of outcome assessment
- Attrition bias: incomplete outcome data
- Reporting bias: selective reporting
- Other sources of bias

Two authors independently performed the ROB assessments for each study. A third reviewer was involved to resolve any disagreements (ZAB). An overall score was not provided.

#### 4.3.4 Measures of treatment effect

We performed a meta‐analysis for the synthesis of quantitative data when the included studies had comparable participants, interventions, and outcomes. We did not assess the effect on outcome across the interventions, such as is done in network meta‐analysis. Each intervention was analysed separately. We analysed continuous and dichotomous data separately. For dichotomous outcomes, results were presented as summary risk ratios with 95% CI. We combined risk ratios (events per child) and rate ratios (events per child year) for incidence data because of their similar interpretation and scale. For continuous outcomes, we presented the summary results as the mean difference with 95% CI when data were available on the same scale across the studies. We used the standardized mean difference with 95% CI when data were presented in different scales across the studies.

To avoid reviewer bias, we planned to predetermine the preference for specific data for certain outcomes. For example, for mortality outcomes, we gave preference to denominators in the following order: number with the definite outcome known, number randomized, and child‐years. For morbidity data such as neonatal sepsis where both survivors and nonsurvivors might have contributed data, we gave preference to child years, number with the definite outcome known, and number randomized. For randomized trials, we gave preference to data that required the least manipulation by authors or inference by reviewers. We extracted the raw values (e.g., means and *SD*s) and built the intention‐to‐treat (ITT) analysis where applicable.

We anticipated that cause‐specific morbidity or mortality data might not be readily available, as febrile illness due to respiratory, urinary, or central nervous system infection during the neonatal period are often categorized under a broader term of neonatal sepsis (WHO, 2017b).

#### 4.3.5 Unit of analysis issues

As we planned to include multiple interventions, all interventions and outcomes within those interventions, were meta‐analyzed separately.

For randomized trials, we meta‐analyzed individual and cluster‐randomized trials in the same analysis. We assessed analyses in the cluster‐randomized trials to ensure that clustering was appropriately accounted for within the analysis of the primary study, such that study precision was not over or under‐estimated within our analysis. If the authors adjusted for cluster randomization, no further adjustment was made. In case a cluster‐randomized study was not adjusted by primary authors, we adjusted effect estimates by using the mean cluster size (*M*) and the intra‐cluster correlation coefficient (ICC) to calculate the design effect as follows: design effect = 1 + (*M* − 1) ICC. We then used the design effect to adjust the study data such that a trial was reduced to its effective sample size or standard error of the summary estimate was inflated. We used the ICC given in the published studies. If the ICC was not available from the published study, we contacted the authors for the same. If the ICC was not available from the authors, we used ICC from the similar studies done in the similar region and on a similar population or took it from the previously published reviews (Haider et al., 2017).

##### Multiple‐arm trials

We included studies with multiple intervention arms, but we only included the arms that were eligible for the review. We selected one pair (with appropriate intervention and control group) that satisfied the inclusion criteria of the review and excluded the rest. In case there were more than two groups eligible for inclusion, we combined these groups into a single pair‐wise comparison. In multiple‐arm trials using two different doses of the same intervention, we combined the two groups to avoid double counting the participants in the control group.

#### 4.3.6 Dealing with missing data

Any missing data were noted including loss to follow‐up and dropouts. The reasons for the missing data were taken from the studies, and if it was not mentioned in the studies, the authors were contacted for the same. If the authors reported the adjusted values for missing data, we used the adjusted values.

#### 4.3.7 Assessment of heterogeneity

Statistical heterogeneity was assessed using *τ* ^2^, *I* ^2^, and significance of the *χ* ^2^ test. We also assessed statistical heterogeneity by visually inspecting the forest plots.

#### 4.3.8 Assessment of reporting biases

A funnel plot and its symmetry were used to assess publication bias if the number of included studies for intervention was more than 10. If the funnel plot was suggestive of publication bias, we further investigated the publication bias with the use of Egger's test (Higgins & Green, 2011).

#### 4.3.9 Data synthesis

##### Synthesis procedures and statistical analysis

We used the software Review Manager 5.3 (Review Manager, 2019) to conduct the statistical analysis. For randomized trials, we followed the ITT analysis. If ITT was not available, and the author reported the analyses as specified in the protocol, we reconstructed the data to create an ITT analysis.

We used a random‐effect model to account for expected heterogeneity in the intervention, comparisons, or setting within studies included in a given synthesis. We used the generic inverse variance method of meta‐analysis for fixed effect models and random effect models. This method of meta‐analysis gives weight to studies based on their variance in a way that a study with low variance gets a high weight and vice versa.

We interpreted the results of the meta‐analysis based on *p* value at the 95% confidence level (a value <0.05 was considered statistically significant) and reported both significant and nonsignificant results. For subgroup analysis, we used an interaction test to determine if there was a relevant difference in effect across subgroups.

We assessed the quality of overall evidence using the GRADE approach. This method of quality assessment considers study type, within‐study ROB (methodological quality), directness of evidence, heterogeneity, precision of effect estimates, and risk of publication bias (Guyatt et al., 2011). We rated the quality of the body of evidence for each key outcome as “high,” “moderate,” “low,” or “very low.”

#### 4.3.10 Subgroup analysis and investigation of heterogeneity

##### Neonatal vitamin A supplementation

Although we had planned a number of subgroup analyses for neonatal vitamin A supplementation; however, a recent IPD analysis (West et al., 2019) covered both individual and study level subgroup analyses, so we did not perform any subgroup analysis for vitamin A supplementation at this stage

##### Neonatal probiotic supplementation

1. Gestational age: term and preterm
2. Strains used in probiotics: single strain versus multiple strain and of type of strain used in each probiotic
3. Strains used in probiotics: contains *Lactobacillus* versus *Bifidobacterium* versus both
4. Settings: community‐based versus hospital setting
5. Type of feedings: breastmilk versus formula milk versus mixed.

##### Oral dextrose gel supplementation

1. Gestational age: term and postterm versus late preterm (35–36 weeks) versus moderately preterm (30–34 weeks) versus extremely preterm (<30 weeks)
2. Dose: equal or <200 mg/kg versus >200 mg/kg
3. Frequency: one versus more than one dose
4. Time of administration: ≤1 h of age versus after 1 h of age versus after 2 h of age.

#### 4.3.11 Sensitivity analysis

1. High quality studies versus low quality studies. The quality of study was subjectively based on the ROB assessment. Even though we considered all the domains included in the Cochrane ROB tool, we gave higher importance to sequence generation and allocation concealment, as most of the outcomes for this review were objective, and it was less likely that the results of the included studies would have been biased by a lack of blinding.
2. Random versus fixed effect models. We chose this sensitivity analysis to assess if the summary estimates will change significantly based on use of random versus fixed effect model. There is no exact criterion to choose between the two models, and we wanted to make sure that estimates were not significantly different between the two models.

##### Treatment of qualitative research

We did not plan to include qualitative research.

#### 4.3.12 Summary of findings and assessment of the certainty of the evidence

##### Summary of findings' tables

We constructed “Summary of findings” tables for all of the primary outcomes using the Grading of Recommendations Assessment, Development and Evaluation (GRADE) criteria (GRADEpro GDT 2015). These covered consideration of within‐study ROB (methodological quality), directness of evidence, heterogeneity, precision of effect estimates and risk of publication bias. We rated the certainty of evidence for each key outcome as “high,” “moderate,” “low,” or “very low.” The GRADE evidence is described in Table 1. Nonrandomised studies were initially rated as “low” quality. If there were no serious methodological flaws, we upgraded the evidence for studies with a large magnitude of effect; presence of a dose response relationship; and effect of plausible residual confounding.

**Table 1:** Effect of probiotic supplementation during neonatal period: Subgroup analysis

Outcome or subgroup	No. of studies	Effect estimate: relative risk	Test for subgroup difference
All‐cause mortality: subgroup analysis: settings			
Hospital based	22	0.78 [0.65, 0.94]	*p* = .31
Community based	3	1.25 [0.51, 3.05]	*I* ^2^ = 0%
All‐cause mortality: subgroup analysis: type of probiotics			
Preparation contain a single strain of probiotics	9	0.80 [0.61, 1.05]	*p* = .95
Preparation contained multiple strains of probiotics	12	0.80 [0.58, 1.09]	*I* ^2^ = 0%
Preparation contained synbiotics (prebiotics + probiotics)	5	0.69 [0.29, 1.61]	
All‐cause mortality: subgroup analysis: type of participants			
Study include preterm/low birth weight babies	24	0.79 [0.65, 0.95]	*p* = .47
Study included term infants only	1	1.38 [0.31, 6.08]	*I* ^2^ = 0%
All‐cause mortality: subgroup analysis: type of feedings			
Baby received breastmilk only	14	0.81 [0.62, 1.05]	*p* = .44
Baby received formula milk only	1	1.38 [0.31, 6.08]	
Baby received both both breastmilk and formula milk	8	0.69 [0.48, 0.99]	*I* ^2^ = 0%
Type of feeding was unclear	3	1.33 [0.63, 2.81]	
All‐cause mortality: subgroup analysis: probiotics preparation			
Preparation contained *Lactobacillus*	10	0.82 [0.63, 1.05]	*p* = .47
Preparation contained *Bifidobacterium*	1	0.43 [0.17, 1.09]	
Preparation contained both *Lactobacillus* and *Bifidobacterium*	13	0.71 [0.47, 1.08]	*I* ^2^ = 0%
Preparation contained *Saccharomyces boulardii* only	2	1.12 [0.46, 2.71]	
Necrotizing enterocolitis: subgroup analysis: probiotic preparation			
Preparation contained *Lactobacillus*	13	0.39 [0.25, 0.61]	*p* = .05
Preparation contained *Bifidobacterium*	1	0.20 [0.09, 0.47]	
Preparation contained both *Lactobacillus* and *Bifidobacterium*	14	0.49 [0.36, 0.68]	
			*I* ^2^ = 60.5%
Preparation contained *S. boulardii* only	2	0.94 [0.45, 1.95]	
Necrotizing enterocolitis: subgroup analysis: type of feeding			
Baby received breastmilk only	13	0.43 [0.31, 0.59]	*p* = .74
Baby received formula only	1	0.21 [0.03, 1.76]	
Baby received both breastmilk and formula milk	9	0.55 [0.33, 0.92]	*I* ^2^ = 0%
Type of feeding was unclear	7	0.41 [0.17, 1.00]	
Necrotizing enterocolitis: subgroup analysis: type of probiotics			
Preparation contained a single strain of probiotics	12	0.48 [0.30, 0.76]	*p*= .50
Preparation contained multiple strains of probiotics	15	0.48 [0.35, 0.67]	*I* ^2^ = 0%
Preparation contained synbiotics (prebiotics + probiotics)	3	0.28 [0.12, 0.67]	
Neonatal sepsis: subgroup analysis: probiotic preparation			
Preparation contained *Lactobacillus*	11	0.74 [0.62, 0.87]	*p* = .79
Preparation contained *Bifidobacterium*	1	0.81 [0.60, 1.09]	
Preparation contained both *Bifidobacterium* and *Lactobacillus*	6	0.83 [0.68, 1.02]	*I* ^2^ = 0%
Preparation contained *S. boulardii* only	3	0.73 [0.57, 0.94]	
Neonatal sepsis: subgroup analysis: type of feeding			
Baby received breastmilk only	8	0.71 [0.61, 0.83]	*p* = .04
Baby received formula milk only	2	0.59 [0.22, 1.56]	
Baby received both formula and breastmilk only	6	0.77 [0.65, 0.90]	*I* ^2^ = 65%
Type of feeding was unclear	4	0.95 [0.82, 1.09]	
Neonatal sepsis: type of probiotics			
Preparation contained single strain of probiotics	8	0.84 [0.74, 0.96]	*p* = .21
Preparation contained multiple strains of probiotics	9	0.81 [0.68, 0.97]	
			*I* ^2^ = 35%
Preparation contained synbiotics (prebiotics + probiotics)	4	0.67 [0.54, 0.83]	
Neonatal sepsis: subgroup analysis: settings			
Hospital based	19	0.83 [0.76, 0.91]	*p* = .19
Community based	2	0.67 [0.49, 0.91]	*I* ^2^ = 42%

We used GRADE and prepared the summary of findings tables for the following primary outcomes:

- Stillbirth defined as baby born with no signs of life at or after 28 weeks' gestation
- Perinatal mortality (stillbirth and deaths ≤7 days)
- Neonatal mortality (death < 28 days)
- Infant mortality (deaths between 0 and 12 months)
- Under‐five mortality (deaths between 0 and 59 months)
- Miscarriage
- Mean maternal body mass index

## 5 RESULTS

### 5.1 Description of studies

#### 5.1.1 Results of the search

**Figure 1:**
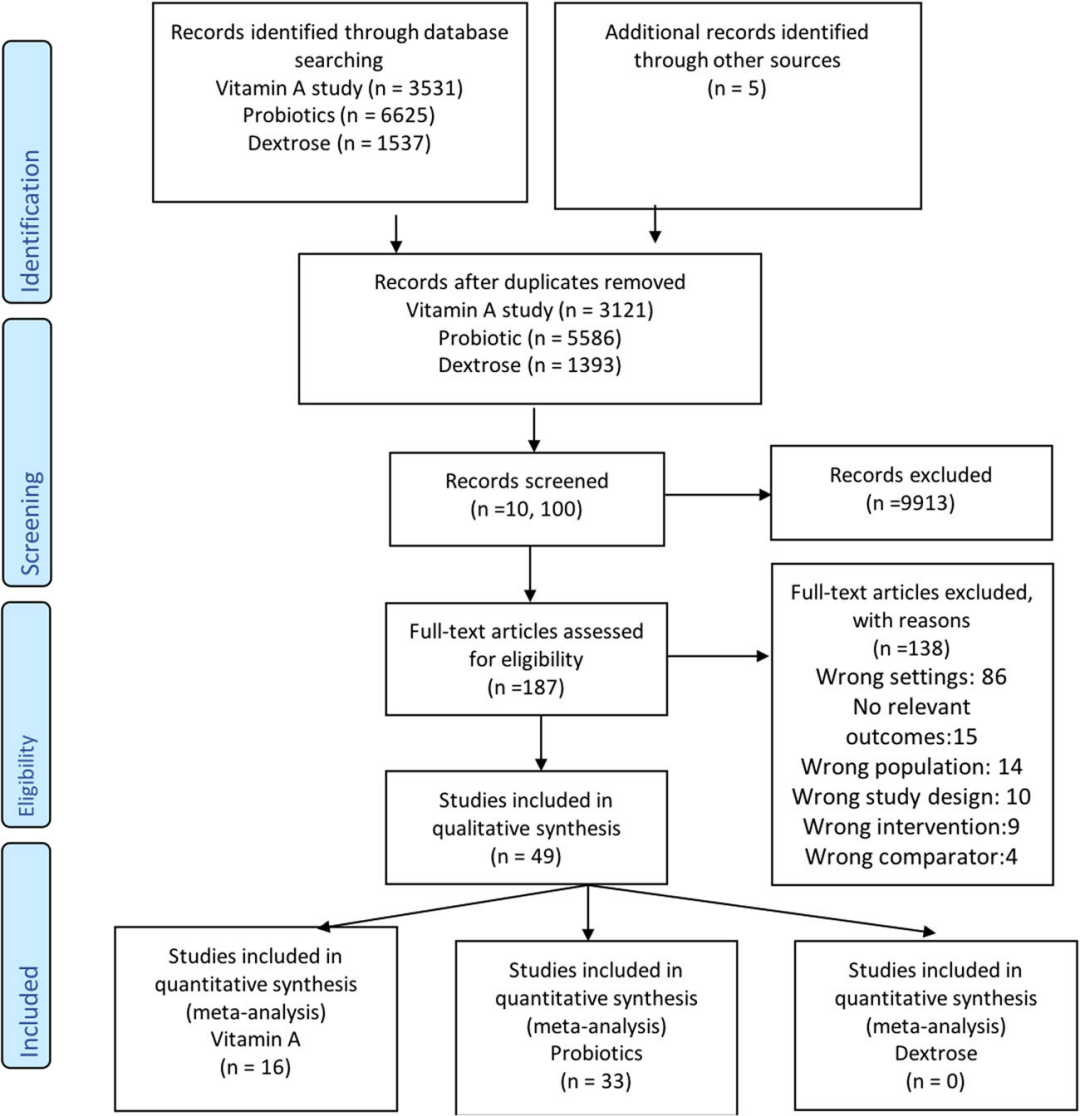
PRISMA flow diagram

Figure 1 shows the PRISMA flow diagram for our literature search.

##### Vitamin A supplementation during neonatal period

Sixteen studies reported in 45 publications assessed the effect of neonatal vitamin A supplementation (Ahmad et al., 2019; Basu et al., 2019; Benn et al., 2008, 2010, 2014; Edmond et al., 2015; Giridhar et al., 2019; Humphrey et al., 1996; Klemm et al., 2008; Malaba et al., 2005; Masanja et al., 2015; Mazumder et al., 2015; Rahmathullah et al., 2003; Soofi et al., 2017; Sun et al., 2019; West et al., 1995). These studies included a total of about 16,366 participants. All the studies were RCTs and published in a peer‐reviewed journal.

##### Dextrose gel supplementation during neonatal period

We did not identify any studies that assessed the use of dextrose gel supplementation during neonatal period for prevention or treatment of hypoglycaemia in LMIC.

##### Probiotic supplementation during neonatal period

Thirty‐three studies reported in 37 publications evaluated the effect of probiotic supplementation during the neonatal period and included a total of 11,595 participants (probiotics, 5854 and controls, 5741; Amini et al., 2017; Braga et al., 2011; Chowdhury et al., 2016; Cooper et al., 2017; Cui et al., 2019; Dashti et al., 2014; Demirel et al., 2013; Dilli et al., 2015; Dongol Singh et al., 2017; Dutta et al., 2015; Fernández‐Carrocera et al., 2013; Hariharan et al., 2016; Hernández‐Enríquez et al., 2016; Huaxian, 2013; Hussain et al., 2016; Kaban et al., 2019, Mazumder et al., 2015; Nandhini et al., 2016; Guney‐Varal et al., 2017; Oncel et al., 2014; Panigrahi et al., 2017; Rojas et al., 2012; Roy et al., 2014; Samanta et al., 2009; Shashidhar et al., 2017; Sinha et al., 2015; Sari et al., 2011; Serce et al., 2013; Shadkam et al., 2015; Saengtawesin et al., 2014; Tewari et al., 2015; Xu et al., 2016).

Three of the studies were available in the form of abstracts (Hariharan et al., 2016; Huaxian, 2013; Rehman et al., 2018). The rest of the studies were published in a peer‐reviewed journal.

#### 5.1.2 Included studies

The characteristics of included studies are available in the table Characteristics of included studies.

##### Vitamin A supplementation during neonatal period

###### Type of studies

All the included studies were RCTs. Thirteen studies were individually randomized (Ahmad et al., 2019; Basu et al., 2019; Benn et al., 2008, 2010, 2014; Edmond et al., 2015; Giridhar et al., 2019; Humphrey et al., 1996; Malaba et al., 2005; Masanja et al., 2015; Mazumder et al., 2015; Rahmathullah et al., 2003), and three studies were cluster‐randomized (Klemm et al., 2008; Soofi et al., 2017; Sun et al., 2019; West et al., 1995). Three trials had multiple arms of interventions (Benn et al., 2010, 2014; Malaba et al., 2005).

###### Country

Studies were conducted in 10 different countries with four studies conducted in India (Basu et al., 2019; Giridhar et al., 2019; Mazumder et al., 2015; Rahmathullah et al., 2003), three studies in Guinea‐Bissau (Benn et al., 2008, 2010, 2014), two studies in Bangladesh (Ahmad et al., 2019; Klemm et al., 2008), and one each in China (Sun et al., 2019), Ghana (Edmond et al., 2015), Indonesia (Humphrey et al., 1996), Nepal (West et al., 1995), Pakistan (Soofi et al., 2017), Tanzania (Masanja et al., 2015), and Zimbabwe (Malaba et al., 2005).

###### Settings

Thirteen studies were conducted in the community setting, while three studies were conducted in the hospital setting (Basu et al., 2019; Giridhar et al., 2019; Sun et al., 2019).

###### Participants

Most of the studies included live born infants who were otherwise healthy. One study included only low birth weight babies (Benn et al., 2010), two studies included newborns with very low birth weight (Basu et al., 2019; Giridhar et al., 2019), and one study included extremely premature babies (Sun et al., 2019). The sample size of each study ranged from 120 (Giridhar et al., 2019) to 44,948 (Mazumder et al., 2015).

###### Dose

Most of the included studies for the use of neonatal vitamin A supplementation used a dose of 50,000 IU. Rahmathullah et al. (2003) gave 24,000 IU daily, and Benn et al. (2010) used 25,000 IU. Benn et al. (2014) compared doses of 50,000 IU versus 25,000 IU. Basu et al. (2019) used a daily dose of 1,500 IU.

###### Comparison

In all the included studies for neonatal vitamin A supplementation a placebo was given to the control group.

##### Probiotic supplementation during neonatal period

###### Type of studies

All the studies that evaluated the effect of probiotic supplementation during the neonatal period were individual RCTs. Two studies had multiple intervention groups (Dilli et al., 2015; Dutta et al., 2015). One of these studies compared different combinations of probiotics with prebiotics (Dilli et al., 2015), and the other study compared different doses of probiotics (Dutta et al., 2015). For the study by Dilli et al. (2015), we included the data in a way that the only difference between the two groups was probiotics. For the study by Dutta et al. (2015), we combined all the groups that compared different doses and compared them with the placebo to avoid double‐counting of the placebo group data.

One study included neonates with and without exposure to human immunodeficiency virus (HIV; based on maternal history of HIV). We included the data for these groups separately in the meta‐analysis (Niekerk et al., 2015 (HIV exposed); Niekerk et al., 2015 (HIV nonexposed).

###### Country

Studies were conducted in 13 different countries with 10 studies conducted in India (Dutta et al., 2015; Hariharan et al., 2016; Mazumder et al., 2015; Nandhini et al., 2016; Panigrahi et al., 2017; Roy et al., 2014; Samanta et al., 2009; Shashidhar et al., 2017; Sinha et al., 2015; Tewari et al., 2015), six studies in Turkey (Demirel et al., 2013; Dilli et al., 2015; Guney‐Varal et al., 2017; Oncel et al., 2014; Sari et al., 2011; Serce et al., 2013), three studies each in China (Cui et al., 2019; Huaxian, 2013; Xu et al., 2016) and Iran (Amini et al., 2017; Dashti et al., 2014; Shadkam et al., 2015), two each in Mexico (Fernández‐Carrocera et al., 2013; Hernández‐Enríquez et al., 2016) and South Africa (Cooper et al., 2017; Niekerk et al., 2015 (HIV exposed)), and one each in Bangladesh (Chowdhury et al., 2016), Brazil (Braga et al., 2011), Colombia (Rojas et al., 2012), Indonesia (Kaban et al., 2019), Nepal (Dongol Singh et al., 2017), Pakistan (Hussain et al., 2016), and Thailand (Saengtawesin et al., 2014).

###### Settings

All the studies were conducted in the hospital setting except for three studies where participants were followed in the community setting (Cooper et al., 2017; Dongol Singh et al., 2017; Panigrahi et al., 2017).

###### Participants

Only one study (Cooper et al., 2017) included neonates that were full term. The rest of the studies included participants that were either low birth weight, preterm, or both. The participants were recruited from neonatal intensive care units except in three studies (Cooper et al., 2017; Dongol Singh et al., 2017; Panigrahi et al., 2017), where participants were recruited from the community.

###### The intervention

Thirteen studies used a single strain of probiotics (Cui et al., 2019; Demirel et al., 2013; Dongol Singh et al., 2017; Hernández‐Enríquez et al., 2016; Hussain et al., 2016; Kaban et al., 2019; Oncel et al., 2014; Rojas et al., 2012; Roy et al., 2014; Serce et al., 2013; Shadkam et al., 2015; Tewari et al., 2015; Xu et al., 2016), and 13 studies used a preparation that contained multiple strains of probiotics (Amini et al., 2017; Braga et al., 2011; Chowdhury et al., 2016; Dashti et al., 2014; Dutta et al., 2015; Fernández‐Carrocera et al., 2013; Hariharan et al., 2016; Niekerk et al., 2015 (HIV exposed); Roy et al., 2014; Saengtawesin et al., 2014; Samanta et al., 2009; Shadkam et al., 2015; Sinha et al., 2015). Five studies used a preparation that had a probiotic + prebiotic (synbiotic) (Cooper et al., 2017; Dilli et al., 2015; Guney‐Varal et al., 2017; Nandhini et al., 2016; Panigrahi et al., 2017); among these five studies, three studies (Cooper et al., 2017; Dilli et al., 2015; Panigrahi et al., 2017) used a probiotic preparation that had a single strain of bacteria, and the other two studies used a preparation that had multiple strains of bacteria (Guney‐Varal et al., 2017; Nandhini et al., 2016). One study did not report the strain of probiotic supplementation (Huaxian, 2013).

Ten studies used a probiotic preparation that contained *Lactobacillus* (Cooper et al., 2017; Cui et al., 2019; Dongol Singh et al., 2017; Hernández‐Enríquez et al., 2016; Kaban et al., 2019; Oncel et al., 2014; Panigrahi et al., 2017; Rojas et al., 2012; Roy et al., 2014; Shadkam et al., 2015), and two studies used a preparation that contained *Bifidobacterium* (Dilli et al., 2015; Hussain et al., 2016). Fourteen studies used a preparation that had both *Lactobacillus* and *Bifidobacterium* (Amini et al., 2017; Braga et al., 2011; Chowdhury et al., 2016; Dashti et al., 2014; Fernández‐Carrocera et al., 2013; Guney‐Varal et al., 2017; Hariharan et al., 2016; Nandhini et al., 2016; Niekerk et al., 2015 (HIV exposed); Roy et al., 2014; Saengtawesin et al., 2014; Samanta et al., 2009; Shashidhar et al., 2017; Sinha et al., 2015). Three studies used *Saccharomyces boulardii* (Demirel et al., 2013; Serce et al., 2013; Xu et al., 2016), and one study used *Bacillus clausii* (Tewari et al., 2015).

The probiotics were mostly given with breastmilk or formula feedings and started when the baby was able to tolerate minimal enteral feeds. The duration and dose of probiotic supplementation varied among the studies.

###### Comparison

Sixteen studies used a placebo (Cui et al., 2019; Dashti et al., 2014; Demirel et al., 2013; Dilli et al., 2015; Dongol Singh et al., 2017; Dutta et al., 2015; Kaban et al., 2019; Niekerk et al., 2015 (HIV exposed); Oncel et al., 2014; Panigrahi et al., 2017; Rojas et al., 2012; Roy et al., 2014; Serce et al., 2013; Shadkam et al., 2015; Sinha et al., 2015; Tewari et al., 2015); the rest of the studies used a control group receiving standard of care only.

###### Outcomes

All the studies reported data for at least one outcome that could be included in the meta‐analysis. Twenty five studies reported data for all‐cause mortality, 29 studies reported data for NEC, and 21 studies reported data for the incidence of neonatal sepsis. See Section 5.3 for more details.

#### 5.1.3 Excluded studies

Overall, 138 studies were excluded. See the Characteristics of excluded studies for reasons for exclusion of studies.

Among excluded studies, 86 studies were excluded because of the wrong settings, and most of these studies were conducted in high‐income countries. Fifteen studies were excluded because no relevant clinical outcomes were available from the abstract or full text of the studies. Fourteen studies were excluded for the wrong population, and 10 studies were excluded because of the wrong study design. Nine studies had a wrong intervention, and four studies had a wrong comparator.

### 5.2 ROB in included studies

#### 5.2.1 Vitamin A supplementation during the neonatal period

**Figure 2:**
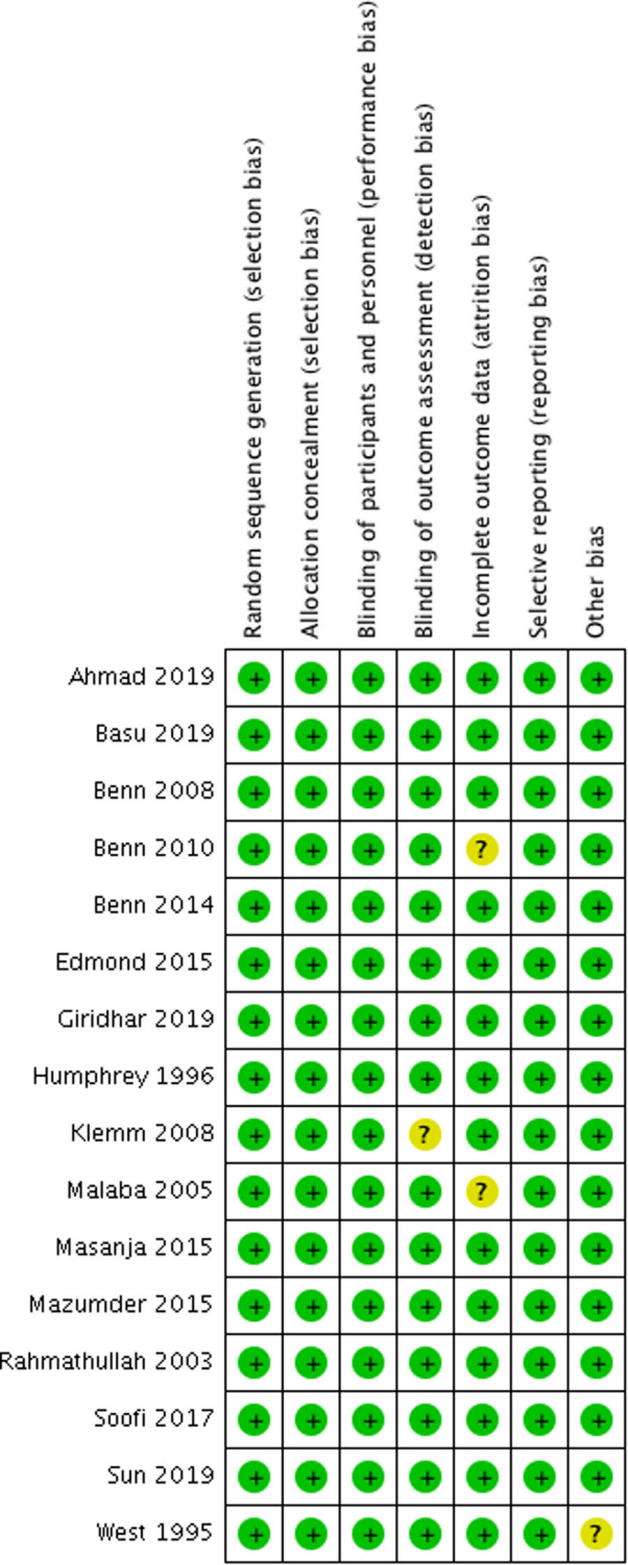
Risk of bias: neonatal vitamin A supplementation

Figure 2 shows the ROB in the 16 included studies that addressed vitamin A supplementation during the neonatal period.

#### 5.2.2 Probiotic supplementation during the neonatal period

**Figure 3:**
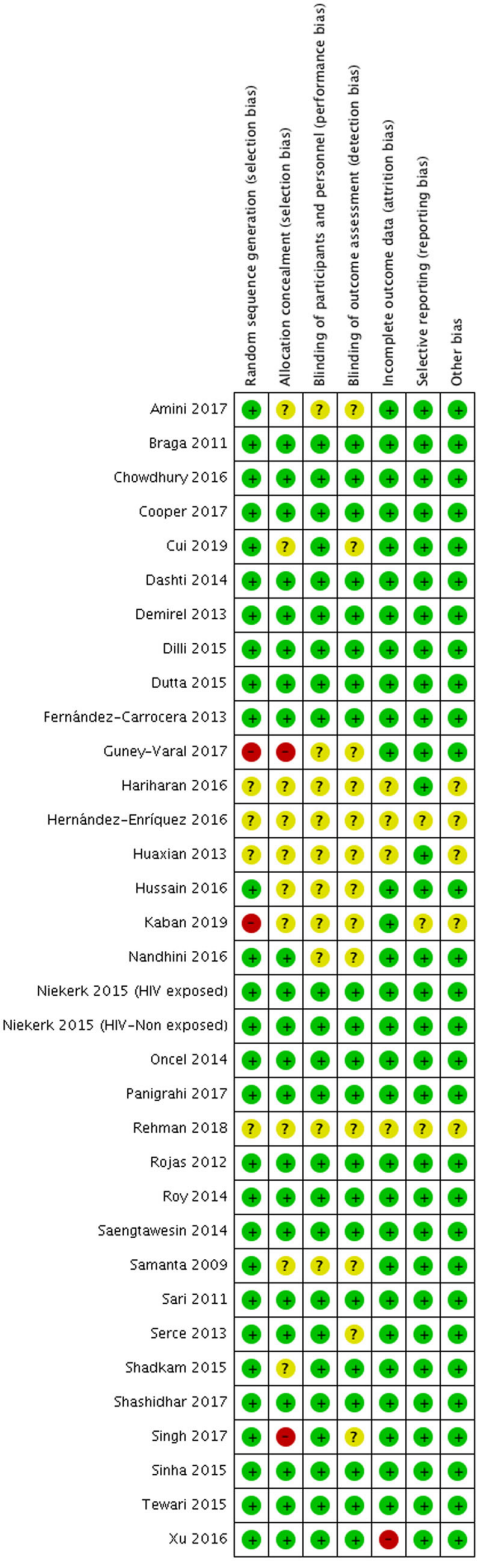
Risk of bias: probiotic supplementation during neonatal period

Figure 3 show the ROB in the 33 included studies that addressed probiotic supplementation during the neonatal period.

#### 5.2.3 Allocation (selection bias)

##### Vitamin A supplementation during the neonatal period

All the studies for vitamin A supplementation were at low ROB for sequence generation and allocation concealment.

##### Probiotic supplementation during the neonatal period

Two studies were judged to be at high ROB due to inadequate randomization (Guney‐Varal et al., 2017; Kaban et al., 2019), and four studies did not provide enough information to allow a judgment about methods of randomization; these were labelled as having an unclear ROB (Hariharan et al., 2016; Hernández‐Enríquez et al., 2016; Huaxian, 2013; Rehman et al., 2018). The rest of the studies had a low ROB for sequence generation.

Two studies were considered at high ROB due to inability to conceal the allocation (Dongol Singh et al., 2017; Guney‐Varal et al., 2017). Ten studies had an unclear ROB, as these studies did not provide enough information to assess methods of allocation concealment (Amini et al., 2017; Cui et al., 2019; Hariharan et al., 2016; Hernández‐Enríquez et al., 2016; Huaxian, 2013; Hussain et al., 2016; Kaban et al., 2019, Rehman et al., 2018; Samanta et al., 2009; Shadkam et al., 2015). The rest of the studies had a low ROB for allocation concealment.

#### 5.2.4 Blinding (performance bias and detection bias)

##### Vitamin A supplementation during neonatal period

None of the included studies for neonatal vitamin A supplementation was at increased ROB for blinding.

##### Probiotic supplementation during neonatal period

Ten studies had an unclear ROB due to blinding of the participants (Amini et al., 2017; Guney‐Varal et al., 2017; Hariharan et al., 2016; Hernández‐Enríquez et al., 2016; Huaxian, 2013; Hussain et al., 2016; Kaban et al., 2019; Nandhini et al., 2016; Rehman et al., 2018; Samanta et al., 2009). The rest of the studies had a low ROB due to the inability to do blinding of the participants.

Thirteen studies had an unclear ROB for blinding of the outcome assessors (Amini et al., 2017; Cui et al., 2019; Dongol Singh et al., 2017; Guney‐Varal et al., 2017; Hariharan et al., 2016; Hernández‐Enríquez et al., 2016; Huaxian, 2013; Hussain et al., 2016; Kaban et al., 2019; Nandhini et al., 2016; Rehman et al., 2018; Serce et al., 2013; Samanta et al., 2009). The rest of the studies had a low ROB.

#### 5.2.5 Incomplete outcome data (attrition bias)

##### Vitamin A supplementation during the neonatal period

All studies were at low risk for attrition bias except two studies that had an unclear ROB (Benn et al., 2010; Malaba et al., 2005).

##### Probiotic supplementation during the neonatal period

Most of the studies had a minimal loss to follow‐up. One study was at high ROB where more than 20% of the participants were lost to follow‐up (Xu et al., 2016). Four studies had an unclear ROB, as there was not enough information to make an assessment in these studies (Hariharan et al., 2016; Hernández‐Enríquez et al., 2016; Huaxian, 2013; Rehman et al., 2018).

#### 5.2.6 Selective reporting (reporting bias)

##### Vitamin A supplementation during neonatal period

All studies were considered to have low ROB for selective outcome reporting.

##### Probiotic supplementation during neonatal period

Most of the studies reported all relevant outcomes, and we did not consider any particular study at high ROB. Three studies had unclear ROB for selective outcome reporting (Hernández‐Enríquez et al., 2016; Kaban et al., 2019; Rehman et al., 2018).

#### 5.2.7 Other potential sources of bias

##### Vitamin A supplementation during neonatal period

No study was considered at high ROB due to other reasons.

##### Probiotic supplementation during neonatal period

No other major source of ROB was noted. Five studies had unclear ROB due to limited available information (Hariharan et al., 2016; Hernández‐Enríquez et al., 2016; Huaxian, 2013; Kaban et al., 2019; Rehman et al., 2018).

### 5.3 Effects of interventions

#### 5.3.1 VItamin A supplementation during neonatal period

##### All‐cause mortality during the neonatal period

Five studies from community settings reported the effect of vitamin A supplementation on all‐cause neonatal mortality. These combined results showed no significant difference between the intervention and the control group (RR, 0.99; 95% CI, 0.90–1.08; six studies, 126,548 participants, heterogeneity: *τ* ^2^ = 0.00; *χ* ^2^ = 3.64, (*p* = 0.46); *I* ^2^ = 0%). The grade rating for this outcome was “high.” Summary of findings Table 2.

**Table 2:** Vitamin A compared to placebo for neonatal health

Vitamin A compared to placebo for neonatal health			
Patient or population: neonates (0‐28 days)			
Setting: low and middle income countries			
Intervention: vitamin A			
Comparison: placebo			
Outcomes	Relative effect (95% CI)	No. of participants (studies)	Certainty of the evidence (GRADE)
All‐cause neonatal mortality	RR, 0.99 (0.90–1.08)	126,548 (6 RCTs)	⊕⊕⊕⊕ HIGH
All‐cause mortality at 6 months of age	RR, 0.98 (0.89–1.07)	154,940 (12 RCTs)	⊕⊕⊕⊕ HIGH
All‐cause mortality at 12 months of age	RR, 1.04 (0.94–1.14)	118,376 (8 RCTs)	⊕⊕⊕⊕ HIGH
Adverse Events: Bulging Fontanelle 48–72 h	RR, 1.53 (1.12–2.09)	100,562 (6 RCTs)	⊕⊕⊕⊕ HIGH
*The risk in the intervention group (and its 95% confidence interval) is based on the assumed risk in the comparison group and the **relative effect** of the intervention (and its 95% CI).			
**GRADE Working Group grades of evidence**			
**High certainty**: We are very confident that the true effect lies close to that of the estimate of the effect			
**Moderate certainty:** We are moderately confident in the effect estimate: The true effect is likely to be close to the estimate of the effect, but there is a possibility that it is substantially different			
**Low certainty**: Our confidence in the effect estimate is limited: The true effect may be substantially different from the estimate of the effect			
**Very low certainty**: We have very little confidence in the effect estimate: The true effect is likely to be substantially different from the estimate of effect			

Abbreviations: CI, confidence interval; OR, odds ratio; RR, risk ratio.

###### Sensitivity analysis: Fixed effect model

Use of a fixed effect model did not change the summary estimate for neonatal mortality (RR, 0.99; 95% CI, 0.90–1.08).

##### All‐cause mortality at 6 months

Twelve studies from community settings reported the data for the effect of neonatal vitamin A supplementation on all‐cause mortality at 6 months. The combined results showed no difference between the intervention and control group (RR, 0.98; 95% CI, 0.89–1.07; 12 studies, 154,940 participants; heterogeneity: *τ* ^2^ = 0.01; *χ* ^2^ = 19.14, (*p* = 0.06); *I* ^2^ = 43%). The Grade rating for this outcome was “high.” Summary of findings Table 2. A funnel plot for publication bias was symmetrical.

###### Sensitivity analysis: Fixed effect model

Use of a fixed effect model led to minimal change in the summary estimate (RR, 0.97; 95% CI, 0.91–1.03).

##### All‐cause mortality at 12 months

Eight studies from community settings reporting on the impact of neonatal vitamin A supplementation reported data for all‐cause mortality at 12 months (RR, 1.04; 95% CI, 0.94–1.14; eight studies, 118,376 participants; Heterogeneity: *τ* ^2^ = 0.01; *χ* ^2^ = 12.99, df = 7 (*p* = 0.07); *I* ^2^ = 46%]. We rated this evidence as high certainty Summary of findings Table 2.

###### Sensitivity analysis: Fixed effect model

Use of a fixed effect model did not change the summary estimate significantly (RR, 1.02; 95% CI; 0.96–1.08).

Three studies from hospital settings also reported data on mortality. The time to event for mortality was not clear in these studies. We did not pool data from the hospital‐based studies with other studies, as the community‐based studies had participants that were very different from hospital‐based studies. We briefly describe the results of these studies below.

A study by Basu et al. (2019) reported the primary outcome; this was a composite incidence of all‐cause mortality and oxygen requirement for 28 days. The results showed a reduction in mortality in the vitamin A group compared to the control group (RR, 0.44; 95% CI, 0.23–0.84).

No difference in mortality was reported by Sun et al. (2019) in the Vitamin A group versus placebo (RR, 0.49; 95% CI, 0.45–5.32).

Similarly, no difference was noted in all‐cause mortality by Giridhar et al. (2019) (RR, 2; 95% CI, 0.63–6.30).

##### Adverse outcomes: Bulging fontanelle

Six studies reported on the effect of neonatal vitamin A supplementation on incidence of bulging fontanelle. The combined results showed a 53% increased risk of bulging fontanelle in the intervention group compared to control (RR, 1.53; 95% CI, 1.12–2.09; heterogeneity: *τ* ^2^ = 0.08; *χ* ^2^ = 14.20, (*p* = 0.01); *I* ^2^ = 65%). We have high certainty in this evidence. Summary of findings Table 2.

##### Adverse outcomes: Vomiting

The combined results from six studies showed that neonatal vitamin A supplementation did not increase the risk of vomiting (RR, 1.00; 95% CI, 0.93–1.07; heterogeneity: *τ* ^2^ = 0.00; *χ* ^2^ = 3.90, (*p* = 0.42); *I* ^2^ = 0%).

##### Vitamin A deficiency

One study from the community setting (Benn et al., 2008) reported VAD at 6 weeks and 4 months post neonatal supplementation. No significant difference was noted between the two groups at 6 weeks (RR, 0.94; 95% CI, 0.75–1.19) or 4 months (RR, 1.02; 95% CI, 0.64–1.62).

Another study from the hospital setting (Giridhar et al., 2019) showed a significant decrease in VAD in the intervention group compared to control (RR, 0.09; 95% CI, 0.024–0.38).

##### Neurodevelopment outcomes

Two studies reported long‐term neurodevelopmental outcomes after use of vitamin A supplementation during the neonatal period. As the outcomes measured and duration of follow up were different, we did not pool the studies.

Humphrey et al. (1996) reported on neurodevelopmental outcomes at 3 year follow‐up after neonatal vitamin A supplementation by using Bayley Scales of Infant Development. The study authors analysed the data for children with (*n* = 91) and without (*n* = 432) bulging fontanelle who received vitamin A versus placebo. The results showed that neonatal vitamin A supplementation did not have any adverse effect on development in the presence or absence of bulging fontanelle. Neonatal vitamin A supplementation had a positive effect on all developmental scores. The reported developmental scores addressed developmental areas such as orientation‐engagement, emotional regulation, and motor quality.

Klemm et al. (2008) reported data on neurodevelopmental outcomes 8 years after vitamin A supplementation. The authors followed a cohort of participants (*n* = 1613) who either directly received neonatal vitamin A or whose mother received vitamin A during pregnancy. The results showed no significant difference in intelligence, memory, and motor function; however, when the neonates and their mothers were supplemented with vitamin A versus placebo, it increased their performance in reading, spelling, and math computation.

#### 5.3.2 Probiotic supplementation during the neonatal period

Data were available for the effect of probiotic supplementation for all‐cause mortality, NEC, sepsis, and sepsis‐specific mortality.

##### All‐cause mortality at longest follow‐up

**Figure 4:**
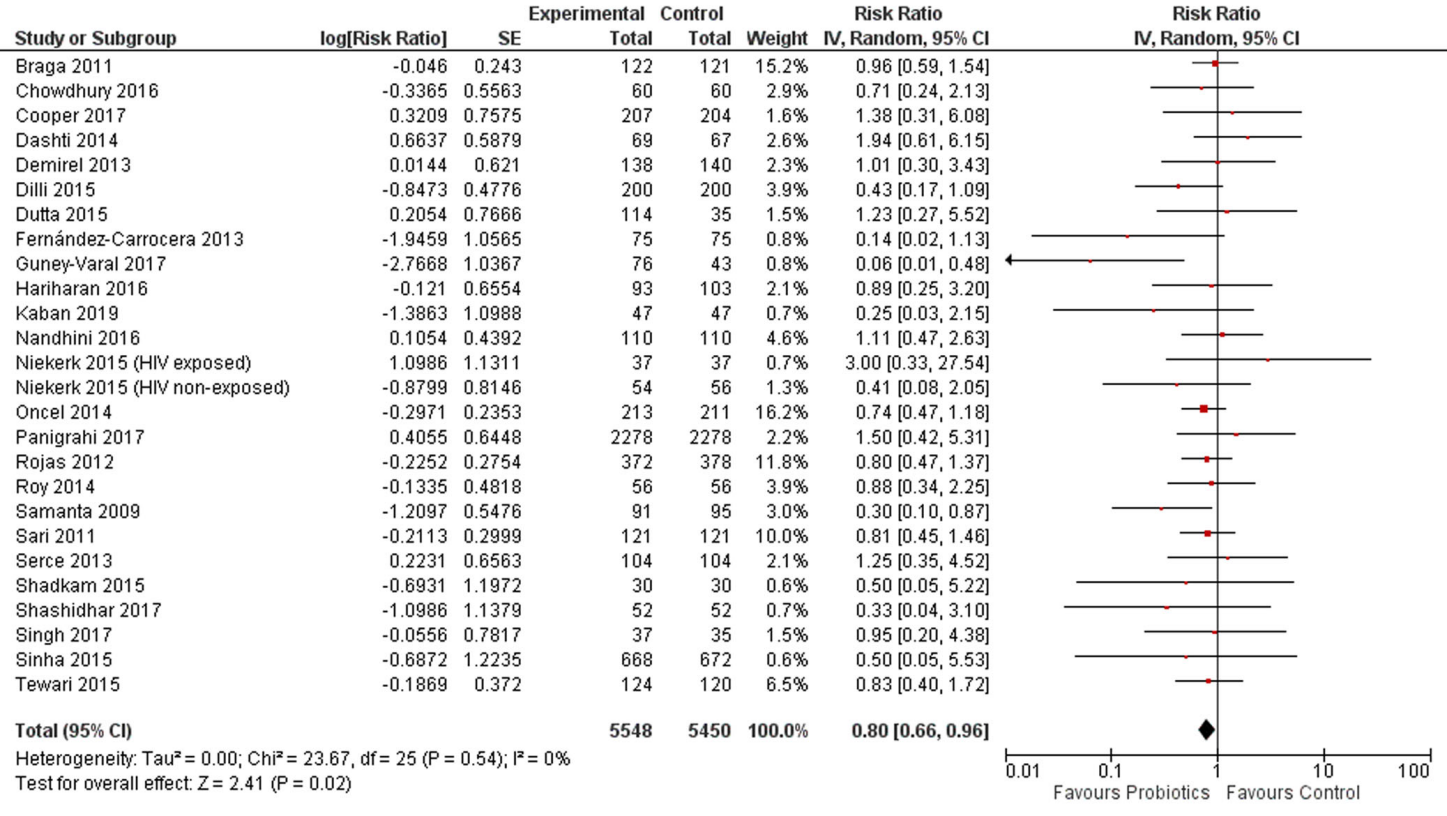
(Analysis 2.1) Forest plot of comparison: 2 probiotics versus control, outcome: 2.1 all‐cause mortality

Twenty‐five studies that included 10,998 subjects (probiotics 5548, control 5450) reported data for all‐cause mortality for the effect of probiotic supplementation on all‐cause mortality. Our meta‐analysis found a reduction of 20% in all‐cause mortality in the probiotic group compared to control (RR, 0.80; 95% CI, 0.66–0.96; heterogeneity: *τ* ^2^ = 0.00; (*p* = 0.55); *I* ^2^ = 0%) (Figure 4). The number needed to treat was 100. The GRADE rating for this outcome was “high.” Summary of findings Table 3.

**Table 3:** Probiotics supplementation during neonatal period

Probiotics supplementation compared to control during neonatal period					
Patient or population: neonates (Mmost of the included studies had preterm and low birth weight neonates)					
Setting: low and middle income countries					
Intervention: probiotics/synbiotics					
Comparison: control					
Outcomes	No. of participants (studies) Follow up	Certainty of the evidence (GRADE)	Relative effect (95% CI)	Anticipated absolute effects* (95% CI)	
				Risk with control	Risk difference with probiotics (intervention)
All‐cause mortality	10904 (25 RCTs)	⊕⊕⊕⊕ HIGH^a^, ^b^, ^c^, ^d^	RR, 0.80 (0.66–0.96)	Study population	
				47 per 1000	9 fewer per 1000 (15 fewer to 1 fewer)
Neonatal sepsis	8918 (21 RCTs)	⊕⊕⊕⊕ HIGH^a^, ^d^, ^e^	RR, 0.78 (0.70–0.86)	Study population	
				205 per 1000	45 fewer per 1000 (62 fewer to 29 fewer)
Necrotizing enterocolitis	55574 (29 RCTs)	⊕⊕⊕⊕ HIGH^a^, ^d^, ^f^	RR, 0.46 (0.35–0.61)	Study population	
				101 per 1000	55 fewer per 1000 (66 fewer to 41 fewer)
*The risk in the intervention group (and its 95% confidence interval) is based on the assumed risk in the comparison group and the **relative effect** of the intervention (and its 95% CI).					
**CI:** Confidence interval; **RR:** Risk ratio; **OR:** Odds ratio;					
**GRADE Working Group grades of evidence**					
**High certainty:** We are very confident that the true effect lies close to that of the estimate of the effect					
**Moderate certainty:** We are moderately confident in the effect estimate: The true effect is likely to be close to the estimate of the effect, but there is a possibility that it is substantially different					
**Low certainty:** Our confidence in the effect estimate is limited: The true effect may be substantially different from the estimate of the effect					
**Very low certainty:** We have very little confidence in the effect estimate: The true effect is likely to be substantially different from the estimate of effect					

^a^ Even though three (Dongol Singh et al., 2017; Fernández‐Carrocera et al., 2013; Kaban et al., 2019) of the included studies in the analysis had high ROB related to randomizations, the exclusion of these studies did not have much effect on the magnitude of the summary estimate or its statistical significance.

^b^ *I* ^2^ was 0%.

^c^ All‐cause mortality is an objective outcome and there were no concerns about the indirect measurement of the outcome.

^d^ The confidence interval of the summary estimate did not include 1.

^e^ *I* ^2^ was 23% and the p. value for heterogeneity was 0.16.

^f^ The *I* ^2^ was 24%.

###### Publication bias

**Figure 5:**
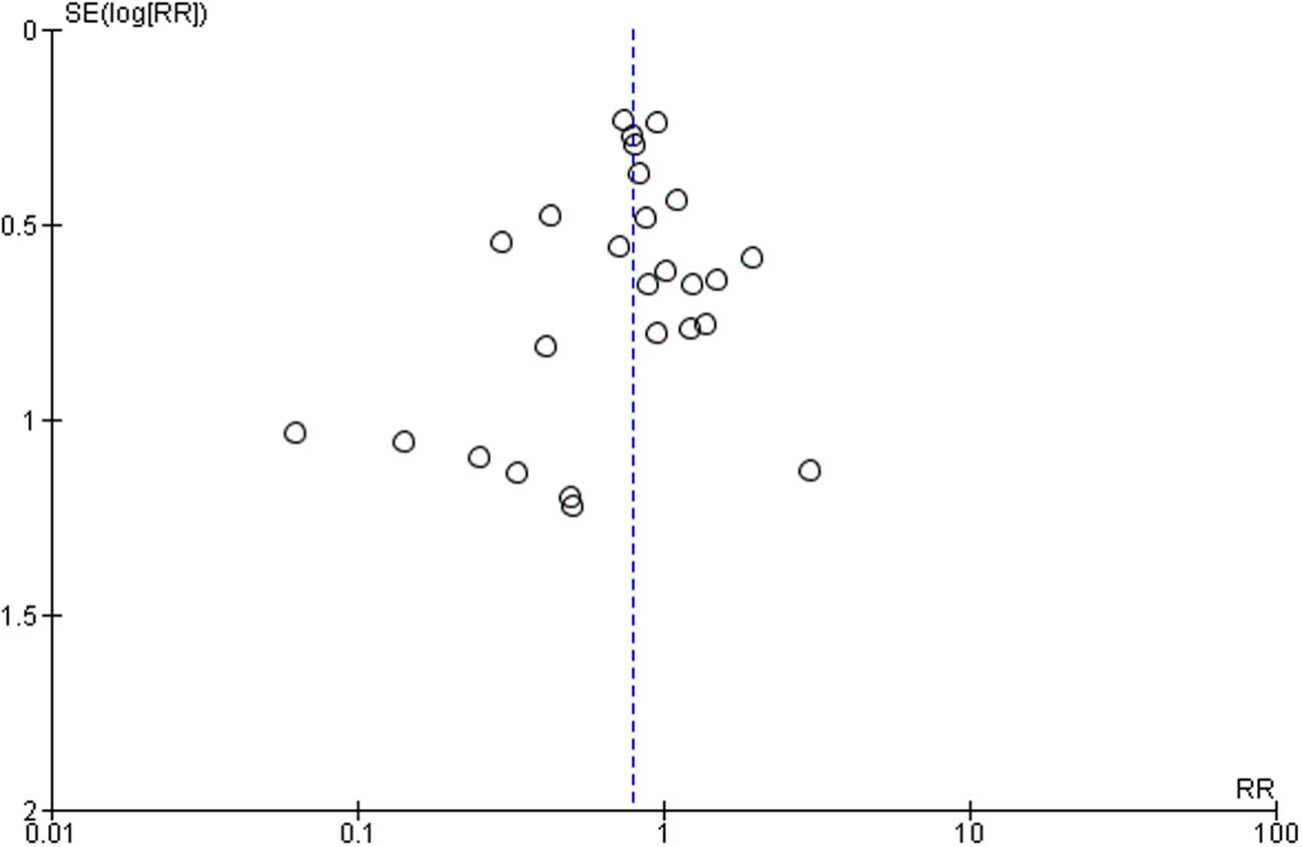
(Analysis 2.1) Funnel plot of comparison: 2 probiotics versus Control, outcome: 2.1 All‐cause mortality

A funnel plot for publication bias looked symmetrical (Figure 5).

###### Subgroup analyses

Table 1 gives the summary of data for subgroup analyses. For the outcome of all‐cause mortality, data were available to perform subgroup analyses based on settings (hospital vs. community‐based studies), type of probiotics (single strains vs. multiple strain vs. synbiotic), type of participants (term vs. preterm/low birth weight), type of feeding (breastfeeding vs. formula feeding vs. mixed feeding), and probiotic preparation (preparation containing *Lactobacillus* vs. *Bifidobacterium* vs. both *Lactobacillus* and *Bifidobacterium* vs. *S. boulardii*). No significant difference was noted among the subgroups; however, the number of studies varied for each group within the subgroup analysis.

###### Sensitivity analysis

####### Random versus fixed effect models

Use of a fixed‐effect model did not change the summary estimate for the effect of probiotics on all‐cause mortality (RR, 0.80; 95% CI, 0.66–0.96; heterogeneity: *χ* ^2^ = 22.55, (*p* = 0.55); *I* ^2^ = 0%) Analysis 2.7.

####### Risk of bias

Exclusion of three studies (Dongol Singh et al., 2017; Fernández‐Carrocera et al., 2013; Kaban et al., 2019) that were at high ROB for randomizations/allocation concealment did not change the summary estimate to a great extent, and results remained statistically significant Analysis 2.8.

##### Incidence of NEC

**Figure 6:**
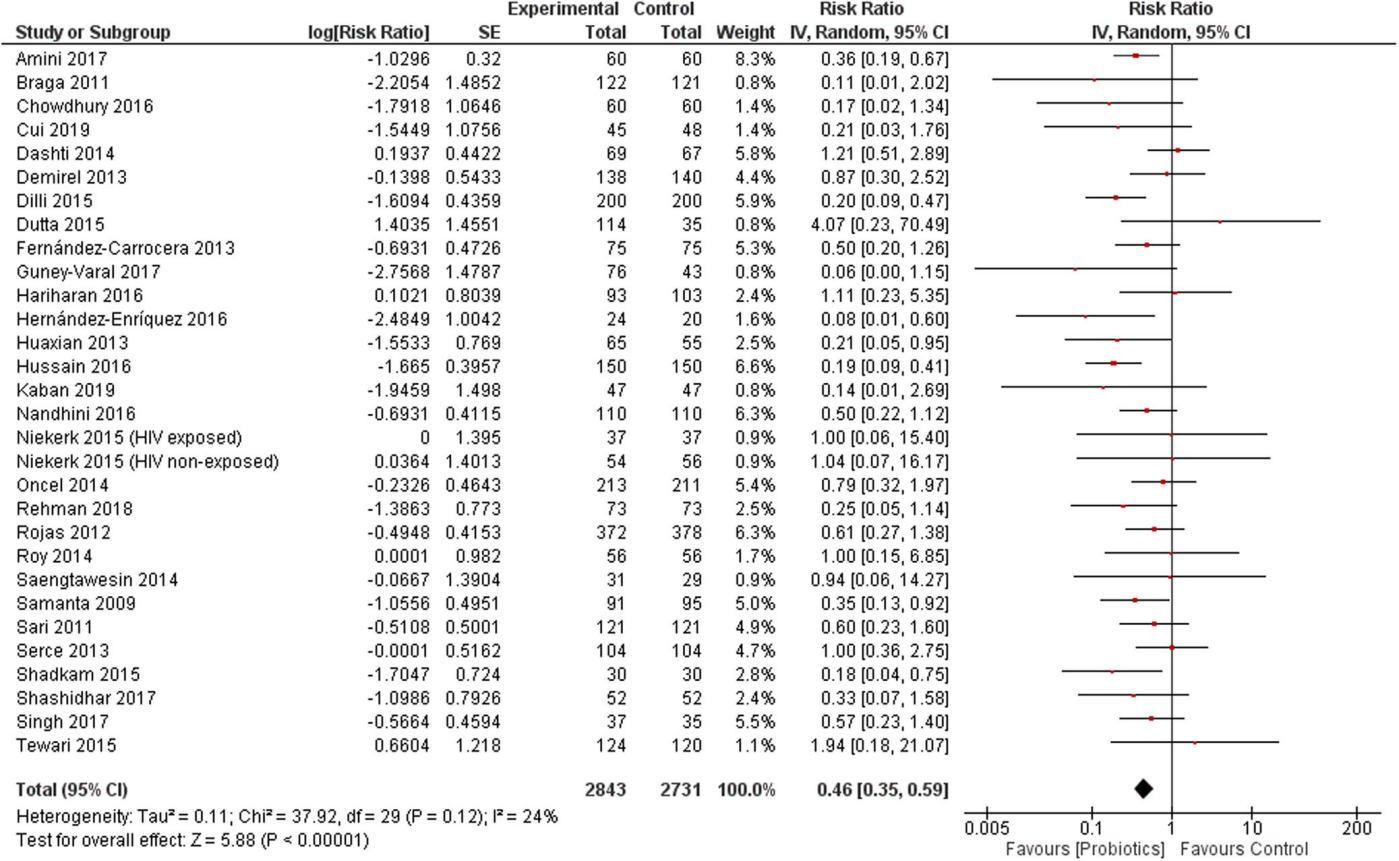
(Analysis 2.9) Forest plot of comparison: 2 probiotics versus control, outcome: 2.9 necrotizing enterocolitis (any type)

This outcome was reported by twenty‐nine studies that included a total of 5574 (probiotics, 2843; control, 2731) participants. The combined results showed that the probiotics group had a relative reduction in NEC prevalence of 54% compared to control group (RR, 0.46; 95% CI, 0.35–0.59; heterogeneity: *τ* ^2^ = 0.11; (*p* = 0.12); *I* ^2^ = 24%) (Figure 6). The number needed to treat was 17. We have high certainty in this evidence. Summary of findings Table 3.

###### Publication bias

A funnel plot for publication bias looked symmetrical.

###### Subgroup analyses

Table 1 shows the results of subgroup analyses. For NEC, data were available to perform the following 3 subgroup analyses: type of probiotics (single strain vs. multiple strain vs. synbiotic), type of feeding (breastfeeding vs. formula feeding vs. mixed feeding), and probiotic preparation (preparation containing *Lactobacillus* vs. *Bifidobacterium* vs. both *Lactobacillus* and *Bifidobacterium* vs. *S. boulardii*). No significant difference was noted among the subgroup analyses except the one based on probiotics preparation (*p* value for subgroup difference 0.05). The probiotics preparation that has *Lactobacillus* in it, either as a single strain (RR, 0.39; 95% CI, 0.25–0.61; 13 studies) or in combination with *Bifidobacterium* (RR, 0.44; 95% CI, 0.36–0.68; 13 studies), had a significant effect compared to Bifidobacterim alone (RR, 0.20; 95% CI, 0.09, −0.47) or *S. boulardii* alone (RR, 0.94; 95% CI, 0.45–1.95; two studies). This subgroup analysis should be interpreted carefully, as the number of studies was not uniformly distributed among all the subgroups, and the statistical difference might be due to the small number of studies in two of the subgroups.

###### Sensitivity analyses

####### Random versus fixed effect models

The use of a fixed effect model did not change the summary estimate for the effect of probiotics on NEC (RR, 0.45; 95% CI, 0.37–0.56; heterogeneity: *χ* ^2^ = 37.92, df = 29 (*p* = 0.12); *I* ^2^ = 24%).

####### Risk of bias

The exclusion of three studies (Dongol Singh et al., 2017; Fernández‐Carrocera et al., 2013; Kaban et al., 2019) with a high ROB due to randomization/allocation concealment did not change the summary estimate significantly.

##### Incidence of neonatal sepsis

The effect of probiotics on the incidence of neonatal sepsis was reported by 21 studies that included 9105 (probiotics, 4606; control, 4499) participants. The combined results showed a statistically significant reduction in incidence of sepsis of 22% in the intervention group compared to control (RR, 0.78; 95% CI, 0.70–0.86; heterogeneity: *τ* ^2^ = 0.01; (*p* = 0.16); *I* ^2^ = 23%). The number needed to treat was 14. The grade rating for this outcome was “high.” Summary of findings Table 3.

###### Publication bias

A funnel plot for publication bias looked symmetrical.

###### Subgroup analysis

Table 1 shows the results of subgroup analyses. Subgroup analysis could be done for the incidence of neonatal sepsis according to study setting, type of probiotic (single strain vs. multiple strain vs. synbiotic), type of feeding (breastfeeding vs. formula feeding vs. mixed feeding), and probiotic preparation (preparation containing *Lactobacillus* vs. *Bifidobacterium* vs. both *Lactobacillus* and *Bifidobacterium* vs. *S. boulardii*). Only the type of feeding differed among subgroups, probiotic supplementation seemed to have a significant effect for the prevention of neonatal sepsis when the supplementation was given to babies who received breastmilk only (RR, 0.76; 95% CI, 0.61–0.83; 8 studies) or breastmilk in combination with formula milk (RR, 0.76; 95% CI, 0.64–0.90; seven studies), *p* value for subgroups difference was 0.04.

###### Sensitivity analysis

####### Random versus fixed effect models

Use of a fixed effect model had a minimal effect on summary estimates for the effect of probiotics on the incidence of neonatal sepsis (RR, 0.79; 95% CI, 0.73–0.85; heterogeneity: *χ* ^2^ = 26.06, df = 20 (*p* = 0.16); *I* ^2^ = 23%).

####### Risk of bias

No significant difference was noted when two studies (Fernández‐Carrocera et al., 2013; Kaban et al., 2019) with a high ROB were excluded from the analysis Analysis 2.21.

##### Sepsis specific mortality

Two studies reported the data for sepsis specific mortality. The combined results showed a reduction of sepsis specific mortality of 89% in the intervention group compared to control (RR, 0.21; 95% CI, 0.04–1.01; heterogeneity: *τ* ^2^ = 0.00; *χ* ^2^ = 0.91, df = 1 (*p* = 0.34); *I* ^2^ = 0%). This is limited by a wide CI of the estimate including 1.

##### Adverse events

No adverse event was reported in any of the included studies.

##### Neurodevelopmental outcomes

Two studies assessed neurodevelopmental outcomes after neonatal probiotic supplementation. We did not perform a meta‐analysis for these outcomes, as these studies used different scales.

Sari et al. (2011) reported the data for neurodevelopmental outcomes at 18–22 months post neonatal probiotic supplementation for extremely low birth weight infants. Their results did not show any difference in growth and neurodevelopmental outcomes between the two groups. The growth outcomes were reported as weight (probiotic group 10.5 ± 1.7 kg vs. control 10.5 ± 1.7 kg, *p* value .92), length (probiotic group 79.4 ± 7.8 cm vs. control 81.0 ± 5.3 cm, *p* value .32), and head circumference (probiotic group 47.5 ± 6.5 cm vs. control 46.7 ± 1.8 cm, *p* value .53). The neurodevelopmental outcomes were reported as mental development index (probiotic group 90.7 ± 15.5 vs. control 90.4 ± 14.5, *p* value .88) and Psychomotor Development Index (probiotic group 95.4 ± 17.2 vs. control 93.2 ± 16.4, *p* value .39).

Oncel et al. (2014) also followed a cohort of extremely low birth weight infants after neonatal supplementation with probiotics or placebo. There was no significant difference in the neurodevelopmental outcomes at 18–24 months of age post supplementation, mental development index (probiotic 81 (median), 49 (min) to 124 (max) vs. placebo 82, 53–128; *p* value .48) or Psychomotor Development Index (probiotic 80, 49–112 vs. placebo 79, 49–107; *p* value .67).

## 6 DISCUSSION

### 6.1 Summary of main results

This review evaluated three neonatal nutritional interventions. Vitamin A supplementation during the neonatal period in the community setting did not have any significant effect on all‐cause mortality at 1, 6, or 12 months. We did not identify any studies from LMICs that assessed the use of dextrose gel for the treatment or prevention of neonatal hypoglycaemia. Probiotic supplementation during the neonatal period mainly given to low birth weight and preterm babies was shown to reduce all‐cause mortality, NEC, and neonatal sepsis.

### 6.2 Overall completeness and applicability of evidence

The evidence of neonatal vitamin A supplementation included 16 studies, and the number of participants in these studies exceeded 150,000. Overall, most of the included studies were at low ROB across many of the ROB items assessed using the Cochrane ROB scale. The statistical heterogeneity in the pooled data for mortality outcomes at 6 and 12 months was noted for neonatal vitamin A supplementation; however, the value of *I* ^2^ was <50%. Subgroup analyses done in a recent IPD analysis could potentially explain the reasons for heterogeneity and are discussed in Section 6.5.

The results for the use of probiotics during the neonatal period seem very promising. Almost all the studies in this meta‐analysis included preterm and/or low birth weight neonates. The effect of probiotics on all‐cause mortality was reported in 25 studies, and the analysis included more than 10,000 participants. The forest plot showed a homogenous effect in favor of the intervention with *I* ^2^ of 0%. We think that this effect is biologically plausible, and the most likely pathway of reduction in neonatal mortality from probiotic supplementation is via a reduction in sepsis and NEC, as shown in Analysis 2.15 and Analysis 2.9, respectively. We also think that these results are less likely due to bias. The studies by Guney‐Varal et al. (2017), Kaban et al. (2019), and Dongol Singh et al. (2017) were at high ROB due to inadequate randomization methods. The exclusion of these three studies from meta‐analysis for the effect of probiotics on all‐cause mortality did not change the summary estimate significantly (RR, 0.82; 95% CI, 0.68–0.99). A funnel plot for publication bias was symmetrical. The use of a fixed versus random effect model also did not change the results significantly. In addition to these observations, the fact that the effect of probiotics on the reduction of neonatal sepsis and NEC was mostly homogenous (*I* ^2^ of 23% and 24%, respectively) indicates that the use of probiotics could be beneficial for babies with low birth weight and preterm birth. We notice, however, that there was significant clinical heterogeneity in the dose, duration, and strains of probiotics used; this indicates that more research is needed to determine the appropriate dose and duration of probiotic supplementation in neonates.

The use of probiotics for the prevention of NEC, sepsis, and mortality in preterm and/or low birth weight babies has been debated in the past. The use of probiotics was advocated after the publication of a Cochrane review in 2011 (Alfaleh et al., 2011, later updated in 2014, AlFaleh & Anabrees, 2014) that showed that probiotics reduced NEC (stage II or more) (RR, 0.35; 95% CI, 0.24–0.52) and mortality (RR, 0.40; 95% CI, 0.27–0.60) in preterm/low birth weight neonates (Ofek Shlomai et al., 2014; Robinson, 2014). Others challenged the appropriateness of the meta‐analysis in the setting of clinical heterogeneity (Mihatsch et al., 2012; Mihatsch, 2011) and called for large trials before this intervention could be recommended in clinical practice (Mihatsch, 2011). This led to two large clinical trials, the ProPrems trial conducted in Australia and New Zealand (Jacob et al., 2013) and the PiPS trial conducted in the United Kingdom (Costeloe et al., 2016). The ProPrem trial used a mixture of probiotics (*Bifidobacterium infantis*, *Streptococcus thermophilus*, and *Bifidobacterium lactis*) and included 1099 preterm (<32 weeks) and very low birth weight (<1500 g) neonates. The results of the ProPrems trial showed that the use of probiotics did not reduce the incidence of sepsis and mortality, but did reduce the incidence of NEC (RR, 0.46; 95% CI, 0.23–0.93) (Jacob et al., 2013). The PiPS trial included 1315 neonates between the gestational age of 23–30 weeks who were randomised within 24–48 h to a single probiotic (*Bifidobacterium breve* BBG‐001) or placebo. The results of the PiPS trial showed no difference between the intervention and the control group for the outcomes of NEC (RR, 0.93; 95% CI, 0.68–1.27), sepsis (RR, 0.97; 95% CI, 0.73–1.29), or death (RR, 0.93; 95% CI, 0.67–1.30) (Costeloe et al., 2016). The results of these trials were surprising in the setting of known evidence from meta‐analyses of the available studies. A closer look at the results of the PiPS trial showed that there was significant contamination of the control group, as about 49% of the neonates from the control group had the same type of probiotic bacteria in their stool as those who were in the intervention group. This decreased the power of the study, and it was argued that a potential lack of effect might be explained by the cross‐contamination of the control group (Deshpande et al., 2016; McKinlay et al., 2016). The debate of appropriateness of probiotic supplementation in the neonatal period has continued, and a recent network meta‐analysis concluded that there is not enough evidence in favor of either a single or a mixture of strains of probiotics that could be suggested for routine clinical use for the prevention of NEC, sepsis, and/or mortality in preterm/low birth weight babies (van den Akker et al., 2018).

Our review focused on studies from LMIC. The effect of probiotic supplementation on mortality, sepsis, and NEC was significant when we pooled studies from these countries only. We noticed clinical heterogeneity in the use of probiotics in terms of type of probiotics, single versus multiple strains used, and baseline interventions such as the use of breastmilk. We think that the use of a meta‐analysis is appropriate to pool these studies, as the primary target of all the studies was the same, that is, correction of dysbiosis. We further demonstrated that subgroup analysis based on certain clinical factors reveals no significant difference in results. We notice, however, the relative lack of studies from the community setting. This is likely due to the increased rate of community over hospital‐based births in LMIC. For this reason, we read the results of a community‐based study by Panigrahi et al. (2017) with great interest. This study was the largest study conducted on the use of probiotics (synbiotics) and had a sample size of 4556; this is three times larger than the sample size of the PiPS trial. Panigrahi et al. (2017) used a synbiotic which was a mixture of the probiotic *Lactobacillus plantarum* ATCC‐202195 and the prebiotic fructooligosaccharide. This study recruited neonates who were at least 35 weeks of gestation and weighed at least 2000 g from rural settings from India. The results of the study showed a significant reduction in the primary outcome (combination of sepsis and death) in the intervention group compared to control (RR, 0.60; 95% CI, 0.48–0.74), culture‐positive and culture‐negative sepsis, and lower respiratory tract infections (Panigrahi et al., 2017). More such community‐based studies are needed from other countries examining term, preterm, and low birth weight infants.

Are probiotics safe for use during the neonatal period? We did not find any substantial evidence of adverse events with the use of probiotics in neonatal age group. It is important, however, to consider the safety considerations of probiotic supplementation in this vulnerable population. Probiotics are not regulated as a medication and are thus susceptible to variations in quality within and between countries. If not carefully produced and handled, probiotics may also contain pathogenic contaminants that may then lead to neonatal sepsis. Probiotic supplementation has been linked to both bacterial (Dani et al., 2016) as well as fungal sepsis (Vallabhaneni et al., 2014). Despite their rare occurrence, neonates receiving probiotics should be followed in a registry to ensure any reporting and observation of the potential risk of sepsis in a large sample size in the real world setting.

### 6.3 Quality of the evidence

The GRADE quality of evidence was considered high for most of the outcomes for neonatal vitamin A and probiotic supplementation. The GRADE method of assessment of overall evidence considers the type of study, ROB, statistical heterogeneity, indirectness, and imprecision of the summary estimates as well as the risk of publication bias (Guyatt et al., 2011).

All the included studies for neonatal vitamin A supplementation were randomized and had minimal ROB. The statistical heterogeneity for pooled studies for neonatal vitamin A supplementation was noticeable but was not significant enough to decrease our confidence in the summary estimate. Similarly, the CIs around the summary estimate were narrow, and there was no increased risk of publication bias for studies that assessed neonatal vitamin A supplementation.

All the included studies for neonatal probiotic supplementation were also randomized. High ROB was noted for three of the included studies (Dongol Singh et al., 2017; Fernández‐Carrocera et al., 2013; Kaban et al., 2019), but exclusion of these studies did not change the results of any of the outcomes, including all‐cause mortality, NEC, and neonatal sepsis. The pooled results were mostly homogenous, and the summary estimates were precise with narrow CIs. The funnel plots for publication bias were symmetrical for the outcomes of all‐cause mortality, NEC, and neonatal sepsis.

### 6.4 Potential biases in the review process

We used standard methods of Campbell and Cochrane collaborations to conduct the review. Two review authors screened the titles and abstracted the data from the included studies. Our inclusion/exclusion criteria were decided a priori, and a peer reviewed protocol was published giving details of methods of conduct of this review.

We performed two posthoc subgroup analyses for the effect of probiotic supplementation during the neonatal period. This analysis was based on the type of feeding, as our team thought it was essential to establish any differential effect of probiotics when the intervention was delivered with breastmilk or formula or both. The results for this analysis were similar among the subgroups for outcomes of all‐cause mortality and NEC; however, there was significant heterogeneity among subgroups for the outcome of neonatal sepsis Analysis 2.18. A close examination of the data showed that the difference among subgroups was due to the group where the status of the feeding was “unclear.” Exclusion of this subgroup showed a homogenous protective effect in the case of breastmilk or formula milk‐fed babies against neonatal sepsis (data not shown). So, we think that probiotics may have a significant protective effect against neonatal sepsis, NEC, and mortality irrespective of the type of food offered. The second posthoc subgroup analysis was based on study setting. We were interested in knowing if the probiotics had a similar effect on infants born in the hospital setting compared to those in the community setting. We were interested in this analysis because a significant number of births happen at home in LMIC. There were a limited number of studies conducted in the community setting that addressed the effect of probiotics; therefore, no solid conclusion could be drawn at this time for any of the outcomes.

### 6.5 Agreements and disagreements with other studies or reviews

The effects of neonatal vitamin A supplementation have been reviewed in two Cochrane reviews (Darlow et al., 2016; Haider et al., 2017). The Cochrane review by Haider et al. (2017) focused on randomized studies from the community setting only. We considered studies from both community and hospital settings. We updated the literature search and found one additional study from the community setting (Ahmad et al., 2019) and added three studies from the hospital setting (Basu et al., 2019; Giridhar et al., 2019; Sun et al., 2019). The studies from hospital settings were done in the neonatal intensive care setting for very low birth weight babies. We did not pool the results of these studies with those of the community‐based studies. The Cochrane review on neonatal vitamin A supplementation for very low birth weight infants included 11 trials and reported a reduction in risk of death or oxygen requirement at 1 month of age (RR, 0.93; 95% CI, 0.88–0.99). Of the three studies we included in our review from LMIC, only one study showed a reduction in composite outcome of incidence of all‐cause mortality and oxygen requirement for 28 days (RR, 0.44; 95% CI, 0.229–0.844)] from vitamin A supplementation in very low birth weight infants (Basu et al., 2019). The new study for neonatal vitamin A supplementation from the community was small and included 306 participants. The addition of this study did not change the results significantly compared to those published in the 2017 Cochrane review (Haider et al., 2017).

An individual participant meta‐analysis of neonatal vitamin A studies conducted in the community setting was published during the preparation of this review (West et al., 2019). This review addressed multiple subgroup analyses both at the study‐ and individual‐level characteristics and used the original data from individual trials to pool the studies. The overall results were similar to our results for all‐cause mortality at 6 and 12 months (West et al., 2019). The subgroup analysis based on study‐level characteristics showed that neonatal vitamin A supplementation significantly reduced 6‐month mortality among the trials conducted in South Asia (RR, 0.87; 95% CI, 0.77–0.98) but not in Africa; they also showed a potential for increased risk of mortality in African countries (RR, 1.07; 95% CI, 1.00–1.15). Further subgroup analyses showed that neonatal vitamin A supplementation reduced all‐cause mortality in the context of moderate or severe maternal VAD (defined as 10% or higher proportion of women with serum retinol <0.7 μmol/L or 5% or more women with night blindness) (RR, 0.87; 95% CI, 0.80–0.94), in settings where baseline (control group) early infant mortality was 30 or more per 1000 live births (RR, 0.91; 95% CI, 0.85–0.98), and in the context of lack of maternal education (>32% mothers had no schooling) (RR, 0.88; 95% CI, 0.80–0.96). The subgroup analyses conducted based on individual‐level characteristics such as sex, birth weight, gestational age and size, age at dosing, parity, time of breastfeeding initiation, maternal education, and maternal vitamin A supplementation did not show any significant differential effect of neonatal vitamin A supplementation compared to placebo for these groups. As most of the subgroup analyses that we prespecified in our review were addressed in this study (West et al., 2019), we did not repeat these analysis in our current study.

Oral dextrose as a treatment of hypoglycaemia and prevention of hypoglycaemia in high‐risk neonates has been evaluated in two Cochrane reviews (Hegarty et al., 2017; Weston et al., 2016). The review by Weston et al. (2016) addressed treatment of neonatal hypoglycaemia and included two studies, one from New Zealand and another from Ireland. They did not show any major difference in episodes of hypoglycaemia between the two study groups. The review by Hegarty et al. (2017) addressed the prevention of hypoglycaemia in high‐risk neonates and included one study from New Zealand. The included study showed a significant reduction in hypoglycaemia episodes in the intervention group compared to control (RR, 0.76; 95% CI, 0.62–0.94). No randomized study was available from LMIC in either of the two reviews mentioned above. We updated the searches and did not find any study from LMIC.

Other reviews have been published that assess the effect of probiotic supplementation during the neonatal period. A Cochrane review was published in 2011 (Alfaleh et al., 2011) with an update in 2014 (AlFaleh & Anabrees, 2014). This has not since been updated. The Cochrane review included studies from LMIC and high‐income countries and concluded that probiotic supplementation reduced NEC (stage II or more) (RR, 0.43; 95% CI, 0.33–0.56) and mortality (RR, 0.65; 95% CI, 0.52–0.81) but showed no effect for nosocomial sepsis (RR, 0.91; 95% CI, 0.80–1.03). More studies have been published since the publication of the 2014 Cochrane review update. Deshpande et al. (2017) reviewed studies from LMIC that addressed probiotic supplementation during the neonatal period. This review included twenty‐three studies and concluded that probiotic supplementation reduced all‐cause mortality (RR, 0.73; 95% CI, 0.59–0.90), NEC (RR, 0.46, 95% CI, 0.34–0.61), and neonatal sepsis (RR, 0.80; 95% CI, 0.71–0.91). We included thirty‐three studies and updated the meta‐analyses. With this new data, the magnitude and the statistical significance remained the same, but the summary estimates became more precise for the outcomes of all‐cause mortality, NEC, and sepsis. We also conducted additional subgroup analyses that were not previously performed.

## 7 AUTHORS' CONCLUSIONS

### 7.1 Implications for practice

Neonatal vitamin A supplementation in the community setting does not appear to reduce infant mortality at 1, 6, or 12 months of age. Vitamin A supplementation during neonatal period increases the risk of bulging fontanelle. No data were available for dextrose gel supplementation for the prevention or treatment of neonatal hypoglycaemia in LMIC.

Probiotic supplementation is a promising intervention and can reduce all‐cause mortality, neonatal sepsis, and NEC in low birth weight and/or preterm babies in LMIC in the hospital setting. Though we observed no adverse effects, infants receiving probiotics should be entered into a registry in order to observe any concerns for safety in large sample sizes in the real world setting.

### 7.2 Implications for research

There was significant clinical heterogeneity in terms of strains and dose of probiotics used in the included studies. More studies are needed to decide upon the right strain and optimal dose and duration of probiotics supplementation. Most of the included studies for probiotics supplementation were conducted in preterm/low birth weight babies in the neonatal intensive care unit. It is not clear if the similar protective effects would be seen when probiotics are given in the community setting or to term babies. It is also unclear if the supplementation of prebiotics and probiotics together (synbiotics) is more effective than probiotics alone. More studies are needed to answer these questions and to assess the effect of dextrose supplementation for the prevention and treatment of neonatal hypoglycaemia in LMIC.

## CONTRIBUTIONS OF AUTHORS

Aamer Imdad, Deepika Ranjit wrote the first draft of the protocol. Gamael S. S. Surin participated in the design of the search strategy and writing of the protocol. Abigail Smith and Sarah Lawler designed the search strategy. Aamer Imdad wrote the manuscript for the final review. Aamer Imdad, Faseeha Rehman, Evans Davis, Gamael S. S. Surin, Deepika Ranjit, and Suzanna L. Attia helped with data extraction and analysis. Suzanna L. Attia and Aamer Imdad edited the manuscript. Zulfiqar A. Bhutta supervised and gave feedback for the design of the protocol and the main review.

## DECLARATIONS OF INTEREST

Zulfiqar A. Bhutta was principle investigator of study (Soofi et al., 2017). He was not involved in the selection of the study for this review and also did not participate in the data extraction from this study. All other authors declare that they do not have any conflict of interest.

## DIFFERENCES BETWEEN PROTOCOL AND REVIEW

– The planned subgroup analyses for vitamin A were not conducted, as the same analyses were available from a recent IPD analysis (West et al., 2019).
– We did not use EPOC methodology for the ROB assessment, as all the included studies were RCTs and the Cochrane ROB assessment tool was used for the same.
– We did two posthoc subgroup analyses for probiotic supplementation during the neonatal period. These included type of feeding and study setting.
– We did the sensitivity analysis for ROB based on sequence generation and allocation concealment.

## PUBLISHED NOTES

### CHARACTERISTICS OF STUDIES

#### CHARACTERISTICS OF INCLUDED STUDIES

Ahmad et al. (2019)

**Table:** 

**Methods**	A block‐randomized, double‐masked, placebo‐controlled intervention trial conducted in Bangladesh
**Participants**	**Inclusion criteria**: "consent of the mother and willingness to have their infant participate; singleton birth at MCHTI clinic and eligible for vaccination according to the national and MCHTI clinic policy".
	**Exclusion criteria**: "planned at home delivery because of the low likelihood of vaccination at MCHTI within 48 h of birth, (2) congenital disease or a serious infection showing that the infant was not healthy; infant with birth weight <1500 g and inability to enrol within 48 h of birth due to lack of timely notification or other exceptional circumstances".
**Interventions**	**Intervention group**: 50,000 IU vitamin A (retinyl palmitate)
	**Comparison**: Placebo (unfortified soya based oil)
	The intervention was delivered within 48 h of birth
**Outcomes**	Neonatal mortality, adverse events, microbiome changes, thymus size
**Notes**	Data on mortality and bulging fontanelle were taken from publication (J Nutr 2019;00:1–8). Data on mortality was reported at 15 weeks. We included the data with “all‐cause mortality at 6 months".

Risk of bias table

**Table:** 

Bias	Authors' judgement	Support for judgement
Random sequence generation (selection bias)	Low risk	Quote: "The randomization lists of vitamin A and placebo within each group were generated by WHO, using Stata, v11"
Allocation concealment (selection bias)	Low risk	Quote: "Preplanned statistical analyses using arbitrary group identifiers…"
		Comment: Most likely done
Blinding of participants and personnel (performance bias)	Low risk	Quote: "dose of VA in oil or an identical placebo (PL) within 48…"
		Comment: Most likely done
Blinding of outcome assessment (detection bias)	Low risk	Quote: "Preplanned statistical analyses using arbitrary group identifiers (group 1, group 2) were completed on 4 April 2014 before unblinding…"
Incomplete outcome data (attrition bias)	Low risk	Attrition rate: 5.2%
Selective reporting (reporting bias)	Low risk	Author prespecified the outcomes. Trial was registered as ClinicalTrials.gov: NCT01583972.
Other bias	Low risk	No other risk of bias was noted

Amini et al. (2017)

**Table:** 

**Methods**	Prospective randomized control trial conducted in Iran
**Participants**	**Inclusion Criteria**: "All premature newborns (*n* = 115) weighting 750–1500 g or <32 weeks' gestation who received antibiotics and total parenteral nutrition in NICU of Vali Asr Hospital were included"
	**Exclusion Criteria**: "Premature babies <750 and more than 1500 g and neonates with congenital heart disease, congenital malformations, and immune system deficiency, even in their family members, were excluded from the study."
**Interventions**	**Intervention:** Multistrain powder probiotic infant formula containing *Streptococcus thermophilus*, *Lactobacillus rhamnosus*, *Lactobacillus acidophilus*, *Lactobacillus bulgaricus*, *Bifidobacterium infantis*, *Lactobacillus casei*. The dose was 0.8–1 g per day in 8–10 doses given for 13 days
	**Comparison:** Enteral feed without probiotic
**Outcomes**	NEC
**Notes**	Data were taken from table 3

Risk of bias table

**Table:** 

Bias	Authors' judgement	Support for judgement
Random sequence generation (selection bias)	Low risk	Quote: "In this double blind randomized clinical trial (RCT), block randomization was used and 60 cases were randomly divided into 2 groups."
Allocation concealment (selection bias)	Unclear risk	No clear information was available about allocation concealment
Blinding of participants and personnel (performance bias)	Unclear risk	No clear information available
Blinding of outcome assessment (detection bias)	Unclear risk	No clear information available
Incomplete outcome data (attrition bias)	Low risk	Minimal loss to follow up
Selective reporting (reporting bias)	Low risk	Authors seem to report all the outcomes irrespective of their statistical significance
Other bias	Low risk	No other risk of bias was noted

Basu et al. (2019)

**Table:** 

**Methods**	A randomized double‐blind placebo‐controlled trial India
**Participants**	**Inclusion Criteria:** Inborn, VLBW (birth weight (BW) < 1500 g) neonates admitted in NICU and requiring respiratory support in the form of oxygen inhalation through nasal prongs or head box, continuous positive airway pressure (CPAP), high flow nasal cannula (HFNC), or mechanical ventilation (MV) at the age of 24 h, were included.
	**Exclusion Criteria:** Neonates with major congenital malformation, any life‐threatening condition such as reversal of umbilical artery end‐diastolic blood flow on antenatal Doppler, perinatal asphyxia with moderate to severe hypoxic ischemic encephalopathy, shock with escalating doses of vasopressors, recurrent seizures, and suspected inborn errors of metabolism
**Interventions**	**Intervention:** 10,000 IU of retinol/dose, alternate days, 28 days or until discharge
	**Compairson:** Placebo
**Outcomes**	All‐cause mortality, sepsis, NEC
**Notes**	Study conducted in very low birth weight babies

Risk of bias table

**Table:** 

Bias	Authors' judgement	Support for judgement
Random sequence generation (selection bias)	Low risk	Quote: "Randomization into vitamin A or placebo group was done using random permuted blocks of 4, 6, and 8, prepared by an independent statistician not involved in the study."
Allocation concealment (selection bias)	Low risk	Quote: "Allocation into vitamin A or placebo group was done using serially numbered opaque and sealed envelopes by on‐duty residents who were appropriately trained for the process beforehand. Allocation concealment was maintained throughout the study."
Blinding of participants and personnel (performance bias)	Low risk	Quote: "Vitamin A and placebo oral solutions were supplied in identical bottles of 20 mL with dropper marked at 1 mL…."
Blinding of outcome assessment (detection bias)	Low risk	Quote: "Treating physicians, nursing staffs, and the parents were unaware about the composition of the bottles."
Incomplete outcome data (attrition bias)	Low risk	Three patients from the intervention and two patients from the placebo group left against medical advice
Selective reporting (reporting bias)	Low risk	Authors seem to report all the relevant outcomes
Other bias	Low risk	No other risk of bias was noted

Benn et al. (2008)

**Table:** 

**Methods**	Randomized placebo controlled trial conducted in Guinea‐Bissau
**Participants**	**Inclusion Criteria**: Weight at least 2500 g at presentation and no signs of overt illness or malformations
	**Exclusion Criteria**: Weight <2500 g at presentation and/or signs of overt illness and/or malformations. Also, infants who died in the maternity ward before the vaccination team could arrive
	Total number randomized to the intervention group: 2145
	Total number randomized to the control group: 2200
**Interventions**	Intervention: 50,000 IU vitamin A intradermally
	Control: 0.5 ml vegetable oil intradermally
	Common intervention given to all groups: 10 IU vitamin E intradermally
**Outcomes**	Primary outcomes: Overall mortality
	Other outcomes: Bulging fontanelles, vomiting, irritability, infections, fever, skin problems, and healthcare contacts
**Notes**	Vitamin A supplementation appeared to benefit boys but was harmful to girls

Risk of bias table

**Table:** 

Bias	Authors' judgement	Support for judgement
Random sequence generation (selection bias)	Low risk	Quote: "The mother drew a lot from an envelope prepared by the study supervisor. Each envelope contained 100 lots—50 marked “1” and 50 marked “2”—indicating from which of two numbered bottles, “1” or “2,” the child should receive the supplement"
		Comment: Most likely done
Allocation concealment (selection bias)	Low risk	Quote: "The lots were folded, making it impossible to tell what was written on them before they were opened"
		Comment: Most likely done
Blinding of participants and personnel (performance bias)	Low risk	Quote: "When asked, none of the three assistants who were responsible for the randomisation procedures at the hospital and at the heath centres had any idea which bottles contained vitamin A and which placebo. We concluded that the blinding of mothers and assistants was successful"
		Comment: Most likely done
Blinding of outcome assessment (detection bias)	Unclear risk	Quote: "Accumulating evidence for sex differential effects of vitamin A supplementation during the trial made us hypothesise before we started the analyses that supplementation would be particularly beneficial for boys"
		Comment: Most likely done
Incomplete outcome data (attrition bias)	Low risk	Total number of loss to follow up: 70 (1.6%)
		The loss to follow up was not balanced
Selective reporting (reporting bias)	Low risk	Authors seem to report all the outcomes irrespective of their statistical significance
Other bias	Low risk	No other risk of bias was noted

Benn et al. (2010)

**Table:** 

**Methods**	Randomized placebo controlled two by two factorial trial conducted in Guinea‐Bissau
**Participants**	Inclusion criteria: Weight <2500 g at presentation
	Exclusion criteria: Weight >2500 g at presentation
	Total number randomized to the intervention group: 864
	Total number randomized to the control group: 872
**Interventions**	Intervention: 25,000 IU vitamin A intradermally
	Control: 0.5 ml vegetable oil intradermally
	Common intervention given to all groups: 10 IU vitamin E intradermally
**Outcomes**	Primary outcomes: Infant mortality
	Other outcomes: Fever, septicaemia, malaria, malnutrition, and respiratory infections
**Notes**	

Risk of bias table

**Table:** 

Bias	Authors' judgement	Support for judgement
Random sequence generation (selection bias)	Low risk	Quote: "Once consent was provided, the mother drew an envelope from a bag. Each bag was prepared by the study supervisor and contained 48 envelopes; each envelope contained a lot name. Within each bag were 12 envelopes with lots marked “BCG 6,” 12 marked “BCG 7,” 12 marked “no BCG 6,” and 12 marked “no BCG 7.” The numbers “6” and “7” indicated from which of two numbered bottles, “6” or “7,” the child should receive treatment (that is, either 25,000 IU vitamin A or placebo)"
		Comment: Most likely done
Allocation concealment (selection bias)	Low risk	Quote: "The envelopes were closed and non‐transparent, making it impossible to identify the allocation before the envelopes were opened"
		Comment: Most likely done
Blinding of participants and personnel (performance bias)	Unclear risk	Quote: "Once consent was provided, the mother drew an envelope from a bag"
		"Each bag was prepared by the study supervisor and contained 48 envelopes; each envelope contained a lot name"
		Comment: Most likely done
Blinding of outcome assessment (detection bias)	Low risk	Quote: "Follow‐up was performed by assistants who were unaware of the allocated treatment"
		Comment: Most likely done
Incomplete outcome data (attrition bias)	Unclear risk	Total number of loss to follow up: 145 (8.4%)
		The loss to follow up was not balanced
Selective reporting (reporting bias)	Low risk	Authors seem to report all the outcomes irrespective of their statistical significance
Other bias	Low risk	No other risk of bias was noted

Benn et al. (2014)

**Table:** 

**Methods**	Double‐blind, placebo‐controlled randomized trial conducted in Guinea‐Bissau
**Participants**	**Inclusion criteria**: Normal birth‐weight neonates who were healthy and due for BCG vaccination
	**Exclusion criteria**: Birth weight <2500 g at presentation or overt illness and/or malformations
	Total number randomized to the intervention group 1 (50,000 IU vitamin A): 2015
	Total number randomized to the intervention group 25 (25,000 IU vitamin A): 2011
	Total number randomized to the control group: 2022
**Interventions**	Intervention: 50,000 IU vitamin A intradermally or 25,000 IU vitamin A intradermally
	Control: 0.5 ml Vegetable oil intradermally
	Common intervention given to all groups: 10 IU vitamin E intradermally
**Outcomes**	Primary outcome: Infant mortality
	Other outcomes: None measured
**Notes**	

Risk of bias table

**Table:** 

Bias	Authors' judgement	Support for judgement
Random sequence generation (selection bias)	Low risk	Quote. "Each envelope was prepared by the data manager, who did not take part in the enrolment procedures, and contained 48 folded lots indicating from which of 3 numbered bottles—“3,” “4,” or “5”—the child should receive his or her supplement"
		Comment: Most likely done
Allocation concealment (selection bias)	Low risk	Quote. "…48 folded lots indicating from which of 3 numbered bottles—“3,” “4,” or “5”—the child should receive his or her supplement"
		Comment: Most likely done
Blinding of participants and personnel (performance bias)	Low risk	Quote. "At each inclusion site, the randomization procedure was carried out by 1 carefully trained assistant every day except during short vacations. After providing consent, the mother drew a lot from an envelope. Each envelope was prepared by the data manager, who did not take part in the enrolment procedures…"
		Comment: Most likely done
Blinding of outcome assessment (detection bias)	Low risk	Quote. "The registration system assistants and the special team were unaware of the allocated treatment, because they were not present during enrolment, and the information was not transferred to the children's vaccination card or follow‐up forms"
		Comment: Most likely done
Incomplete outcome data (attrition bias)	Unclear risk	Quote: “Of 6053 children invited to participate, 6048 were randomly allocated to each of the 3 groups (50,000 IU vitamin A, 25,000 IU vitamin A, or placebo) (Figure 1). The 3 randomly assigned groups were similar in terms of their background characteristics (Table 1). A total of 176 deaths occurred; 2 of these were due to accidents and were censored. Fourteen deaths occurred after the child had been eligible for a national vitamin A campaign. Hence, censoring for accidents and subsequent VAS, the cohort had 160 deaths during 4125 person‐years of risk, corresponding to an MR of 39 per 1000 person‐years”
Selective reporting (reporting bias)	Low risk	Authors seem to report all the outcomes irrespective of their statistical significance
Other bias	Low risk	No other risk of bias was noted

Braga et al. (2011)

**Table:** 

**Methods**	Randomized, double blind control study conducted in Brazil
**Participants**	**Inclusion criteria:** All infants included in this study were born locally and admitted to the Neonatal Intensive Care Unit (NICU) with a birth weight from 750 to 1499 g, and had no major congenital malformations, life threatening chromosomal alterations, or congenital infections
**Interventions**	**Intervention:** Probiotic supplementation: *Bifidobacterium breve* and *Lactobacillus casei*: The intervention was started on the second day of life and was maintained until 30 d of life, a diagnosis of NEC, discharge from the hospital, or death, whichever occurred first. The dose was 3 ml human milk from the bank milk to which L. casei and B. breve had been added providing 3.5 × 10^7^ to 3.5 × 10^9^ CFU
	**Comparison**: The control group received the same volume of human milk without probiotics
**Outcomes**	Mortality, NEC and sepsis
**Notes**	Authors did not do intention to treat analysis. We created the intention to treat analysis by taking the number randomized as denominators. The data on outcomes was taken from table 2 of the main manuscript

Risk of bias table

**Table:** 

Bias	Authors' judgement	Support for judgement
Random sequence generation (selection bias)	Low risk	Randomization was carried out in blocks of 10, and the list of random numbers was generated by the subprogram Epitable from Epi‐Info 6.04
Allocation concealment (selection bias)	Low risk	A sealed envelope with the identification number in ascending order, containing information about which group they belonged to, was provided for each infant and sent to the hospital's nutritional centre
Blinding of participants and personnel (performance bias)	Low risk	Neither the medical and nursing staff responsible for monitoring the infants nor the researchers were aware of which group the infants were allocated to
Blinding of outcome assessment (detection bias)	Low risk	Neither the medical and nursing staff responsible for monitoring the infants nor the researchers were aware of which group the infants were allocated to
Incomplete outcome data (attrition bias)	Low risk	Attrition in intervention group was 2% and 7% in the control group
Selective reporting (reporting bias)	Low risk	Most of the outcomes were reported
Other bias	Low risk	No other bias was noted

Chowdhury et al. (2016)

**Table:** 

**Methods**	A randomized controlled trial conducted in Bangladesh
**Participants**	**Inclusion crietria**: Preterm (<33 woks), VLBW (<1500 g) infants who are able to tolerate oral feeds and survive beyond 48 h
	**Exclusion criteria**: Babies with suspicion of clinical sepsis, presence of prenatal asphyxia, major congenital anomaly and babies who expired due to other neonatal illness were excluded
**Interventions**	**Intervention:** Probiotic supplementation: *Bifidobacterium breve* and *Lactobacillus casei*: The dose was 3 ml once daily of solution containing *Bifidobacterium breve* and *Lactobacillus casei* 10^6^ CFU. The intervention was continued for at least 10 days
	**Comparison:** No probiotics
**Outcomes**	NEC, all‐cause mortality
**Notes**	Authors did not perform intention to treat analysis, however, we created the intention to treat analysis from Figure 1. The data for mortality was taken from Figure 1 and the data for NEC was taken from table 2

Risk of bias table

**Table:** 

Bias	Authors' judgement	Support for judgement
Random sequence generation (selection bias)	Low risk	Coding to group 1 and 2 was done by a faculty of another department not related to this study. First case was selected to 1 group by lottery method and subsequent group was continued accordingly
Allocation concealment (selection bias)	Low risk	Participants and investigators did not know group allocation
Blinding of participants and personnel (performance bias)	Low risk	Probiotics were added to breast milk by registrar or assistant registrar of the corresponding unit before feeding
Blinding of outcome assessment (detection bias)	Low risk	Participants and investigators did not know group allocation
Incomplete outcome data (attrition bias)	Low risk	15% attrition. Reasons for loss to follow up reported.
Selective reporting (reporting bias)	Low risk	All expected outcomes were reported
Other bias	Low risk	No concerns for other risk of bias

Cooper et al. (2017)

**Table:** 

**Methods**	A randomized double‐blind controlled trial conducted in South Africa in community settings
**Participants**	**Inclusion criteria**: "healthy", full term (37–42 weeks), born to HIV + formula feeding mothers, ≤3 days old, 2500–4500 g, singleton birth
	**Exclusion criteria**: Congenital illness or malformation affecting growth; significant perinatal disease, antibiotics in 1st 3 days of life, caregivers could not comply, or in another trial
**Interventions**	**Intervention**: Probiotics: Formula containing prebiotic (bovine milk‐derived oligosaccharides) and probiotic ((B. lactis strain CNCM‐I‐3446 with 1 × 10^7^ cfu/g. of powder formula). The duration of intervention was 6 months
	**Comparison**: Formula without prebiotic and probiotic
**Outcomes**	All‐cause mortality
**Notes**	The data was taken from the last paragraph of the result section. We note that the intervention group received both prebiotics and probiotics and the control group did not receive any prebiotics or probiotics. Some of the study participants were HIV positive

Risk of bias table

**Table:** 

Bias	Authors' judgement	Support for judgement
Random sequence generation (selection bias)	Low risk	Quote: "The randomization was performed using the in‐house TrialSys software".
Allocation concealment (selection bias)	Low risk	Formulas labelled similarly
Blinding of participants and personnel (performance bias)	Low risk	Parents (caregivers), investigators, study support staff, and the clinical project managers were blinded to the identity of the products.
Blinding of outcome assessment (detection bias)	Low risk	Likely same care‐team
Incomplete outcome data (attrition bias)	Low risk	Total loss to follow up was 1%
Selective reporting (reporting bias)	Low risk	Authors do not seem to selectively report outcomes
Other bias	Low risk	No other risk of bias was noted.

Cui et al. (2019)

**Table:** 

**Methods**	A prospective, double‐blinded randomized study conducted in China
**Participants**	**Inclusion criteria**: Formula‐fed preterm infants, gestational age ≥30 and <37 weeks; birthweight ≥1500 g and ≤ 2000 g with vital sign and hemodynamic parameters stable
	**Exclusion criteria**: Congenital diseases, expected hospitalisations <2 weeks and maternal or neonatal antibiotics or other probiotics before admission
**Interventions**	**Intervention**: *Lactobacillus reuteri* DSM 17938, five drops daily for minim of 7 days. Each drop had 1×10^8^ colony‐forming units
	**Comparison**: Placebo
**Outcomes**	Sepsis, NEC, growth
**Notes**	Data were taken from table 2

Risk of bias table

**Table:** 

Bias	Authors' judgement	Support for judgement
Random sequence generation (selection bias)	Low risk	Quote: "Randomization was conducted according to a random computer‐determined allocation order considering gestational age"
		Comment: Most likely done
Allocation concealment (selection bias)	Unclear risk	No clear information available for allocation concealment
Blinding of participants and personnel (performance bias)	Low risk	Quote: "Blinding was possible because the nurses who administered L. reuteri to the infants were not involved in the daily care and the attending neonatal team was unaware of the randomization assignments"
Blinding of outcome assessment (detection bias)	Unclear risk	It was not clear if the families of the participating neonates were aware of the treatment assignments
Incomplete outcome data (attrition bias)	Low risk	About 18% attrition reported that was balanced in two groups
Selective reporting (reporting bias)	Low risk	Authors seem to report all the relevant outcomes
Other bias	Low risk	No other risk of bias was noted

Dashti et al. (2014)

**Table:** 

**Methods**	Prospective triple‐blinded, interventional, randomized clinical trial
**Participants**	**Inclusion criteria**: Birth weight of 700‐1800 g, stable hemodynamic, be able to have enteral feeding, and written parental consent.
	**Exclusion criteria**: Evidence or suspicion of congenital intestinal obstruction or perforation, prenatal or postnatal diagnosis of gastroschisis, large omphalocele, or congenital diaphragmatic hernia, and major congenital anomalies."
**Interventions**	**Intervention**: Protexin (probiotics). Protexin (Restore): 1 × 109 CFU (colony forming unit), 1 g (one sachet) contains: *Lactobacillus acidophilus*, *Lactobacillus rhamnosus*, *Bifidobacterium longum*, *Lactobacillus bulgaricus*, *Lactobacillus casei*, *Streptococcus thermophilus*, *Bifidobacterium breve*, and *Bifidobacterium*
	The dose was as follows
	– Neonates weighing <1000 g were fed with a half of sachet once daily (5×10^8^ CFU of probiotics), – Neonates weighing 1001–1500 g were fed with 3/4 of a sachet once daily (7.5×10^8^ CFU of probiotics) – Neonates weighing more than 1500 g were fed with a full sachet once daily (1×10^9^ CFU of probiotics).
	**Control**: "placebo that was physically indistinguishable from the probiotic powder"
**Outcomes**	NEC, mortality and sepsis
**Notes**	The data for NEC, mortality and sepsis was taken from table 2. We assumed that group A was the intervention group and group B was the control. Authors did not mention clearly in the paper which group is the intervention group and which one is the control group.The duration of intervention was not clearly stated

Risk of bias table

**Table:** 

Bias	Authors' judgement	Support for judgement
Random sequence generation (selection bias)	Low risk	No clear data is available to support the assessment however the two groups were comparable after randomizations and allocation seems to be concealed. So less likely that randomizations was not done properly
Allocation concealment (selection bias)	Low risk	Quote: "To blind the trial the probiotic and placebo sachets were set in similar indistinguishable packages"
Blinding of participants and personnel (performance bias)	Low risk	Quote: "The control group was fed with milk and a placebo that was physically indistinguishable from the probiotic powder"
Blinding of outcome assessment (detection bias)	Low risk	Quote: "After starting the feeding, infants were observed continuously by a chart containing basic information like daily weight, feeding volume, abdominal girth, appearance of erythema of abdominal wall, loose stools with blood, vomiting, and orogastric tube suction volume. The amount of feeding was advanced slowly, if tolerated, with no more than a 20 ml/kg/d"
Incomplete outcome data (attrition bias)	Low risk	Quote: "Feeding was discontinued if there was any sign of feeding intolerance (defined as the presence of gastric aspirate in the amount that was more than a half of the previous feeding or abdominal distension)"
Selective reporting (reporting bias)	Low risk	Authors seem to report all the relevant outcomes
Other bias	Low risk	No other risk of bias was noted

Demirel et al. (2013)

**Table:** 

**Methods**	Prospective, blinded, randomized control trial conducted in Turkey
**Participants**	**Inclusion criteria**: Neonates born ≤32 weeks and birthweight ≤1500 g who survived to start enteral feedings.
	**Exclusion criteria**: Major congenital anomalies, lack of parental consent, death in first seven days after study start
**Interventions**	**Intervention**: Probiotic supplementation: *S. boulardii*: Dose was 250 mg (5 billion cfu), added to breastmilk or formula, frequency was once daily and supplementation continued till discharge
	**Comparison**: Placebo
**Outcomes**	NEC, sepsis, mortality
**Notes**	Data was not analysed as intention to treat analysis. We created the intention to treat analysis by using the number randomized and the outcome numbers given in table 3

Risk of bias table

**Table:** 

Bias	Authors' judgement	Support for judgement
Random sequence generation (selection bias)	Low risk	Quote, "Randomisation was simple and unadjusted and was performed using sequential numbers generated at the computer centre of the NICU"
Allocation concealment (selection bias)	Low risk	Quote, "The allocations were sealed in opaque, sequentially numbered envelopes"
Blinding of participants and personnel (performance bias)	Low risk	Quote, "The supplements were prepared by personnel on the breast milk team following the instructions in the sealed envelope. These individuals were the only personnel who were aware of the group assignments, and they were not involved in the care of the infants"
Blinding of outcome assessment (detection bias)	Low risk	Quote, "The supplements were prepared by personnel on the breast milk team following the instructions in the sealed envelope. These individuals were the only personnel who were aware of the group assignments, and they were not involved in the care of the infants"
Incomplete outcome data (attrition bias)	Low risk	7/278 dropped out. The intervention and the control group were fairly similar
Selective reporting (reporting bias)	Low risk	Authors seems to report all the outcomes mentioned in the analysis plan
Other bias	Low risk	No other bias was noted

Dilli et al. (2015)

**Table:** 

**Methods**	Prospective, randomized, controlled trial conducted in TurkeyQuote
**Participants**	**Inclsuin criteria:**: "VLBW infants with a gestational age of <32 weeks and a birth weight of <1500 g, born at or transferred to the NICU within the 1st week of life and fed enterally before inclusion, were eligible
	**Exclusion criteria:** Infants with any disease other than those linked to prematurity or congenital anomalies of the intestinal tract, not fed enterally or who died before the seventh day after birth, whose mothers had taken nondietary probiotic supplements, and whose parents refused to participate were excluded"
**Interventions**	**Intervention:** Probitics/Prebiotics
	Multiple Arm trial
	1) Probiotic (*Bifiidobacterium lactis*, 5×10^9^ colony‐forming units) 2) Prebiotic (inulin, 900 mg) 3) Synbiotic (*Bifidobacterium lactis*, 5×10^9^ colony‐forming units, 30 mg plus inulin, 900 mg 4) **The control group**: maltodextrin powder as a placebo
	The dose was 1 sachet per day of pre/probiotics with breast milk or formula until discharge or death, for a maximum of 8 weeks, whichever comes first
**Outcomes**	NEC, sepsis and mortality
**Notes**	The data were taken from table 3. We included data as probiotics+synbiotics vs. prebiotics+placebo

Risk of bias table

**Table:** 

Bias	Authors' judgement	Support for judgement
Random sequence generation (selection bias)	Low risk	Quote: "Infants were randomized by balanced blocks using sealed envelopes"
Allocation concealment (selection bias)	Low risk	Quote: "Infants were randomized by balanced blocks using sealed envelopes"
Blinding of participants and personnel (performance bias)	Low risk	Quote: " In feeding units, sachets were opened and mixed with 1 ml of sterile water or breastmilk immediately before administration to infants who were receiving enteral feeding on the day of the supplementation. The feeding team was not involved in the care of the infant and followed directions on the sealed envelopes"
Blinding of outcome assessment (detection bias)	Low risk	Quote: "The only personnel who knew of the infants group assignments were the investigators". As the outcomes were mostly objective, it is less likely that study had significant detection bias
Incomplete outcome data (attrition bias)	Low risk	Minimal loss to follow up reasons reported
Selective reporting (reporting bias)	Low risk	Authors seem to report all the relevant outcomes
Other bias	Low risk	No other sources of bias were noted

Dutta et al. (2015)

**Table:** 

**Methods**	A randomized, placebo‐controlled trial conducted in India
**Participants**	**Inclusion criteria:**
	(1) Neonates born at 27–33 weeks gestation in our hospital (2) aged <96 h of life (3) who were likely to either remain admitted in hospital or reside within 30 km of the hospital for the next 28 days (4) who were tolerating at least 15 ml/kg/day of milk feeds
	**Exclusion criteria:**
	(1) a gastro‐intestinal malformation (2) prior NEC or sepsis (3) any life‐threatening malformation that limited estimated life expectancy to less than a month
**Interventions**	**Intervention group:** Probiotic *Lactobacillus acidophilus* (662.5 million), *Lactobacillus rhamnosus* (362.5 million), *Bifidobacterium longum* (87.5 million), and *Saccharomyces boulardii* (137.5 million)
	Four groups
	A. High‐dose long course 10^10^ cells 12 hourly for 21 days B. High‐dose short course 10^10^ cells 12 hourly for days 1–14; followed by placebo from days 15–21 C. Low‐dose long course 10^9^ cells 12 hourly for 21 days D. **Control group**: Placebo for 21 days
**Outcomes**	NEC, mortality and sepsis
**Notes**	We combined all the probiotics groups (group A + B + C) to avoid the double counting of the placebo.

Risk of bias table

**Table:** 

Bias	Authors' judgement	Support for judgement
Random sequence generation (selection bias)	Low risk	Quote: "A block randomized sequence was generated online by an investigator who was not involved in the recruitment of subjects"
Allocation concealment (selection bias)	Low risk	Quote: "A block randomized sequence was generated online by an investigator who was not involved in the recruitment of subjects"
Blinding of participants and personnel (performance bias)	Low risk	Quote: "The external appearance and the contents of the sachets of high dose, low dose, and placebo were identical looking.
Blinding of outcome assessment (detection bias)	Low risk	As the intervention was concealed properly, less likely that outcomes assessors were aware of the allocation
Incomplete outcome data (attrition bias)	Low risk	Minimal loss to follow up
Selective reporting (reporting bias)	Low risk	Authors seems to report all the relevant outcomes
Other bias	Low risk	No other risk of bias was noted

Edmond et al. (2015)

**Table:** 

**Methods**	Randomized, double‐blind, placebo‐controlled trial conducted in Ghana
**Participants**	Recruited from the community, at least 2 h old, able to tolerate oral feeds and the family was likely to stay in the area. Parental consent was needed for inclusion to study.
**Interventions**	Intervention: Vitamin A: single dose: each dose was 50,000 IU
	Control: Placebo
	Common intervention given to all groups: Vitamin E
**Outcomes**	Primary outcome: All‐cause mortality
	Other outcomes: Diarrhea, vomiting, bulging fontanelle, irritability, fever
**Notes**	Some of the participants were HIV positive

Risk of bias table

**Table:** 

Bias	Authors' judgement	Support for judgement
Random sequence generation (selection bias)	Low risk	Quote: “The computerised block randomisation scheme was done with a block size of 20, so that in each block ten infants received vitamin A and ten received placebo”
		Comment: Most likely done
Allocation concealment (selection bias)	Low risk	Quote: “An independent statistician who was not part of the trial prepared the randomisation code at the WHO offices in Geneva, Switzerland. The code was available only to the Data Safety and Monitoring Board (DSMB) and their statistician”
		Comment: Most likely done
Blinding of participants and personnel (performance bias)	Low risk	Quote: “The research team and parents were fully unaware of the content of the capsules, which were only labelled with the infant number. Amanufacturer (StridesArcolab Limited, Bangalore, India) supplied the capsules. Separate staff, who were not part of the trial, labelled all capsules”
		Comment: Most likely done
Blinding of outcome assessment (detection bias)	Unclear risk	Quote: "…"
		Comment: Most likely done
Incomplete outcome data (attrition bias)	Low risk	Quote: “Our loss to follow‐up was only 1.1% at the time of ascertainment of our primary outcome at 6 months and only 2.9% at 12 months”
Selective reporting (reporting bias)	Low risk	Authors seem to report all the relevant outcomes
Other bias	Low risk	No other risk of bias was noted

Fernández‐Carrocera et al. (2013)

**Table:** 

**Methods**	Propsective, double‐blind, randomized clinical trial conducted in Mexico
**Participants**	**Inclusion criteria**: Preterm newborns who weighed <1500 g admitted at the intensive and intermediate care units
	**Exclusion criteria**: Preterm newborns weighing <1500 g with a low Apgar score (<6 at 5 min), gastrointestinal malformations, genetic syndromes, asphyxia and IA–IB NEC stages according to Bell's
**Interventions**	**Intervention**: Probiotic supplementation: "Lacidophilus 1.0×10^9^ colony forming units (CFU) CFU/g, *Lactobacillus rhamnosus* 4.4×108 CFU/g, *Lactobacillus casei* 1.0×10^9^ CFU/g, *Lactobacillus plantarum* 1.76×108 CFU/g, *Bifidobacteruim infantis* 2.76×107 CFU/g, *Streptococcus theremophillus* 6.6×105 CFU/g, each pack (Laboratorio Italmex SA)"
	**Control**: "The control group received their regular feeds from their mother's own milk when available with nothing added, or a premature infant formula"
**Outcomes**	All‐cause mortality, NEC
**Notes**	Data were taken from table 3

Risk of bias table

**Table:** 

Bias	Authors' judgement	Support for judgement
Random sequence generation (selection bias)	Low risk	Infants were prospectively and randomly assigned to one of two groups using a random digit table
Allocation concealment (selection bias)	Low risk	"which was handled by the Human Milk Bank staff that was not involved in the care of the patients and adhered to proper trial procedures."
Blinding of participants and personnel (performance bias)	Low risk	"As allocation concealment measure, the study group received a suspension that matched the physical appearance of milk and the bottles were labelled only with the patient's name and identification number as usual."
Blinding of outcome assessment (detection bias)	Low risk	Attending physicians and nurses caring for the infants were blinded to the group assignments.
Incomplete outcome data (attrition bias)	Low risk	Minimal loss to follow up
Selective reporting (reporting bias)	Low risk	Authors seem to report all the relevant outcomes
Other bias	Low risk	No other risk of bias was noted

Giridhar et al. (2019)

**Table:** 

**Methods**	Randomized, parallel group, placebo controlled trial
**Participants**	**Inclusion criteria**: All infants admitted to the neonatal intensive care unit with birth weight between 750 and 1250 g and between 24 to 96 h of life
	**Exclusion criteria**: Lethal congenital malformations, terminal illness characterized by shock or bradycardia for more than 2 h, refusal of consent
**Interventions**	**Intervention 1**: Vitamin A: 5000 IU (0.125 ml) IM on alternate days till establishment of adequate enteral feeds followed by oral vitamin A 10,000 IU (1 ml) once daily for a total duration of 28 days
	**Control intervention 1**: Placebo: 0.125 ml 0.9% normal saline IM alternate days till establishment of adequate enteral feeds followed by 1 ml oral dose of inert pharmacy made substance once daily for a total duration of 28 days
**Outcomes**	Proportion of infants with vitamin A deficiency (plasma retinol <200 mcg/L), mortality, sepsis
**Notes**	

Risk of bias table

**Table:** 

Bias	Authors' judgement	Support for judgement
Random sequence generation (selection bias)	Low risk	Quote: "The random sequence was generated online from the web site www.randomizer. org"
Allocation concealment (selection bias)	Low risk	Quote: "Each stratum had permuted, even‐numbered, randomly varying block sizes"
Blinding of participants and personnel (performance bias)	Low risk	Quote: "The investigators, supervisors, caregivers, laboratory personnel, and statistician were blinded to the intervention"
Blinding of outcome assessment (detection bias)	Low risk	Quote: "The investigators, supervisors, caregivers, laboratory personnel, and statistician were blinded to the intervention"
Incomplete outcome data (attrition bias)	Low risk	Minimal loss to follow up
Selective reporting (reporting bias)	Low risk	Authors seem to report all the relevant outcomes
Other bias	Low risk	No other risk of bias was noted

Guney‐Varal et al. (2017)

**Table:** 

**Methods**	Prospective, randomized controlled trial conducted in Turkey
**Participants**	**Inclusion Criteria:** preterm infants with a gestational age ≤32 week and a birth weight ≤ 1500 g
	**Exclusion Crietria**: "detected chromosomal abnormalities, previous gastrointestinal system surgery, a diagnosis of metabolic disease, babies lost in the first postnatal week and babies with severe sepsis episode were excluded from the study"
**Interventions**	**Intervention:** Probiotic supplementation: Lactobacillus rhamnosus (4.1×10^8^ cfu) + Lactobacillus casei (8.2×108 cfu) + Lactobacillus plantorum (4.1×10^8^ cfu) + *Bifidobacterium animalis* (4.1×10^8^ cfu)
	**Comparison:** No probiotics
**Outcomes**	Mortality, NEC, sepsis
**Notes**	Data were taken from table 2

Risk of bias table

**Table:** 

Bias	Authors' judgement	Support for judgement
Random sequence generation (selection bias)	High risk	"Alternate randomization was used to enrol the infants to the study arms"
		This method of randomization is not adequate
Allocation concealment (selection bias)	High risk	Less likely to be done as the sequence generation was done on alternate basis
Blinding of participants and personnel (performance bias)	Unclear risk	No details are provided about blinding
Blinding of outcome assessment (detection bias)	Unclear risk	No details are provided about blinding
Incomplete outcome data (attrition bias)	Low risk	Minimal loss to follow up
Selective reporting (reporting bias)	Low risk	Authors seem to report all the relevant outcomes
Other bias	Low risk	No other risk of bias was noted

Hariharan et al. (2016)

**Table:** 

**Methods**	Prospective, randomized control trial conducted in India
**Participants**	**Inclusion criteria**: "Infants with birth weight <1250 g, gestation <32 weeks"
**Interventions**	**Intervention**: *Lactobacillus acidophilus*, *Bifidobacterium bifidum*, *Saccharomyces boulardii* 2.5 ×10^9^ UFC of each twice a day, from the 3rd day of life, for 6 week courses
	**Comparison:** No probiotic
**Outcomes**	Mortality, NEC, sepsis
**Notes**	Only abstract available

Risk of bias table

**Table:** 

Bias	Authors' judgement	Support for judgement
Random sequence generation (selection bias)	Unclear risk	No details were available as only the abstract was available
Allocation concealment (selection bias)	Unclear risk	No details were available as only the abstract was available
Blinding of participants and personnel (performance bias)	Unclear risk	No details were available as only the abstract was available
Blinding of outcome assessment (detection bias)	Unclear risk	No details were available as only the abstract was available
Incomplete outcome data (attrition bias)	Unclear risk	No details were available as only the abstract was available
Selective reporting (reporting bias)	Low risk	Minimal loss to follow up
Other bias	Unclear risk	No details were available as only the abstract was available

Hernández‐Enríquez et al. (2016)

**Table:** 

**Methods**	Prospective randomized controlled trial conducted in Mexico
**Participants**	**Inclusion criteria**: Infants with very low birth weight
**Interventions**	**Intervention:** *Lactobacillus reuteri* 5 drops, equivalent to 100 million colony forming units (1 × 10^8^ CFU) daily, whether they were newborns with weight >1000 to 1500 g. In the case of newborns with weight <1000 g them 3 drops were administered *Lactobacillus reuteri* (60 million CFU) daily
	The duration of supplementation was 20 days
	**Compasrion:** Group B (control group) received no probiotic.
**Outcomes**	NEC, sepsis
**Notes**	Study published in Spanish. Data extracted from the abstract

Risk of bias table

**Table:** 

Bias	Authors' judgement	Support for judgement
Random sequence generation (selection bias)	Unclear risk	Study published in Spanish. Details not available
Allocation concealment (selection bias)	Unclear risk	Study published in Spanish. Details not available
Blinding of participants and personnel (performance bias)	Unclear risk	Study published in Spanish. Details not available
Blinding of outcome assessment (detection bias)	Unclear risk	Study published in Spanish. Details not available
Incomplete outcome data (attrition bias)	Unclear risk	Study published in Spanish. Details not available
Selective reporting (reporting bias)	Unclear risk	Study published in Spanish. Details not available
Other bias	Unclear risk	Study published in Spanish. Details not available

Huaxian, 2013

**Table:** 

**Methods**	Randomized controlled trial conducted in China
**Participants**	**Inclusuion criteria**: Preterm babies admitted to NICU
**Interventions**	**Intervention**: Probiotics and early minimal feeding
	**Control**: Early minimal feedings
**Outcomes**	NEC
**Notes**	Only abstract was available

Risk of bias table

**Table:** 

Bias	Authors' judgement	Support for judgement
Random sequence generation (selection bias)	Unclear risk	No details were available as only the abstract was available
Allocation concealment (selection bias)	Unclear risk	No details were available as only the abstract was available
Blinding of participants and personnel (performance bias)	Unclear risk	No details were available as only the abstract was available
Blinding of outcome assessment (detection bias)	Unclear risk	No details were available as only the abstract was available
Incomplete outcome data (attrition bias)	Unclear risk	No details were available as only the abstract was available
Selective reporting (reporting bias)	Low risk	Minimal loss to follow up
Other bias	Unclear risk	No details were available as only the abstract was available

Humphrey et al. (1996)

**Table:** 

**Methods**	A placebo‐controlled trial conducted in Indonesia
**Participants**	**Inclusion criteria**: All infants born at Hasan Sadikin Hospital in Bandung, Indonesia from June 18, 1992, to June 3, 1993
	**Exclusion criteria**: Infants that were considered very low birthweight (<1500 g) and infants with life‐threatening conditions
	Total number randomized in the intervention group: 1034
	Total number randomized in the control group: 1033
**Interventions**	**Intervention**: 1 dose of 52 μmol of vitamin A (as retinyl palmitate) orally
	**Control**: Placebo (<0.10 μmol of vitamin A) orally
	Common intervention given to all groups: 23 μmol vitamin E (as *dl‐α‐*tocopherol) orally
**Outcomes**	**Primary outcome**: Infant Morbidity & Mortality
	**Other outcomes**: Diarrhea, fever, cough, rapid breathing, wheezing, otitis media, pneumonia, sepsis
**Notes**	

Risk of bias table

**Table:** 

Bias	Authors' judgement	Support for judgement
Random sequence generation (selection bias)	Low risk	Quote: "The randomization scheme and coded supplement packets were prepared by a team in Baltimore, none of whom was involved in recruitment or follow‐up of infants in Indonesia"
		Comment: Most likely done
Allocation concealment (selection bias)	Low risk	Quote: "Supplements were individually coded, odorless, and identical in appearance"
		Comment: Most likely done
Blinding of participants and personnel (performance bias)	Low risk	Quote: "The randomization scheme and coded supplement packets were prepared by a team in Baltimore, none of whom was involved in recruitment or follow‐up of infants in Indonesia
		Comment: Most likely done
Blinding of outcome assessment (detection bias)	Low risk	Quote:: "Two study pediatricians, masked to the treatment group of the case, independently reviewed each verbal autopsy and assigned as a probable cause of death all diagnoses for which the criteria of the algorithm were met"
		Comment: Most likely done
Incomplete outcome data (attrition bias)	Unclear risk	Total number of loss to follow up: n (%)
		The loss to follow up was balanced
Selective reporting (reporting bias)	Low risk	Authors seem to report all the relevant outcomes
Other bias	Low risk	No other risk of bias was noted

Hussain et al. (2016)

**Table:** 

**Methods**	Propsective, randomized controlled trial conducted in Pakistan
**Participants**	**Inclusion criteria**:
	1. Pre‐term neonates <36 weeks gestation 2. Low birth weight neonates <2.5 Kg 3. Both genders 4. Both NG feed and bottle feed neonates 5. All neonates that were admitted at day 1 of life
	**Exclusion criteria**:
	1. Neonates <30 weeks low birth weight neonates, <1.5 Kg 2. Neonate on mechanical ventilatory support 3. IUGR (gestational age>36 weeks and weight <2.5 kg 4. Patients with congenital cyanotic heart diseases or has birth asphyxia and persistent cyanosis and need of oxygen inhalation.
**Interventions**	**Intervention:** Probiotic supplementation: Bifidobacteria.
	**Comparison**: No probiotics
**Outcomes**	NEC
**Notes**	The strain of the probiotic was not clearly stated. The dose and duration of the intervention was not given

Risk of bias table

**Table:** 

Bias	Authors' judgement	Support for judgement
Random sequence generation (selection bias)	Low risk	Quote: "neonates were divided in two groups by using random number tables"
Allocation concealment (selection bias)	Unclear risk	Seems unlikely, but no clear statement is made regarding this matter
Blinding of participants and personnel (performance bias)	Unclear risk	No supporting statement is age in this regard
Blinding of outcome assessment (detection bias)	Unclear risk	No clear statement is made in this regard
Incomplete outcome data (attrition bias)	Low risk	Minimal loss to follow up
Selective reporting (reporting bias)	Low risk	Authors seem to report the relevant outcomes
Other bias	Low risk	No other source of bias was noted

Kaban et al. (2019)

**Table:** 

**Methods**	A double‐blind randomized controlled clinical trial conducted in Indonesia
**Participants**	**Inclusion criteria**: Gestational age of 28–34 weeks, birth weight of 1000–1800 g in a stable condition
	**Exclusion criteria**: Lower gastrointestinal tract obstruction, massive gastrointestinal tract bleeding, NEC, sepsis and shock, and refusal of the infants' parents to participate in the study
**Interventions**	**Intervention**: *Lactobacillus reuteri* DSM 17938, duration of at least 7 days or until the subject was discharged, experienced NEC, or died, five drops per day, 10^8^ colony‐forming units/day
	**Comparison**: Placebo: The placebo contains a mixture of pharmaceutical‐grade medium‐chain triglycerides and sunflower oil together with pharmaceutical‐grade silicon
**Outcomes**	Mortality, sepsis and death
**Notes**	Data were taken from table 4

Risk of bias table

**Table:** 

Bias	Authors' judgement	Support for judgement
Random sequence generation (selection bias)	High risk	Quote: "Subjects were allocated to the groups by a third party using a simple alternating randomization technique"
		Comment: Alternation allocation of patients is not random
Allocation concealment (selection bias)	Unclear risk	Quote: "Subjects were allocated to the groups by a third party using a simple alternating randomization technique"
		Comment: It is not clear if the allocation was revealed before the patient were allocated to intervention or placebo
Blinding of participants and personnel (performance bias)	Unclear risk	Authors mentioned that it was a double blind trial but no details were provided on how the blinding was done
Blinding of outcome assessment (detection bias)	Unclear risk	Authors mentioned that it was a double blind trial but no details were provided on how the blinding was done
Incomplete outcome data (attrition bias)	Low risk	No attrition was reported
Selective reporting (reporting bias)	Unclear risk	Authors seem to report all the relevant outcomes
Other bias	Unclear risk	No other risk of bias was noted

Klemm et al. (2008)

**Table:** 

**Methods**	Community‐based, double‐masked, cluster‐randomized, and placebo‐controlled trial conducted in Bangladesh
**Participants**	**Inclusion criteria**: Infants born to consenting mothers who were participating in the parent trial
	**Exclusion criteria**: Infants of consenting mothers who had died before they could be supplemented by staff, infants born outside of the study area, and infants who could not be reached to receive a supplement during the first 30 days after birth
	Total number randomized in the intervention group: 8525
	Total number randomized in the control group: 8591
**Interventions**	**Interventio**n: 50,000 IU vitamin A
	**Control**: Placebo
	Common intervention given to all groups:
**Outcomes**	**Primary outcome**: All‐Cause infant mortality
	**Other outcomes**: Bulging fontanel
**Notes**	– "Follow‐up of the trial cohort at 3 years of age revealed no evidence of adverse effects associated with having had a perinatal bulging fontanel in terms of cognitive, motor, and behavioral test outcomes" – This study was concluded early by direction of the Data Safety and Monitoring Board

Risk of bias table

**Table:** 

Bias	Authors' judgement	Support for judgement
Random sequence generation (selection bias)	Low risk	Quote: "Sectors were listed in geographically contiguous order and were randomized in blocks of 4 within each of 3 previously randomized maternal supplementation trial treatment arms…"
		Comment: Most likely done
Allocation concealment (selection bias)	Unclear risk	Quote: "Community maps of the area were developed, homes were issued numeric addresses, and married women of reproductive age were enumerated and issued unique study identification numbers"
		Comment: Most likely done
Blinding of participants and personnel (performance bias)	Low risk	Quote: "The supplements for both groups were opaque gelatinous capsules identical in shape, size, and color containing edible oil"
		Comment: Most likely done
Blinding of outcome assessment (detection bias)	Unclear risk	Quote: "Infant vital status was assessed weekly at home for the first 12 weeks of life by field staff and then again at 24 weeks of age"
		Comment: Most likely done
Incomplete outcome data (attrition bias)	Low risk	Total number of loss to follow up: 11 (0.07%)
		The loss to follow up was not balanced
Selective reporting (reporting bias)	Low risk	Authors seem to report all the relevant outcomes
Other bias	Low risk	No other risk of bias was noted

Malaba et al. (2005)

**Table:** 

**Methods**	Randomized, placebo‐controlled, 2‐by‐2 factorial design trial was conducted in Zimbabwe
**Participants**	**Inclusion criteria**: Neither the mother nor the infant had an acutely life‐threatening condition, the infant was a singleton with a birth weight of >1500 g, and the mother planned to stay in the region after delivery
	**Exclusion criteria**: Either the mother and/or the infant had an acutely life‐threatening condition, the infant was not a singleton or had a birth weight of <1500 g, and the mother did not plan to stay in the region after delivery
	Total number randomized in the intervention group:
	– Aa: 3529 – Ap: 3529 – Pa: 3530
	Total number randomized in the control group: Pp: 3522
**Interventions**	**Intervention**:
	– Mothers received 400,000 IU vitamin A (as retinyl palmitate) and infants received 50,000 IU vitamin A (Aa group) – Mothers received 400,000 IU vitamin A and infants received placebo (Ap group) – Mothers received placebo and infants received 50,000 IU vitamin A (Pa group)
	**Control**: Both mothers and infants received placebo (Pp group)
	Common intervention given to all groups: Soy oil base with vitamin E as a preservative (50 IU per maternal capsule; 10 IU per infant capsule)
**Outcomes**	**Primary outcome**: Infant mortality
	**Other outcomes**: None
**Notes**	

Risk of bias table

**Table:** 

Bias	Authors' judgement	Support for judgement
Random sequence generation (selection bias)	Low risk	Quote: "A separate team at Johns Hopkins University prepared the study capsule packets. Study identification numbers were randomly allocated to the treatment groups by computer in blocks of 12"
		Comment: Most likely done
Allocation concealment (selection bias)	Low risk	Quote: "Lists linking the study number to the treatment were kept in sealed envelopes and encrypted computer files"
		"Treatment and placebo capsules appeared identical…"
		Comment: Most likely done
Blinding of participants and personnel (performance bias)	Unclear risk	Quote: "Treatment and placebo capsules appeared identical…"
		Comment: Most likely done
Blinding of outcome assessment (detection bias)	Low risk	Quote: "Cause of death was determined from medical records for infants who died in a hospital or from a review of verbal autopsy information by a study pediatrician, who was masked to treatment group, for infants dying at home"
		Comment: Most likely done
Incomplete outcome data (attrition bias)	Unclear risk	Total number of loss to follow up: n (%)
		The loss to follow up was not balanced
Selective reporting (reporting bias)	Low risk	Authors seem to report all the relevant outcomes
Other bias	Low risk	No other risk of bias was noted

Masanja et al. (2015)

**Table:** 

**Methods**	A randomized, double‐blind, placebo‐controlled trial conducted in Tanzania
**Participants**	**Inclusion criteria**: Able to feed orally, parents planned to stay in the study area for at least 6 months, and informed written consent was provided
	**Exclusion criteria**: Infants enrolled in another trial
	Total number randomized in the intervention group: 15,995
	Total number randomized in the control group: 16,004
**Interventions**	**Intervention**: 50,000 IU of vitamin A (as retinol palmitate) orally
	**Control**: Placebo (minute amounts of vitamin E (9.5–12.6 IU) in soybean oil) orally
	Common intervention given to all groups: Minute amounts of vitamin E (9.5–12.6 IU) in soybean oil
**Outcomes**	**Primary outcome**: Mortality between supplementation and 6 months of age
	**Other outcomes**: Mortality between supplementation and 28 days of age, Mortality between supplementation and 365 days of age, and hospital admission in the first 6 months of life
**Notes**	

Risk of bias table

**Table:** 

Bias	Authors' judgement	Support for judgement
Random sequence generation (selection bias)	Low risk	Quote: "We randomly assigned infants to receive either vitamin A or a placebo. The unit of randomisation was the individual infant. Block randomisation was done at WHO (Geneva, Switzerland) in block sizes of 20 (ten infants received vitamin A and ten received placebo)"
		Comment: Most likely done
Allocation concealment (selection bias)	Low risk	Quote: "The vitamin A and placebo capsules were identical in taste and appearance. Capsules were individually packed in blister packs of two capsules each; one for the dose and the second for the backup dose. Labels for the capsules were printed at WHO with country and infant study number in sequential order"
		Comment: Most likely done
Blinding of participants and personnel (performance bias)	Low risk	Quote: "Codes for the experimental regimens were kept with the data and safety monitoring board and broken during the analysis after a cleaned and locked database for the study was submitted to WHO"
		Comment: Most likely done
Blinding of outcome assessment (detection bias)	Low risk	Quote: "All reported deaths of children were investigated and trained field staff visited the family at least 6 weeks after the date of death to do a verbal autopsy interview"
		"Trained field interviewers visited enrolled infants at home (or in health facilities for cases in which the mother and child were not discharged after delivery) 1 day and 3 days after dosing to monitor possible adverse events after supplementation"
		Comment: Most likely done
Incomplete outcome data (attrition bias)	Unclear risk	Total number of loss to follow up: n (%)
		The loss to follow up was not balanced
Selective reporting (reporting bias)	Low risk	Authors seem to report all the relevant outcomes
Other bias	Low risk	No other risk of bias was noted

Mazumder et al. (2015)

**Table:** 

**Methods**	Randomized, double‐blind, placebo‐controlled trial conducted in India
**Participants**	**Inclusion criteria**: Livebirths born in the study area
	**Exclusion criteria**: Died before screening, serious illness, and/or were admitted into the intensive care unit
	Total number randomized in the intervention group: 22,493
	Total number randomized in the control group: 22,491
**Interventions**	**Intervention**: 50,000 IU vitamin A plus vitamin E 9.5–12.6 IU
	**Control**: Placebo (vitamin E 9.5–12.6 IU)
	Common intervention given to all groups: Vitamin E 9.5–12.6 IU
**Outcomes**	**Primary outcome**: Infant mortality from supplementation to 6 months
	**Other outcomes**: Neonatal mortality, mortality between supplementation and 12 months of age, infant hospital admission one or more times due to any illness between supplementation and 6 months of age, potential adverse events in the 3‐day period following supplementation, and vitamin A status in a sub‐sample of infants at 2 weeks and 3 months of age
**Notes**	

Risk of bias table

**Table:** 

Bias	Authors' judgement	Support for judgement
Random sequence generation (selection bias)	Low risk	Quote: "The unit of randomisation was the individual infant. We randomly assigned infants using a block randomisation scheme with a block size of 20, so that in each block ten infants received vitamin A and ten received placebo. The randomisation list was prepared offsite at WHO (Geneva, Switzerland) by a statistician not otherwise involved with the trial"
		Comment: Most likely done
Allocation concealment (selection bias)	Low risk	Quote: "The vitamin A and placebo capsules were identical in colour, shape, and size. Capsules were individually packaged in identical blister packs with two capsules, one for the dose and the other as a backup"
		Comment: Most likely done
Blinding of participants and personnel (performance bias)	Low risk	Quote: "Investigators, participants' families, and the data analysis team were masked to treatment allocation"
		Comment: Most likely done
Blinding of outcome assessment (detection bias)	Low risk	Quote: "Research staff were trained to do surveillance, interview families, obtain informed consent, give capsules, collect baseline and follow‐up information, and data capture"
		Comment: Most likely done
Incomplete outcome data (attrition bias)	Low risk	Total number of loss to follow up: 40 (0.09%)
		The loss to follow up was not balanced
Selective reporting (reporting bias)	Low risk	Authors seem to report all the relevant outcomes
Other bias	Low risk	No other risk of bias was noted

Nandhini et al. (2016)

**Table:** 

**Methods**	A prospective, double blind. controlled trail conducted in India
**Participants**	**Inclusion criteria**: Preterm infants, enterally fed, 28–34 weeks, birthweight >1000 g, admitted to the NICU
	**Exclusion crietria**: major congenital anomalies, surgical problems of the GI tract, severe birth asphyxia, early onset sepsis
**Interventions**	**Intervention:** Synbiotic supplementation: probiotics (*Lactobacillus acidophilus* (700 million CFU), *Bifidobacterium longum* (400 million CFU), *Lactobacillus rhamnosus* (400 million CFU), *Lactobacillus plantaris* (300 million CFU), *Lactobacillus casei* (300 million CFU), *Lactobacillus bulgaricus* (300 million CFU), *Bifidobacterium infantis* (300 million CFU) and *Bifidobacterium breve* (300 million CFU) + Prebiotic (fructo‐oligosaccharide)
	The dose was given two times daily for 7 days. The Probitiotics were mixed with breastmilk
	**Comparison:** Standard of care without synbiotics
**Outcomes**	Mortality, neonatal sepsis, NEC
**Notes**	Data were included from table 2. We created the intervention to treat analysis

Risk of bias table

**Table:** 

Bias	Authors' judgement	Support for judgement
Random sequence generation (selection bias)	Low risk	After obtaining informed consent from the parents, neonates satisfying the inclusion criteria were randomized prior to starting enteral feeds into two groups using computer generated random numbers kept in opaque sealed envelopes
Allocation concealment (selection bias)	Low risk	After obtaining informed consent from the parents, neonates satisfying the inclusion criteria were randomized prior to starting enteral feeds into two groups using computer generated random numbers kept in opaque sealed envelopes
Blinding of participants and personnel (performance bias)	Unclear risk	No details are provided in the study
Blinding of outcome assessment (detection bias)	Unclear risk	No details are provided in the study
Incomplete outcome data (attrition bias)	Low risk	Miminal loss to follow up
Selective reporting (reporting bias)	Low risk	Author seem to report all the outcomes
Other bias	Low risk	No other bias was noted

Niekerk et al. (2015) (HIV exposed)

**Table:** 

**Methods**	A randomized, double blind, placebo‐controlled clinical trial conducted in South Africa
**Participants**	**Inclsuion criteria**: "(i) HIV‐positive or HIV‐negative mothers who gave birth to a premature and VLBW baby at TBCH and consented to participate in the study; (ii) only breastfeeding mothers, regardless of their HIV status; and (iii) HIV‐positive mothers that were on the prevention of mother to child transmission treatment schedule. Babies were included if they (i) had a birth weight of 500 g and 1250 g; (ii) were either HIV‐exposed or HIV‐unexposed; and (iii) received breast milk (either from their mothers or donor breast milk)"
	**Exclusion criteria:** abnormalities such as gastroschisis, a large omphalocele or congenital diaphragmatic hernia
**Interventions**	**Intervention:** Probiotics: L. rhamnosus GG [0.35 ×10^9 colony‐forming units (CFU)] and B. infantis (0.35 ×10^9^ CFU), 5 drops daily for 28 days
	**Control:** Placebo
**Outcomes**	Mortality and NEC
**Notes**	Data were taken from table 3. This was a multiple arm trial and we included the data for HIV exposed and HIV nonexposed separately

Risk of bias table

**Table:** 

Bias	Authors' judgement	Support for judgement
Random sequence generation (selection bias)	Low risk	Quote: "Participants were randomized into either the study or control groups (probiotic vs. placebo supplementation) with a random‐number sequence allocated to each participant number"
Allocation concealment (selection bias)	Low risk	Quote: "No differences in the colour and appearance of the probiotic and placebo were noted. The probiotic and placebo were blinded with the use of a colour‐coded label (orange or purple)"
Blinding of participants and personnel (performance bias)	Low risk	Quote: "The attending physician, nurses, researcher, research assistant and study participants were blinded to the group assignment"
Blinding of outcome assessment (detection bias)	Low risk	Quote: "The attending physician, nurses, researcher, research assistant and study participants were blinded to the group assignment"
Incomplete outcome data (attrition bias)	Low risk	Minimal loss to follow up
Selective reporting (reporting bias)	Low risk	Authors seem to report all the relevant outcomes
Other bias	Low risk	No other bias was noted

Niekerk et al. (2015) (HIV nonexposed)

**Table:** 

**Methods**	Same as above study
**Participants**	
**Interventions**	
**Outcomes**	
**Notes**	

Risk of bias table

**Table:** 

Bias	Authors' judgement	Support for judgement
Random sequence generation (selection bias)	Low risk	Quote: "Participants were randomized into either the study or control groups (probiotic vs. placebo supplementation) with a random‐number sequence allocated to each participant number"
Allocation concealment (selection bias)	Low risk	Quote: "No differences in the colour and appearance of the probiotic and placebo were noted. The probiotic and placebo were blinded with the use of a colour‐coded label (orange or purple)"
Blinding of participants and personnel (performance bias)	Low risk	Quote: "The attending physician, nurses, researcher, research assistant and study participants were blinded to the group assignment"
Blinding of outcome assessment (detection bias)	Low risk	Quote: "The attending physician, nurses, researcher, research assistant and study participants were blinded to the group assignment"
Incomplete outcome data (attrition bias)	Low risk	Minimal loss to follow up
Selective reporting (reporting bias)	Low risk	Authors seem to report all the relevant outcomes
Other bias	Low risk	No other bias was noted

Oncel et al. (2014)

**Table:** 

**Methods**	A prospective, double‐blinded, randomized, placebo controlled trial conducted in Turkey
**Participants**	**Inclusion criteria**: Preterm infants with a gestational age ≤32 weeks and birth weight ≤1500 g, which survived to feed enterally, were eligible for the study.
	**Exclusion crietria**: Major congenital malformations and lack of parental consent.
**Interventions**	**Intervention group:** Probiotic supplementation:Infants in the probiotic group received 5 drops of oil‐based suspension containing 1×10^8^ colony‐forming units of *Lactobacillus reuteri* DSM 17938". The probiotic was given once a day, until death or discharge from the hospital
	**Comparison:** Placebo
**Outcomes**	Mortality, NEC
**Notes**	We created the intention to treat analysis by using the numbers from fig. 1 and the table 2. We included all the patients who were randomized to intervention or control group

Risk of bias table

**Table:** 

Bias	Authors' judgement	Support for judgement
Random sequence generation (selection bias)	Low risk	Study infants were randomly assigned to probiotic or placebo by using sequential numbers generated at the computer centre of the NICU by 1:1 allocation ratio
Allocation concealment (selection bias)	Low risk	The allocations were contained in opaque, sequentially numbered sealed envelopes
Blinding of participants and personnel (performance bias)	Low risk	Identical vial containing only oil base were administered following the same protocol as the probiotic group.
Blinding of outcome assessment (detection bias)	Low risk	Seems less likely
Incomplete outcome data (attrition bias)	Low risk	Minimal loss to follow up
Selective reporting (reporting bias)	Low risk	Authors seem to report all the relevant outcomes
Other bias	Low risk	No other bias was noted

Panigrahi et al. (2017)

**Table:** 

**Methods**	A community‐based, double‐blind, placebo controlled randomized trial conducted in India
**Participants**	**Inclusion criteria**: Neonate >24 h and <96 h old, ≥2000 g at birth, breastfeeding begun by 24 h of life, ability to tolerate oral feeds, informed consent by parent or guardian
	**Exclusion criteria**: Evidence or suspicion of clinical sepsis before the infant was randomized, gestational age reported voluntarily by the mother to be <35 weeks, infant >96 h old, infant did not cry immediately after birth, mother had fever (>38°C) within 2 days of delivery, mother had foul‐smelling amniotic discharge within 2 days of delivery, mother had abdominal tenderness within 2 days of delivery, amniotic fluid was meconium‐stained, infant was on antibiotics, mother unlikely to stay in the village for 60 days, difficulty in carrying out study (maternal sickness etc.), or presence of major congenital anomalies (defined as any malformation that was felt to be life‐threatening or that required surgical intervention)"
**Interventions**	**Intervention**: Synbiotic supplementation: The synbiotic preparation consisted of a capsule containing ~109 *Lactobacillus plantarum* ATCC strain 202195 and 150 mg of fructooligosaccharide with 100 mg maltodextrin as excipient
	The synbiotic was administered orally to the newborns for 7 days beginning on day 2–4 of life
	**Comparison**: Placebo capsules contained only 250 mg of maltodextrin
**Outcomes**	Mortality, sepsis
**Notes**	This was a community based study. Data were taken from table 2

Risk of bias table

**Table:** 

Bias	Authors' judgement	Support for judgement
Random sequence generation (selection bias)	Low risk	Quote: "Each assignment was the product of a random permutation scheme that assigned 2 intervention and 2 placebo slots to each of 38 consecutive blocks of 4 assignments for each village. This numbered list and corresponding bar codes were created by the GCRC (General Clinical Research Center) at the University of Maryland with assistance from the Department of Bioinformatics"
Allocation concealment (selection bias)	Low risk	Quote: "This numbered list and corresponding bar codes were created by the GCRC (General Clinical Research Center) at the University of Maryland with assistance from the Department of Bioinformatics and given to the clinical trial supplier (Laxai USA, South Plainfield, New Jersey, USA) for labelling of the synbiotics and to prepare packages for each village to be assigned consecutively to enrolled subjects"
Blinding of participants and personnel (performance bias)	Low risk	Quote: "physicians had no access to randomization, distribution, or administration of the intervention making them completely blinded to the intervention"
Blinding of outcome assessment (detection bias)	Low risk	Quote: "physicians had no access to randomization, distribution, or administration of the intervention making them completely blinded to the intervention"
Incomplete outcome data (attrition bias)	Low risk	Minimal loss to follow up
Selective reporting (reporting bias)	Low risk	Authors seem to report all the relevant outcomes
Other bias	Low risk	No other risk of bias was noted

Rahmathullah et al. (2003)

**Table:** 

**Methods**	Community based, randomized, double‐blind, placebo controlled trial conducted in southern India
**Participants**	**Inclusion criteria**: Liveborn infants from all pregnancies in the participating villages
	**Exclusion criteria**: Stillbirths, Miscarriages, Any delivery more than 20 km outside the study area, and infants who died before the study team arrived
	Total number randomized in the intervention group: 6624
	Total number randomized in the control group: 6570
**Interventions**	**Intervention**: 24,000 IU vitamin A
	**Control**: Placebo
	Common intervention given to all groups: Edible oil solution
**Outcomes**	**Primary outcome**: Infant mortality at 6 months
	**Other outcomes**: None
**Notes**	

Risk of bias table

**Table:** 

Bias	Authors' judgement	Support for judgement
Random sequence generation (selection bias)	Low risk	Quote: "Randomisation was at the individual level, stratified by geographical area in blocks of four. Because births were likely in a variety of locations, randomisation was conducted at the time of recruitment"
		Comment: Most likely done
Allocation concealment (selection bias)	Low risk	Quote: "Treatment codes were kept in a sealed envelope in a locked filing cabinet in Baltimore"
		Comment: Most likely done
Blinding of participants and personnel (performance bias)	Low risk	Quote: "Investigators, study staff, and mothers were masked to the assigned treatment"
		Comment: Most likely done
Blinding of outcome assessment (detection bias)	Unclear risk	Quote: "Project staff visited the household every 2 weeks to assess the vital status of the child and any morbidity.
		"Investigators, study staff, and mothers were masked to the assigned treatment"
		Comment: Most likely done
Incomplete outcome data (attrition bias)	Unclear risk	Total number of loss to follow up: 143 (1.1%)
		The loss to follow up was not balanced
Selective reporting (reporting bias)	Low risk	Authors seem to report all the relevant outcomes
Other bias	Low risk	No other risk of bias was noted

Rehman et al. (2018)

**Table:** 

**Methods**	A randomized control conducted in Pakistan
**Participants**	**Inclsuion criteria**:"preterm infants having gestation of 27 to 36 + 7 weeks; they were VLBW (<1500 g) and they survived to feed enterally"
	**Exclusion criteria**: None available as only abstract was available for data extraction
**Interventions**	Authors mentioned that they supplemented the neonates with Probiotic mixtures. Exact strain was not clear
**Outcomes**	NEC
**Notes**	Study only available as abstract

Risk of bias table

**Table:** 

Bias	Authors' judgement	Support for judgement
Random sequence generation (selection bias)	Unclear risk	Study available as abstract
Allocation concealment (selection bias)	Unclear risk	Study available as abstract
Blinding of participants and personnel (performance bias)	Unclear risk	Study available as abstract
Blinding of outcome assessment (detection bias)	Unclear risk	Study available as abstract
Incomplete outcome data (attrition bias)	Unclear risk	Study available as abstract
Selective reporting (reporting bias)	Unclear risk	Study available as abstract
Other bias	Unclear risk	Study available as abstract

Rojas et al. (2012)

**Table:** 

**Methods**	A multicenter, double‐blinded, randomized, placebo‐controlled trial conducted in Colombia
**Participants**	**Inclusion Cciteria**: "Preterm infants admitted to NICU, birth weight ≤ 2000 g, haemodynamically stable (blood pressure not requiring boluses or pressors), and ≤ 48 h of age"
	**Exclusion criteria**: "Infants with evidence or suspicion of congenital intestinal obstruction or perforation, gastroschisis, large omphalocele, congenital diaphragmatic hernia, major congenital heart defects, or anticipated transfer to a NICU not participating in the study were excluded"
**Interventions**	**Intervention:** Probiotic administration:"Infants in the probiotic group received 5 drops of an oil‐based suspension containing 10^8^ colony‐forming units of L reuteri DSM 17938"
	**Comparison:** Oil based placebo
**Outcomes**	Mortality, NEC
**Notes**	Data were taken from table 2. The duration of the intervention was not clearly stated

Risk of bias table

**Table:** 

Bias	Authors' judgement	Support for judgement
Random sequence generation (selection bias)	Low risk	Quote: " Study participants were randomly assigned to probiotic or placebo by the use of a computer‐generated balanced block randomization scheme"
Allocation concealment (selection bias)	Low risk	Quote: "Treatment assignment was performed by using sealed, sequentially numbered, opaque envelopes, color‐coded for strata, available in each NICU pharmacy"
Blinding of participants and personnel (performance bias)	Low risk	Quote: "Infants were administered probiotic or placebo regardless of whether enteric feeds were started"
Blinding of outcome assessment (detection bias)	Low risk	The authors do not report clearly if the outcome assessment was blinded, however in the setting of adequate allocation concealment and blinding of interventions it is less likely the outcome assessors knew the intervention vs placebo group
Incomplete outcome data (attrition bias)	Low risk	Quote: "The study was terminated before the completion of the targeted sample because of a substantial drop in patient recruitment among participating institutions as well as funding restrictions that limited our ability to recruit the required additional subjects"
Selective reporting (reporting bias)	Low risk	Authors seem to report all the relevant outcomes
Other bias	Low risk	No other risk of bias was noted

Roy et al. (2014)

**Table:** 

**Methods**	A prospective, randomized, double blind, placebo‐controlled trial in India
**Participants**	**Inclusion criteria**: "Admission to the NICU, a stable oral feeding within 72 h of birth and an informed parental consent; gestational age (GA) < 37 weeks; birth weight <2500 g; adequate renal and liver function; a postnatal age <2 week; did not have baseline fungal colonization at enrolment (with colonization defined by isolation of fungi from a culture specimen obtained from any site during the first 3 days of life); did not receive any form of antifungal prophylaxis other than the probiotic used"
	**Exclusion criteria**: "presence of major congenital malformation; antenatal and perinatal risk factors for sepsis, major congenital malformation; stigma of congenital infection; severe lesions diagnosed by cranial ultrasound (e.g. intraventricular haemorrhage (IVH) grade 3 and 4 and major ischemic lesions); altered liver and renal function; likely to die within 72 h of birth; and babies of mothers taking supplemental probiotics by capsule/powder"
**Interventions**	**Intervention**: "6×109 CFU *Lactobacillus*: half a sachet of *Lactobacillus acidophilus* 1.25 billion, B. longum 0.125billion, B. bifidum 0.125billion, and B. lactis 1 billion/1 g sachet, daily for 6 weeks or NICU discharge
	**Control**:sterile water in breastmilk
**Outcomes**	NEC, mortality and sepsis
**Notes**	Data were taken from table 2

Risk of bias table

**Table:** 

Bias	Authors' judgement	Support for judgement
Random sequence generation (selection bias)	Low risk	Quote: "The newborns were randomized into two groups by a random‐generated (computer‐generated), predetermined number table"
Allocation concealment (selection bias)	Low risk	Quote: "All doctors, nurses, laboratory staff, and parents are blind to the randomized allocation"
		Less likely that allocation was revealed
Blinding of participants and personnel (performance bias)	Low risk	Quote: "All doctors, nurses, laboratory staff, and parents are blind to the randomized allocation"
Blinding of outcome assessment (detection bias)	Low risk	Quote: "All doctors, nurses, laboratory staff, and parents are blind to the randomized allocation"
Incomplete outcome data (attrition bias)	Low risk	Minimal loss to follow up
Selective reporting (reporting bias)	Low risk	Authors seem to report all the relevant outcomes
Other bias	Low risk	No other risk of bias was noted

Saengtawesin et al. (2014)

**Table:** 

**Methods**	A prospective, randomized control trial conducted in Thailand
**Participants**	**Inclusion criteria**: "all preterm infants with gestational age ≤34 weeks and birth weight ≤1500 g"
	**Exclusion criteria**: "Very low birth weight preterm infants who had severe birth asphyxia, chromosome anomalies, cyanotic congenital heart disease, congenital intestinal obstruction, gastroschisis, omphalocele, nil per oral >3 weeks and parents who declined consent"
**Interventions**	**Intervention**: Probiotics: "Infloran(1×10^9 *Lactobacillus acidophilus* and 1×109 *Bifidobacterium bifida*): 125 mg/kg/dose two times daily for 6 weeks
	**Control**: "unsupplemented breast milk or preterm formula"
**Outcomes**	Neonatal Sepsis, NEC
**Notes**	

Risk of bias table

**Table:** 

Bias	Authors' judgement	Support for judgement
Random sequence generation (selection bias)	Low risk	Quote: "randomized by blocks of four into two groups, study and control group"
		Comment: Most likely done
Allocation concealment (selection bias)	Low risk	Quote: "Infants in the control group were fed either breast milk or premature formula alone. Infloran® was stored in a refrigerator at 4°C to 8°C, at the hospital pharmacy and sent then to the neonatal unit according to prescription"
		Comment: The sequence was kept at a central location
Blinding of participants and personnel (performance bias)	Low risk	Seems less likely that participants were aware of the intervention
Blinding of outcome assessment (detection bias)	Low risk	Quote: "Whenever an infant was suspected to have NEC, he/she was evaluated by two attending neonatologist"
		Seems less likely that outcome assessment was biased
Incomplete outcome data (attrition bias)	Low risk	Minimal loss to follow up
Selective reporting (reporting bias)	Low risk	Authors seem to report all the relevant outcomes
Other bias	Low risk	No other risk of bias was noted

Samanta et al. (2009)

**Table:** 

**Methods**	A prospective randomized double‐blind control trial conducted in India
**Participants**	**Inclusion criteria**: "preterm (<32 weeks) VLBW (<1500 g) born between October 2007 and March 2008, started feed enterally, survived beyond 48 h of life"
	**Exclusion criteria**: "babies with major congenital and GI anomalies and babies who expired due to other neonatal illnesses"
**Interventions**	**Intervention**: Probitotics containing *Bifidobacteria infantis*, *Bifidobacteria bifidum*, *Bifidobacteria longum*, *Lactobacillus acidophilu*s: Dose 2.5 billion cfu each of 4 strains twice daily until NICU discharge
	**Control**: Breastmilk without probiotics
**Outcomes**	Mortality, sepsis
**Notes**	

Risk of bias table

**Table:** 

Bias	Authors' judgement	Support for judgement
Random sequence generation (selection bias)	Low risk	Quote: "the infants were randomly assigned to two groups by random number table sequence"
Allocation concealment (selection bias)	Unclear risk	No details are provided
Blinding of participants and personnel (performance bias)	Unclear risk	No details are provided
Blinding of outcome assessment (detection bias)	Unclear risk	No details are provided
Incomplete outcome data (attrition bias)	Low risk	Minimal loss to follow up
Selective reporting (reporting bias)	Low risk	Authors seem to report all the relevant outcomes
Other bias	Low risk	No other risk of bias was noted

Sari et al. (2011)

**Table:** 

**Methods**	Prospective, randomized, controlled trial Turkey
**Participants**	**Inclusion criteria**: Infants with gestational age of <33 weeks or birth weight of 1500 g
	**Exclusion criteria**: Major congenital malformations and lack of parental consent
**Interventions**	**Intervention**: "Lactobacillus sporogenes with a dose of 350 000 000 c.f.u. once a day until discharge"
	**Control**: "The control group was fed with breast milk or formula without the probiotics
**Outcomes**	NEC, mortality, sepsis
**Notes**	Data were taken from table 2 of the study. We created the intention to treat analysis

Risk of bias table

**Table:** 

Bias	Authors' judgement	Support for judgement
Random sequence generation (selection bias)	Low risk	Quote: "using sequential numbers generated at the computer center of…"
Allocation concealment (selection bias)	Low risk	Quote: "The allocations were contained in opaque, sequentially numbered sealed envelopes"
Blinding of participants and personnel (performance bias)	Low risk	Quote: "Thus, the only personnel who knew of the infants’ group assignments were the investigators and those in the breast‐milk team who were not involved in the care of the study infants"
Blinding of outcome assessment (detection bias)	Low risk	Quote: "Whenever an infant was suspected to have NEC, the infant was evaluated by two senior‐attending neonatologists who did not know the group assignment of the infant"
Incomplete outcome data (attrition bias)	Low risk	Minimal loss to follow up
Selective reporting (reporting bias)	Low risk	Authors seem to report all the outcomes irrespective of their statistical significance
Other bias	Low risk	No other risk of bias was noted

Serce et al. (2013)

**Table:** 

**Methods**	A prospective, double blind, placebo controlled trial conducted in Turkey
**Participants**	**Inclusion criteria**: "VLBW infants (gestational age ≤32 weeks; birth weight ≤1500 g) who survived to feed enterally were eligible for the trial"
	**Exclusion criteria**: "Infants who had severe asphyxia (stage III), major congenital anomalies, those who had been fasted for more than 3 weeks, died in the first postnatal 14 days and infants who used antifungal therapy were excluded"
**Interventions**	**Intervention**: "The study group received *Saccharomyces boulardii* (50 mg/kg equal to 0.5×10^9^ cell/kg per dose twice daily until discharge
	**Comparison**: placebo (distilled water; 1 ml per dose twice daily)
**Outcomes**	Mortality, NEC, sepsis
**Notes**	Data were taken from table 3

Risk of bias table

**Table:** 

Bias	Authors' judgement	Support for judgement
Random sequence generation (selection bias)	Low risk	Quote: "Randomization was performed by using sequential numbers generated at the computer…"
Allocation concealment (selection bias)	Low risk	Quote: "The allocations were contained in opaque, sequentially numbered sealed envelopes"
Blinding of participants and personnel (performance bias)	Low risk	Quote: "The allocations were contained in opaque, sequentially numbered sealed envelopes"
		Comment less likely that participants were aware
Blinding of outcome assessment (detection bias)	Unclear risk	No information was provided on who made the assessment
Incomplete outcome data (attrition bias)	Low risk	Minimal loss to follow up
Selective reporting (reporting bias)	Low risk	Authors seem to report all the outcomes irrespective of their statistical significance
Other bias	Low risk	No other risk of bias was noted

Shadkam et al. (2015)

**Table:** 

**Methods**	A prospective, triple blind, placebo controlled trial conducted in Iran
**Participants**	**Inclusion criteria**: "premature infants admitted at the neonatal intensive care unit (NICU) during October 2012‐March 2013. Gestational age of infants was estimated at 28–34 weeks using the Dubowitz method, and birth weight of infants was calculated to be 1000–1800 g"
	**Exclusion criteria**: "presence of disorders such as digestive obstruction, GI bleeding, gastroschisis, omphalocele, withdrawal syndrome, neonatal proven or clinical sepsis, congenital heart defect and asphyxia (degree II or III)"
**Interventions**	**Intervention**: "5 ml of a mixture containing *Lactobacillus reuteri* DSM 17938. One drop of this product holds a minimum of 20 million live *Lactobacillus reuteri* protectis." The intervention was given two times daily. The intervention was given until the child achieved full enteral feedings
	**Control**: "placebo group received 0.5 ml of distilled water every 12 hours"
**Outcomes**	Mortality, sepsis, NEC
**Notes**	Data were taken from table 2

Risk of bias table

**Table:** 

Bias	Authors' judgement	Support for judgement
Random sequence generation (selection bias)	Low risk	Quote: "using the random allocation software"
Allocation concealment (selection bias)	Unclear risk	No details were provided on where the allocation was placed
Blinding of participants and personnel (performance bias)	Low risk	Quote: "the intervention was implemented by nurses, and the physician was not aware of the condition of neonates in detail"
Blinding of outcome assessment (detection bias)	Low risk	Quote: "the intervention was implemented by nurses, and the physician was not aware of the condition of neonates in detail"
Incomplete outcome data (attrition bias)	Low risk	Minimal loss to follow up
Selective reporting (reporting bias)	Low risk	Authors seem to report all the outcomes irrespective of their statistical significance
Other bias	Low risk	No other risk of bias was noted

Shashidhar et al. (2017)

**Table:** 

**Methods**	A double blind randomized controlled trial in India
**Participants**	**Inclusion criteria**: "All neonates with a birth weight between 750 and 1499 g admitted to the NICU"
	**Exclusion criteria**: "Neonates with gastrointestinal anomalies, severe congenital malformation, and those not started on enteral feeds by day 14 of life were excluded"
**Interventions**	**Intervention**: "Multicomponent probiotic formulation of *Lactobacillus acidophilus*, *Lactobacillus rhamnosus*, *Bifidobacterium longum*, and *Saccharomyces boulardii* in the form of powdered sachets of 1 g each. The intervention was administered once a day at a dose of 1.25×109 CFU starting within 24 h of initiation of feeds. The probiotic supplementation was continued till discharge given once a day if the volume of feeds was 2 ml or more, and in two divided doses if the baby received <2 ml/feed
	**Control**: "The no probiotic group received only breast milk and served as the control"
**Outcomes**	Mortality, NEC
**Notes**	Data were taken from table 2

Risk of bias table

**Table:** 

Bias	Authors' judgement	Support for judgement
Random sequence generation (selection bias)	Low risk	Quote: "The subjects were randomly allocated into two groups using computer generated random numbers by an investigator not directly involved in the study"
Allocation concealment (selection bias)	Low risk	Quote: "Sequentially numbered opaque sealed envelopes were used for allocation concealment"
Blinding of participants and personnel (performance bias)	Low risk	Quote: "The two groups were coded as A and B and the group code was kept off site in an opaque sealed envelope and opened only after the final analysis was done"
Blinding of outcome assessment (detection bias)	Low risk	Quote: "The two groups were coded as A and B and the group code was kept off site in an opaque sealed envelope and opened only after the final analysis was done"
Incomplete outcome data (attrition bias)	Low risk	Minimal loss to follow up
Selective reporting (reporting bias)	Low risk	Authors seem to report all the outcomes irrespective of their statistical significance
Other bias	Low risk	No other risk of bias was noted

Dongol Singh et al. (2017)

**Table:** 

**Methods**	A randomized, double blind, placebo controlled conducted in Nepal
**Participants**	**Inclusion criteria**: Preterm babies admitted to the NICU
	**Exclusion criteria**: "Sick infants (neonates with clinical or proven sepsis), those with congenital malformation especially (central nervous system) malformation and other such as gastrointestinal obstruction, gastrointestinal bleeding, gastroschisis, omphalocoele, congenital heart defect and birth asphyxia (grade III). Out born babies were also excluded in this study"
**Interventions**	**Intervention**: "probiotics *Lactobacillus casei* var. rhamnosis (LCR 35) 0.8 mg (half packet) dissolved in 2 ml of EBM in infant more than 1500 g and 0.4 mg probiotics (1/4th packet) dissolved in 1 ml of EBM in infants <1500 g was given twice a day until they reached full feeding"
	**Control**: "placebo as expressed breast milk only"
**Outcomes**	NEC and mortality
**Notes**	Data were taken from the last two paragraphs of the result section

Risk of bias table

**Table:** 

Bias	Authors' judgement	Support for judgement
Random sequence generation (selection bias)	Low risk	Quote: "using random selection by lottery"
Allocation concealment (selection bias)	High risk	Quote: "Intervention was instructed by the researcher and conducted by nursing staff of NICU"
Blinding of participants and personnel (performance bias)	Low risk	Less likely that neonates were aware of the intervention
Blinding of outcome assessment (detection bias)	Unclear risk	No clear information was provided on who made the assessment for clinical outcomes
Incomplete outcome data (attrition bias)	Low risk	Minimal loss to follow up
Selective reporting (reporting bias)	Low risk	Authors seem to report all the outcomes irrespective of their statistical significance
Other bias	Low risk	No other risk of bias was noted

Sinha et al. (2015)

**Table:** 

**Methods**	Randomized, double‐blind, placebo‐controlled trial conducted in India in community settings
**Participants**	**Inclusion criteria**: infants aged 3 days, born in the hospitals weighing 1500–2500 g"
	**Exclusion criteria**: "extremely premature infants (<34 weeks), sick infants, those with congenital malformations incompatible with life, and those with guardians not giving consent and belonging to out of study areas"
**Interventions**	**Intervention**: "VSL#3 (a mix of eight strains: *Streptococcus thermophilus*, *Bifidobacterium breve*, *Bifidobacterium longum*, *Bifidobacterium infantis*, *Lactobacillus acidophilus*, *Lactobacillus plantarum*, *Lactobacillus paracasei*, and *Lactobacillus delbrueckii* spp bulgaricus, at a dose of 10 billion cfu for 30 days, starting on the third day of life and continued for 30 days"
	**Control**: "A similar‐looking maltodextrin preparation in the same outer packing was administered to the control group"
**Outcomes**	Mortality, sepsis
**Notes**	Data were taken from table 2 and table 4. For sepsis, we included the numbers for suspected sepsi

Risk of bias table

**Table:** 

Bias	Authors' judgement	Support for judgement
Random sequence generation (selection bias)	Low risk	Quote: "A team of scientists at INCLEN Trust, New Delhi, used a computer‐generated table for subject allocation"
Allocation concealment (selection bias)	Low risk	Quote: "Allocation concealment was ensured by sequentially numbering the sachet packets containing VSL#3 or placebo after block randomisation. Identical packaging of VSL#3 and a placebo with similar consistency and colour was provided"
Blinding of participants and personnel (performance bias)	Low risk	Quote: "Parents of enrolled infants, investigators and field workers were masked to treatment allocation. Data analysis was performed in a blinded manner"
Blinding of outcome assessment (detection bias)	Low risk	Quote: "Parents of enrolled infants, investigators and field workers were masked to treatment allocation. Data analysis was performed in a blinded manner"
Incomplete outcome data (attrition bias)	Low risk	Minimal loss to follow up
Selective reporting (reporting bias)	Low risk	Authors seem to report all the outcomes irrespective of their statistical significance
Other bias	Low risk	No other risk of bias was noted

Soofi et al. (2017)

**Table:** 

**Methods**	A cluster randomized, placebo controlled trial conducted in Pakistan
**Participants**	**Inclusion criteria**: All infants born in the study village were eligible for inclusion.
	**Exclusion criteria**: Infants with congenital anomalies were excluded
**Interventions**	**Intervention**: The intervention group received a single dose of vitamin A 50,000 IU
	**Comparison**: the comparison group received placebo.
	Both the groups received vitamin E.
**Outcomes**	All‐cause mortality, febrile illness, diarrhoea or pneumonia
**Notes**	

Risk of bias table

**Table:** 

Bias	Authors' judgement	Support for judgement
Random sequence generation (selection bias)	Low risk	Quote: “This was a cluster randomized, placebo‐controlled trial”; and “an external consultant generated the computerized allocation sequence of clusters to each study intervention to either group using Epi Info 3.5.3 with restricted randomization based on population size, expected births and LHW presence”
Allocation concealment (selection bias)	Low risk	Quote: “An external consultant generated the computerized allocation sequence of clusters to each study intervention to either group using Epi Info 3.5.3 with restricted randomization based on population size, expected births and LHW presence”
Blinding of participants and personnel (performance bias)	Low risk	Quote: “The capsules were identical in appearance (Banner Pharmacaps, Canada) and supplied through the courtesy of the Micronutrient Initiative (Ottawa, Canada). The capsules were packaged in containers labelled as A & B. The content of the capsules were masked from field staff and supervisors, and the codes were only known to the external consultant responsible for cluster randomizations and the chair of the DSMB. The masking was maintained until the completion of the study”
Blinding of outcome assessment (detection bias)	Low risk	Quote: “The capsules were identical in appearance (Banner Pharmacaps, Canada) and supplied through the courtesy of the Micronutrient Initiative (Ottawa, Canada). The capsules were packaged in containers labelled as A & B. The content of the capsules were masked from field staff and supervisors, and the codes were only known to the external consultant responsible for cluster randomization and the chair of the DSMB. The masking was maintained until the completion of the study”
Incomplete outcome data (attrition bias)	Low risk	Quote: “We were able to follow 10,286 (93%) infants until death or 6 months of age”
Selective reporting (reporting bias)	Low risk	Author seem to report all the relevant outcomes
Other bias	Low risk	No other risk of bias was noted

Sun et al. (2019)

**Table:** 

**Methods**	A prospective, randomized study conducted in China
**Participants**	**Inclusion criteria:** Admitted to the neonatal intensive care unit at a gestational age of 28 weeks, 96 h of age
	**Exclusion criteria**: Genetic metabolic diseases; congenital major abnormalities; congenital TORCH infections with overt signs at birth; terminal stage of illness (pH 7.0 or hypoxia with bradycardia 2 h); or the lack of parental consent
**Interventions**	**Intervention**: Vitamin A, 1500 IU/day and continued if the infant tolerated the milk, for 28 days or until discharge
	**Comparison**: Placebo
**Outcomes**	Mortality, NEC
**Notes**	

Risk of bias table

**Table:** 

Bias	Authors' judgement	Support for judgement
Random sequence generation (selection bias)	Low risk	Quote: "A blocked randomization method stratified by the neonatal intensive care unit size was used to assign infants to either the control or oral VA group"
Allocation concealment (selection bias)	Low risk	Quote: "solutions, which were only labelled with the study site and infant number"
Blinding of participants and personnel (performance bias)	Low risk	Quote: "The medical and nursing teams caring for the infants were thus completely unaware of the content of the solutions"
Blinding of outcome assessment (detection bias)	Low risk	Quote: "The medical and nursing teams caring for the infants were thus completely unaware of the content of the solutions, which were only labelled with the study site and infant number"
Incomplete outcome data (attrition bias)	Low risk	No loss to follow up was noted
Selective reporting (reporting bias)	Low risk	Authors seem to report all the relevant outcomes
Other bias	Low risk	No other risk of bias was noted

Tewari et al. (2015)

**Table:** 

**Methods**	A double‐blinded, placebo‐controlled, randomized trial conducted in India
**Participants**	**Inclusion criteria**: "Preterm neonates <34 weeks admitted to the NICU"
	**Exclusion criteria**:
	"i. Extramural preterm neonates >10 day age with clinical or lab marker of sepsis
	ii. Preterm babies with necrotizing enterocolitis (NEC) or an intestinal surgical anomaly
	iii. Preterm babies with a lethal congenital anomaly, dysmorphism or aneuploidy"
**Interventions**	**Intervention**: "*Bacillus clausii* containing 2×10^9^ spores in 5 ml minibottle in a dose of 2 ml per‐oral every 8 h mixed with the enteral feeds through orogastric tube or oral feeds, giving them 2.4×10^9^ spores per day. Probiotic supplementation was continued till postnatal age of 6 weeks, or till discharge or death or occurrence of LOS, whichever was earlier for babies in both"
	**Control**: "Babies in the placebo group received sterile water, 2 ml per‐oral every 8 h mixed with feeds"
**Outcomes**	Mortality, sepsis, NEC
**Notes**	Data were taken from table 3 and 4. We combined the numbers for very preterm and extreme preterm babies

Risk of bias table

**Table:** 

Bias	Authors' judgement	Support for judgement
Random sequence generation (selection bias)	Low risk	Quote: "Randomization was done using an online service (www.randomization.com)"
Allocation concealment (selection bias)	Low risk	Quote: "Serially numbered opaque sealed envelopes with the allocation were available with the in‐charge nurse of the NICU, who dispensed the intervention in a syringe for oral administration"
Blinding of participants and personnel (performance bias)	Low risk	Quote: "All probiotic and sterile water mini bottles were coded and labels concealed"
Blinding of outcome assessment (detection bias)	Low risk	Quote: "All the investigators were blinded to the intervention"
Incomplete outcome data (attrition bias)	Low risk	Minimal loss to follow up
Selective reporting (reporting bias)	Low risk	Authors seem to report all the outcomes irrespective of their statistical significance
Other bias	Low risk	No other risk of bias was noted

West et al. (1995)

**Table:** 

**Methods**	A randomized, double‐masked trial conducted in Nepal
**Participants**	**Inclusion criteria**: Infants 5 months of age or younger
	**Exclusion criteria**: Infants >5 months of age
	Total number randomized in the intervention group: 5832
	Total number randomized in the control group: 6086
**Interventions**	**Intervention**:
	– 15,000 RE (50,000 IU) vitamin A administered orally in approximately 3 drops of oil for neonates (<1 month old) – 30,000 RE (100,000 IU) vitamin A administered orally in approximately 6 drops of oil for infants ages 1–5 months old
	**Control**:
	– 75 RE (250 IU) administered orally for neonates (<1 month old) – 150 RE (500 IU) administered orally for infants ages 1–5 months old
	Common intervention given to all groups: Approximately 3.3 IU vitamin E per drop of oil
**Outcomes**	**Primary outcome**: Infant mortality, all‐cause mortality
	**Other outcomes**: Malnutrition, ALRI, diarrhea or dysentery, whooping cough, meningitis, sudden death
**Notes**	All analyses were completed on an intent‐to‐treat basis

##### Risk of bias table

**Table:** 

Bias	Authors' judgement	Support for judgement
Random sequence generation (selection bias)	Low risk	Quote: "After a random start, wards were systematically assigned, blocked on VDAs, for infants to receive…"
		Comment: Most likely done
Allocation concealment (selection bias)	Low risk	Quote: "…from gelatinous capsules of identical appearance"
		Comment: Most likely done
Blinding of participants and personnel (performance bias)	Low risk	Quote: "The protocol and procedures for the trial were reviewed and approved by the Nepal Medical Research Council, Kathmandu, and the Joint Committee on Clinical Investigation at the Johns Hopkins University School of Medicine, Baltimore"
		Comment: Most likely done.
Blinding of outcome assessment (detection bias)	Unclear risk	Quote: "Verbal autopsy reports were independently reviewed by two physicians (SKK and RA) who standardized their reviews for ‘S0 pre‐study death reports"
		Comment: Most likely done
Incomplete outcome data (attrition bias)	Unclear risk	Total number of loss to follow up: n (%)
		The loss to follow up was not balanced
Selective reporting (reporting bias)	Low risk	Authors seem to report all the outcomes irrespective of their statistical significance
Other bias	Unclear risk	No other risk of bias was noted

Xu et al. (2016)

**Table:** 

**Methods**	A prospective, randomized, case‐controlled trial conducted in China
**Participants**	**Inclusion criteria**: "hospital‐born formula‐fed infants with a gestational age of 30–37 weeks and a birth weight between 1500 and 2500 g"
	**Exclusion criteria**: "severe neonatal pathologies, such as severe birth complications, GI malformations, chromosomal abnormalities, known immunodeficiency, hydrops fetalis, central venous catheter, antifungal drugs, and probiotics"
**Interventions**	**Intervention**: "The intervention group received *Saccharomyces boulardii* CNCM I‐745, administered two times per day as separate medication, not mixed with formula, at a dosage of 50 mg/kg. The study period ended at the 28th day after birth or when the infant was discharged from the hospital. Minimum duration of intervention was 7 days"
	**Control**: "Nothing was administered to the control group"
**Outcomes**	Neonatal sepsis
**Notes**	Data were taken from table 2

Risk of bias table

**Table:** 

Bias	Authors' judgement	Support for judgement
Random sequence generation (selection bias)	Low risk	Quote: "Randomization was conducted according to a random computer‐determined allocation order considering birth weight"
Allocation concealment (selection bias)	Low risk	Quote: "Blinding was possible because the nursing staff who administered S. boulardii to the infants was not involved in the daily care and the attending neonatal team was unaware of the randomization assignments"
		Comment: Less likely that randomization sequence was revealed
Blinding of participants and personnel (performance bias)	Low risk	Quote: "Blinding was possible because the nursing staff who administered S. boulardii to the infants was not involved in the daily care and the attending neonatal team was unaware of the randomization assignments"
Blinding of outcome assessment (detection bias)	Low risk	Quote: "Blinding was possible because the nursing staff who administered S. boulardii to the infants was not involved in the daily care and the attending neonatal team was unaware of the randomization assignments"
Incomplete outcome data (attrition bias)	High risk	Quote: "25 (20%) patients were considered dropouts"
Selective reporting (reporting bias)	Low risk	Authors seem to report all the outcomes irrespective of their statistical significance
Other bias	Low risk	No other risk of bias was noted

### Characteristics of excluded studies

**Table:** 

Abdulkadir et al. (2016)	
**Reason for exclusion**	Study conducted in a high income country (UK)
Abrahamse‐Berkeveld et al. (2016)	
**Reason for exclusion**	Study conducted in a high income country (Germany and Italy)
Abrahamsson et al. (2005)	
**Reason for exclusion**	No relevant outcomes were found
ADAPTS trial (2019)	
**Reason for exclusion**	Wrong settings: Ongoing study in Australia
Agarwal et al. (2003)	
**Reason for exclusion**	No relevant clinical outcomes were reported
Agarwal (2018)	
**Reason for exclusion**	Study conducted in a high income country (Australia)
Ahmadipour et al. (2019)	
**Reason for exclusion**	Study conducted to treat neonatal Jaundice.
Ahmadpour Kacho et al. (2005)	
**Reason for exclusion**	No relevant outcomes were reported
Al‐Hosni et al. (2012)	
**Reason for exclusion**	Study conducted in a high income country (USA)
Ala‐Houhala et al.(1988)	
**Reason for exclusion**	Study conducted in a high income country (Finland)
Allen et al. (2010)	
**Reason for exclusion**	Study conducted in high income country (UK)
Armanian et al. (2014)	
**Reason for exclusion**	Study participants were given only Prebiotics and no probiotics
Arthur et al. (1992)	
**Reason for exclusion**	Study population did not include Neonates
Aryayev et al. (2018)	
**Reason for exclusion**	Study conducted in high income country
Athalye‐Jape et al. (2018)	
**Reason for exclusion**	Study conducted in high income country
Awad et al. (2010)	
**Reason for exclusion**	Study was retracted
Ayah et al. (2007)	
**Reason for exclusion**	Vitamin A was given at 14 weeks
Aydin et al. (2012)	
**Reason for exclusion**	Population included children with Congential Heart disease only
Baglatzi et al. (2016)	
**Reason for exclusion**	Study conducted in a high income country
Bakker (2005)	
**Reason for exclusion**	Study continued supplementation of probiotics for 4 months
Bakker Zierikzee (2005)	
**Reason for exclusion**	Study conducted in a high income country (Netherland)
Bin‐Nun et al. (2005)	
**Reason for exclusion**	Study conducted in Israel
Bocquet et al. (2013)	
**Reason for exclusion**	Study conducted in a high income country (France)
**Bonati (1993a)**	
**Reason for exclusion**	Study conducted in a high income country (Italy)
**Bonati (1996b)**	
**Reason for exclusion**	Study conducted in a high income country (Italy)
Bora & Deori (2019)	
**Reason for exclusion**	Study compared two forms of the same intervention
Cekola et al. (2015)	
**Reason for exclusion**	Study conducted in a high income country (USA)
Chabra et al. (2013)	
**Reason for exclusion**	Study conducted in a high income country (USA)
Chandel et al. (2017)	
**Reason for exclusion**	No relevant clinical outcomes were available
Chi (2019)	
**Reason for exclusion**	No clinical outcomes were available
Chouraqui et al. (2008)	
**Reason for exclusion**	Study conducted in a high income country (France)
Chrzanowska‐Liszewska (2011)	
**Reason for exclusion**	Study conducted in a high income country (Poland)
Chua et al. (2017)	
**Reason for exclusion**	Study conducted in a high income country (Netherland)
Corkins & Kovacevich (2001)	
**Reason for exclusion**	Study conducted UK
Costalos et al. (2003)	
**Reason for exclusion**	Study conducted in a high income country (Greece)
**Costeloe (2016)**	
**Reason for exclusion**	Study conducted in a high income country (UK)
Coutsoudis et al. (1996)	
**Reason for exclusion**	No clinical outcomes were available
Dani et al. (2002)	
**Reason for exclusion**	Study conducted in high income country (
Darboe et al. (2007)	
**Reason for exclusion**	Study conducted on wrong study population (infants)
Delimont et al. (2019)	
**Reason for exclusion**	Study conducted on older children and used Sorghum‐Based and Corn‐Based Fortified Blended Foods
Delvin et al. (2000)	
**Reason for exclusion**	Study conducted in a high income country (US)
Deng & Chen (2010)	
**Reason for exclusion**	No abstract or full text available and no relevant outcomes were available.
Denkel et al. (2017)	
**Reason for exclusion**	Study conducted in a high income country (Germany)
**Deshpande 2016**	
**Reason for exclusion**	Study conducted in high income country
Diaby et al. (2018)	
**Reason for exclusion**	Observational study assessing the coverage of vitamin A supplementation
Dilli et al. (2013)	
**Reason for exclusion**	Study conducted on wrong patient population (infants with congenital heart diseases)
Elom et al. (2019)	
**Reason for exclusion**	Wrong study population
Escribano et al. (2018)	
**Reason for exclusion**	Study conducted in a high income country (Spain)
Galderisi et al. (2016)	
**Reason for exclusion**	The study investigated glucose monitoring and not the dextrose gel
Garg et al. (2017)	
**Reason for exclusion**	Wrong study design (retrospective cohort study)
Garland et al. (2011)	
**Reason for exclusion**	Study conducted in a high income country (Australia)
Garofoli et al. (2018)	
**Reason for exclusion**	Wrong setting
Gomber (1996)	
**Reason for exclusion**	Wrong study design
Gomez‐Rodriguez et al. (2019)	
**Reason for exclusion**	Compared two different regimens of probiotics. No placebo group was included.
Gonchar et al. (2016)	
**Reason for exclusion**	No relevant outcomes were available. Study only available in the form of abstract. Authors were contacted for full text but no response
Guo‐Qiang et al. (2016)	
**Reason for exclusion**	Wrong study design
Hammerman & Bin‐nun (2007)	
**Reason for exclusion**	Wrong intervention
Harris et al. (2016)	
**Reason for exclusion**	Study conducted in New Zealand
Hays (2015)	
**Reason for exclusion**	Study conducted in a high income country
Hoy‐Schulz et al. (2016)	
**Reason for exclusion**	Study conducted on wrong patient population
Hoyos (1999)	
**Reason for exclusion**	Wrong study design
Hua et al. (2014)	
**Reason for exclusion**	Only abstract available and no relevant clinical outcomes were available
Huang et al. (2016)	
**Reason for exclusion**	No relevant outcomes were available
Hunter et al. (2012)	
**Reason for exclusion**	Wrong study design
Härtel (2019)	
**Reason for exclusion**	The study is being conducted in a high income country (Germany)
Idindili et al. (2007)	
**Reason for exclusion**	Study conducted on wrong patient population
Indrio et al. (2008)	
**Reason for exclusion**	Study conducted in a high income country
IRCT (2015)	
**Reason for exclusion**	Wrong comparison
Jacobs (2017)	
**Reason for exclusion**	Study conducted in a high income country
Janvier et al. (2014)	
**Reason for exclusion**	Study conducted in a high income country
Kahbazi et al. (2019)	
**Reason for exclusion**	Wrong study population
Kanic et al. (2015)	
**Reason for exclusion**	Study conducted in a high income country
Karthikeyan & Bhat (2017)	
**Reason for exclusion**	Study conducted in a high income country
Kiatchoosakun (2014)	
**Reason for exclusion**	No relevant outcomes were available
Kirkwood et al. (2010)	
**Reason for exclusion**	Wrong study design
Kliegman (2005)	
**Reason for exclusion**	Wrong study design
Koksal et al. (2015)	
**Reason for exclusion**	Only abstract was available and no analyzable data were reported
Kukkonen et al. (2008)	
**Reason for exclusion**	Wrong study design
Leele et al. (2015)	
**Reason for exclusion**	Study conducted in a high income country (Singapore)
Li (2019)	
**Reason for exclusion**	The intervention continued for 4 months
Lin (2009)	
**Reason for exclusion**	Study conducted in a high income country (Taiwan)
Long & Dempsey (2018)	
**Reason for exclusion**	Study conducted in a developed country
Lozano (2008)	
**Reason for exclusion**	Study conducted in a high income country
Lund et al. (2014)	
**Reason for exclusion**	The control group did not receive the placebo but polio vaccine. It is difficult to tease out the effect of vitamin A supplementation vs. No vitamin A supplementation.
Lundelin et al. (2017)	
**Reason for exclusion**	Study conducted in a high income country
Mactier et al. (2012)	
**Reason for exclusion**	Study conducted in a high income country
Maldonado‐Lobon (2015)	
**Reason for exclusion**	Study conducted in a high income country
Manzano et al. (2017)	
**Reason for exclusion**	Study conducted in a high income country (Spain)
Manzoni et al. (2006)	
**Reason for exclusion**	Study conducted in a high income country (Italy)
Manzoni et al. (2009)	
**Reason for exclusion**	Study conducted in a high income country (italy)
Marissen et al. (2019)	
**Reason for exclusion**	This is an ongoing study in Germany which is a high income country.
Martins et al. (2009)	
**Reason for exclusion**	Wrong study population
Materna (2010)	
**Reason for exclusion**	Study conducted in a high income country
McCulloch et al. (2012)	
**Reason for exclusion**	Study conducted in a high income country (UK)
McKinlay (2016)	
**Reason for exclusion**	Study conducted in a high income country
Meyer & Gortner (2014)	
**Reason for exclusion**	Study conducted in a high income country (Germany)
Mg (2011)	
**Reason for exclusion**	Study conducted in a high income country (Italy)
Mihatsch et al. (2010)	
**Reason for exclusion**	Study conducted in a high income country (Germany)
Millar (2017)	
**Reason for exclusion**	Study conducted in a high income country
Moles et al. (2015)	
**Reason for exclusion**	Study conducted in high income country
Nadella et al. (2019)	
**Reason for exclusion**	Wrong intervention
Nct (2006)	
**Reason for exclusion**	Study conducted in high income country
Nct (2016)	
**Reason for exclusion**	Wrong settings
Papagaroufalis et al. (1991)	
**Reason for exclusion**	Study conducted in high income country (Greece)
Papagaroufalis et al. (2014)	
**Reason for exclusion**	Study conducted in high income country (Greece)
Patole et al. (2016)	
**Reason for exclusion**	Study conducted in a high income country (Australia)
Pearson et al. (1992)	
**Reason for exclusion**	Study conducted in a high income country (USA)
Plummer et al. (2018)	
**Reason for exclusion**	Study conducted in a high income country (Australia)
Puccio et al. (2007)	
**Reason for exclusion**	Study conducted in a high income country (Italy)
Qiao et al. (2017)	
**Reason for exclusion**	No relevant outcomes were available
Radke et al. (2017)	
**Reason for exclusion**	Study conducted in a high income country (Germany)
Raguž et al. (2016)	
**Reason for exclusion**	Wrong study design
Rakshasbhuvankar et al. (2017)	
**Reason for exclusion**	Study conducted in a high income country (Australia)
Rawat et al. (2016)	
**Reason for exclusion**	Study conducted in the USA
Repa et al. (2015)	
**Reason for exclusion**	Study conducted in a high income country (Austria)
Robbins & Fletcher (1993)	
**Reason for exclusion**	wrong comparator
Rodriguez‐Herrera et al. (2019)	
**Reason for exclusion**	Study conducted in a high income country
Rodríguez (2015)	
**Reason for exclusion**	Study compared two forms of Probiotics and no comparison with placebo was available
Rohan (2016)	
**Reason for exclusion**	Study conducted in a high income country (UK)
Ross et al. (1993)	
**Reason for exclusion**	Study included children 6 months and older
Rouge et al. (2009)	
**Reason for exclusion**	Study conducted in a high income country (France)
Rubaltelli et al. (2000)	
**Reason for exclusion**	Study conducted in a high income country (Italy)
Sadowska‐Krawczenko et al. (2012)	
**Reason for exclusion**	Study conducted in a high income country (Germany)
Samuels et al. (2016)	
**Reason for exclusion**	Study conducted in a high income country (Netherland)
Shenai et al. (1987)	
**Reason for exclusion**	Study conducted in a high income country (USA)
Smilowitz et al. (2017)	
**Reason for exclusion**	Study conducted in a high income country (USA)
Storm et al. (2019)	
**Reason for exclusion**	Study conducted in a high income country USA
Stratiki et al. (2007)	
**Reason for exclusion**	Study conducted in a high income country (Greece)
Strus et al. (2018)	
**Reason for exclusion**	Study conducted in a high income country (Poland)
Ter (2017)	
**Reason for exclusion**	Study conducted in Australia
Thanhaeuser et al. (2014)	
**Reason for exclusion**	Study conducted in a high income country (Austria)
Totsu et al. (2014)	
**Reason for exclusion**	Study conducted in a high income country (Japan)
Tyson et al. (1999)	
**Reason for exclusion**	Study conducted in a high income country (USA)
Venkatarao et al. (1996)	
**Reason for exclusion**	Infant received vitamin A at 6 months
Vlieger et al. (2009)	
**Reason for exclusion**	Study conducted in high income country (Netherland)
Wardle et al. (2001)	
**Reason for exclusion**	Study conducted in high income country (UK)
West et al. (1991)	
**Reason for exclusion**	Study included children 6‐59 months of age
Yang et al. (2011)	
**Reason for exclusion**	Full text not available and no abstract was available either so no relevant outcomes were available.

### Characteristics of studies awaiting classification

Barclay et al. (2003)

**Table:** 

**Methods**	Randomized controlled trial
**Participants**	Term infants
**Interventions**	Formula with pre‐pro and synbiotics
**Outcomes**	Growth
**Notes**	Study available only in the abstract form

Chubarova and Sharyafetdinova (2017)

**Table:** 

**Methods**	Randomized Controlled trial
**Participants**	Preterm neonates
**Interventions**	A combination of freeze‐dried strains of lactobacilli and bifidobacteria: *Lactobacillus rhamnosus*, *Bifidobacterium infantis*, *Bifidobacterium longum*, *Lactobacillus acidophilus* (at time of manufacture 2 billion CFUs per 3 g of the preparation), also containing the additional component maltodextrin. The comparison group received placebo
**Outcomes**	NEC and stay in NICU and others
**Notes**	Only abstract available. It is not clear on where the study was done

### Characteristics of ongoing studies

DelPiano (2016)

**Table:** 

**Study name**	Our open‐label, randomized controlled study has the primary endpoint of reducing diarrhea and infectious diseases (number of episodes/severity) and the secondary endpoint of decreasing infant mortality
**Methods**	Randomized control trial
**Participants**	The trial is currently conducted in Luzira, a suburb of Kampala, the capital of Uganda, and in Gulu and Lira, in the north of Uganda.The study is projected to enrol 4000 babies (control = 2000 and treatment=2000) who will be followed till 1 year of life. As controls, 2000 babies of the same community are planned to be considered
**Interventions**	The probiotic product selected for the trial is composed of 3 designated microorganisms, namely *Bifidobacterium breve* BR03 (DSM 16604), *B. breve* B632 (DSM 24706), and *Lactobacillus delbrueckii* subsp. delbrueckii LDD01 (DSM 22106)
**Outcomes**	Incidence of diarrhea and mortality
**Starting date**	
**Contact information**	Gastroenterology Department, Santa Rita Hospital‐Policlinico di Monza †Department of Gynecology and Obstetrics, Santa Rita Hospital‐Policlinico di Monza, Vercelli ‡Biolab Research Ltd, Novara, Italy
**Notes**	

Goodman (2015)

**Table:** 

**Study name**	Prevention of vitamin A deficiency by supplementation alongside routine vaccinations: a randomised controlled trial in Ghana infants
**Methods**	Randomised controlled trial
**Participants**	Participant inclusion criteria
	1. Mothers normally resident in the study area 2. Informed consent obtained from the mother
	Participant exclusion criteria
	1. Mothers unable to give informed consent 2. Mothers considered to be at high risk of adverse outcome in puerperal period 3. Multiple deliveries 4. Severe adverse reaction to vitamin A supplementation
**Interventions**	1st Group:
	1. Mothers 200,000 IU vitamin A shortly after delivery 2. Infants: 25,000 IU vitamin A with each Diphtheria, Pertussis, Tetanus (DPT) vaccine 1, 2 and 3
	2nd Group:
	1. Mothers 200,000 IU vitamin A at infant's Bacillus Calmette‐Guerin (BCG) vaccine and another 200,000 IU vitamin A at infant's 1st DPT 2. Infants: 50,000 IU vitamin A with each DPT 1, 2 and 3
**Outcomes**	Primary outcome measure
	1. Serum retinol levels, assessed by carrying out mRDR testing of infants at 6 weeks, 6 and 9 months 2. Modified Retinol Dose Response (mRDR) tests 3. Incidence of side effects such as bulging of the anterior fontanel and vomiting 4. Incidence of severe morbidity
	Secondary outcome measures
	1. Breast milk retinol concentrations, assessed at 6 weeks, 6 and 9 months for an assessment of the impact of the different supplementation regimes 2. mRDR testing of infants at 9 months of age
**Starting date**	01/01/2004
**Contact information**	World Health Organization
	Geneva‐27
	CH‐1211
	Switzerland
**Notes**	No results are available

Heydarabad (2018)

**Table:** 

**Study name**	Evaluation of the effect of probiotics on late‐onset sepsis in very preterm newborns
**Methods**	A randomized, triple blinded, placebo controlled clinical trial with two parallel groups
**Participants**	Inclusion criteria include: 1. Preterm infants weighting 1000‐1500 g and <32 weeks’ gestational age at birth 2. Enrolled within 48 h of birth
	Exclusion criteria: (a) Major congenital anomalies (Esophageal atresia,omphalocele, imperforate anus). (2) Major congenital heart malformations. (3) Genetic anomalies(e.g. Trisomy 21 or other trisomies). (4) Considered likely to die within 72 h of birth. (5) Death before minimal entral feeding (10‐20cc/kg/day). (6) Parents from whom informed consent cannot be obtained. (7) Sepsis in admission (CRP > 10 mg/dl in 1th day of admission). (8) Asphyxia (grade II, III). (9) Maternal chorioamnionitis
**Interventions**	Very low‐birth‐weight preterm infants with a gestational age of <32 weeks and a weight of 1000‐1500 gr who are admitted to the NICU of Shahid Motahari Hospital in Urmia during the first 48 h of their birth. Patients are randomly divided into two groups; the intervention group receive probiotic and the control group receive Dish water as placebo
**Outcomes**	Late onset sepsis
**Starting date**	2018‐03‐05
**Contact information**	Kamran Dehghan
	Iran (Islamic Republic of)
	dehghan.k@umsu.ac.ir
**Notes**	

Kaur (2018)

**Table:** 

**Study name**	Effect of probiotic supplementation on feed tolerance and weight gain in low birth weight infants on tube feeds
**Methods**	Randomized, parallel group trial
**Participants**	Inclusion criteria: All neonates with a birth weight between 1000 and 1800 gm admitted to the NICU in whom enteral feeds can be started.
	Exclusion criteria:
	Neonates with weight <1000 gm
	Neonates with gastrointestinal anomalies
	Neonates with major congenital malformations
	Neonates in whom the feed could not be started by day 14 of life
**Interventions**	Intervention 1: Probiotic: Probiotic containing *Lactibacillus acidophilus*, *Lactibacillus rhamnosus*, *Bifidobacterium longum*, *Saccharomyces boulardi* in form of powdered sachet of 1 g each
	Control Intervention 1: Nil: Nil
**Outcomes**	To compare the time taken (in days) to reach full enteral feeds (150 ml/kg/day) in low birth weight infants on orogastric feeds between the probiotic and no probiotic group
	To compare episodes of feed intolerance and weight gain in both the groups.Timepoint: Time taken to reach full enteral feeds
**Starting date**	31‐07‐2018
**Contact information**	Neonatal Intensive Care Unit, Department of pediatrics,GGS medical college and hospital faridkot
	Faridkot
	PUNJAB
	151203
	India
	dr.amarpreet12@gmail.com
**Notes**	

Londhe (2019)

**Table:** 

**Study name**	Use of zinc and pre‐probiotics as a therapeutic adjunct in neonatal sepsis in preterms‐ An Open label randomized controlled trial
**Methods**	Randomized, parallel group, multiple arm trial
**Participants**	All intramural preterm neonates from 28 week 1day to 36 week 6 days admitted to NICU at GMCH Aurangabad with proven sepsis during the study period, whose parents consented to be part of the study were included
	Diagnostic criteria for sepsis was
	(a) Positive “sepsis screen,” that is, presence of at least two of the following three parameters, namely, Total leucocyte count <5000/mm3, Low absolute neutrophil count (as per standard charts), C‐reactive protein>1 mg/dl, (b) Radiological evidence of pneumonia (c) Culture positive sepsis (d) Meningitis
**Interventions**	Zinc and prebiotics: zinc and pre‐probiotic group was given both zinc 10 mg per day and pre‐probiotics as syrup 5 ml per day containing *L. acidophilus* (1.25 billion), *B. longum* (0.125 billion), B. bifidum (0.125 billion), *B. lactis* (1 billion) and Inulin (25 mg) till discharge
	Zinc: Zinc group was given oral zinc 10 mg once a day irrespective of age of newborn till discharge.
	Pre‐biotic group: Pre‐probiotics group was given as Syrup 5 ml per day containing *L. acidophilus* (1.25 billion), *B. longum* (0.125 billion), *B. bifidum* (0.125 billion), *B. lactis* (1 billion) and Inulin (25 mg) till discharge
	Control: Not receiving any of the above
**Outcomes**	Reduction in mortality
**Starting date**	Date Completed: 31/08/2016
**Contact information**	Division of Neonatology Department of Pediatrics Govt Medical College Aurangabad
	431001
	Aurangabad, MAHARASHTRA
	India
	atul.londhe1982@gmail.com
**Notes**	Description results are available at the web site where the clinical trial was registered. We wrote to authors to obtain the results

Mirmohammdi (2018)

**Table:** 

**Study name**	Determination of the effect of probiotics on prevention of necrotizing enterocolitis in preterm infants
**Methods**	This randomized clinical trial will be conducted on preterm infants with low birth weight and very low birth weight.
**Participants**	Inclusion criteria: VLBW infants (gestational age ≤34 weeks and birth weight ≤1500 g) survive until the onset of oral nutrition
	Exclusion criteria: (1) Severe asphyxia (stage III) (2) Congenital major anomalies (3) Babies who have not started oral feeding for 3 weeks after birth. (4) Infants receiving antifungal treatment
**Interventions**	The intervention group consisted of newborn infants with probiotic podilakat made by Iran Fertilizer Company in a quantity of 1 drops per kg of weight every 12 h with breast milk or milk powder
	The control group is a neonate who will receive 0.5 cc normal saline every 12 h with breastfeeding or breastfeeding
**Outcomes**	Baby weight at the end of the 3rd month
**Starting date**	2019‐07‐29
**Contact information**	mir farhad mirmohammdi
	Ibn sina ave‐ imam reza hospital‐ mashhad‐ Iran
	Emailmirmohammadif951@mums.ac.ir
**Notes**	

Mukhtar, 2019

**Table:** 

**Study name**	Role of prophylactic microbial supplements in prevention of blood stream infection and intestinal tract injury in premature neonates
**Methods**	Randomized, parallel group trial
**Participants**	All infants admitted to our NICU with birth weight <2 kg or gestational age <35 weeks as assessed by EDD and further confirmed by Ballards score who survive the first 24 hrs of life will be enrolled in the study
**Interventions**	Mixture of *Lactobacillus acidophilus*, *Lactobacillus rhamnosus*, *Bifidobacterium longum*, and *Streptomyces boulardii* in a dose of 1.25 billion CFU twice daily started with the first feed which can be in the form of expressed breast milk or preterm formula feed and continued till discharge
	The control group will receive expressed breast milk or preterm formula feed with no supplements added
**Outcomes**	1. Necrotising colitis 2. Nosocomial sepsis
	In preterm low birth weight babies
**Starting date**	01/12/2012
**Contact information**	Seniour resident, Department of Pediatrics and Neonatology, Sher‐i‐Kashmir Institute of Medical Sciences, Soura Srinagar
	Srinagar
	JAMMU & KASHMIR
	190005
	India
	gousiamukhtar@gmail.com,
**Notes**	Study started in 2012 but no results have been published yet

***Nandhini 2012***

**Table:** 

**Study name**	A clinical study to analyse the effectiveness of administration of harmless bacteria in preventing infection of intestine in preterm babies
**Methods**	Randomized, parallel group trial
**Participants**	Inclusion criteria:
	birth weight >1000 g
	gestational age—28–34 weeks
	enterally fed
	Exclusion criteria:
	surgical conditions of gastrointestinal tract
	severe birth asphyxia
	major congenital malformations
	chromosomal anomalies
**Interventions**	Intervention 1: probiotics: *Bifidobacterium* and *Lactobacilli* species‐ capsule(available as pre pro HS capsules marketed by fourrts limited) one capsule, administered twice a day orally mixed with breast milk for 7 days
	Control Intervention 1: none
**Outcomes**	Incidence and severity of NEC. Timepoint: during period of hospital stay
**Starting date**	15‐02‐2011
**Contact information**	Department of pediatrics JIPMER, Dhanvanthri Nagar
	605006
	Pondicherry, PONDICHERRY
	India
	drnbiswal@yahoo.com
**Notes**	The web site said that the study is completed. We wrote to authors to ask for the results

Punnahitananda, 2011

**Table:** 

**Study name**	Effect of oral probiotic supplementation on the rate of hospital acquired infection and necrotizing enterocolitis in preterm very low birth weight infants
**Methods**	Randomized controlled study
**Participants**	Participant inclusion criteria
	1. VLBW preterm infants (gestational age (GA) < 35 weeks, body weight (BW) < 1.5 kg) 2. Admitted to the neonatal intensive care unit (NICU) who survived the first 3 days of life
	Participant exclusion criteria
	1. Infants with chromosome abnormality 2. Infants with severe congenital defects 3. Infants with gastrointestinal anomalies (e.g., omphalocele, gastroschisis, intestinal obstruction) 4. Infants with unstable hemodynamic status
**Interventions**	Daily enteral probiotic supplementation of live *Lactobacillus acidophilus* and *Bifidobacterium infantis* at a dose of 2.5 ×108 CFU of each strain once a day for at least 28 days versus placebo
**Outcomes**	Primary outcome measure
	Nosocomial Infections
	Secondary outcome measures
	1. Necrotizing enterocolitis (NEC) 2. Feeding tolerance 3. Time to reach full enteral feeding
**Starting date**	28/04/2011
**Contact information**	Rama IV Road
	Pathumwan
	Bangkok
	10330
	Thailand
	grad@chula.ac.th
**Notes**	

Rathod, 2019

**Table:** 

**Study name**	Probiotics for prevention of necrotising enterocoilitis in preterm neonates
**Methods**	Randomized, parallel group, placebo controlled trial
**Participants**	1. gestational age 28–34 weeks 2. Birth weight of <2 kg
	Exclusion criteria: Preterm newborn
	1. Birth weight >2 kg 2. Lethal Congenital malformation 3. Newborn on ventilator
**Interventions**	Intervention 1: giving probiotics: 50% of preterm newborn are given probiotics while rest 50% are not given probiotics
	Intervention 2: giving probiotics: 50% of preterm newborn are given probiotics while rest 50% are not given probiotics
	Control Intervention 1: not applicable
**Outcomes**	Occurrence of necrotising enterocolitis
**Starting date**	28/09/2017
**Contact information**	Mahendra Rathod
	Department of Pediatrics,sir Takhtsinhji hospital, Bhavnagar
	364001
	Bhavnagar, GUJARAT
	India
	jayendragohil@gmail.com
**Notes**	It is not clear if the study is completed.

Razavi, 2014

**Table:** 

**Study name**	Effect of probiotic in prevention of necroziting entrocolitis in preterm infants in Hafez hospital
**Methods**	Randomized, blinding: double blinded, placebo
**Participants**	Inclusion criteria: premature neonates with 1500 gr and below that are stable and tolerate (10cc/kg/day) formula or breast milk.Exclusion criteria: decline to participate; severe congenital anomaly; death
**Interventions**	Intervention group: add probiotics (Probiotic drops Pedilact Manufacturing zist takhmir) milk at the rate of 0/1cc/kg/day until hospital
	Control group: The control group was used as control and did not receive drug
**Outcomes**	Sign and symptom of NEC
**Starting date**	2014‐05‐22
**Contact information**	Dr.Seyed Mostajab Razavi
	Neonatal Research Center, Neonatal Department, Namazi Hospital, Zand Street, Shiraz Shiraz Iran (Islamic Republic of)
	porarish@sums.ac.ir
**Notes**	

Shashidhar, 2019

**Table:** 

**Study name**	A study of the effect of probiotic organism administration on feeding tolerance in very low birth weight newborn babies
**Methods**	Randomized, parallel group, active controlled trial
**Participants**	Inclusion criteria: All neonates (infants in the first 28 days of life) with a birth weight <1.5 kg, admitted to NICU SJMCH Bangalore. Postnatal age <2 wks and started enteral feeds.
	Exclusion criteria: Neonates GI tract anomalies, severe congenital malformations.
**Interventions**	Intervention 1: probiotic sachets: *Lactobacillus* Spp, *Bifidobacter* Spp, *Saccharomyces boulardi*
	oral 1 sachet once a day orally mixed in breast milk till discharge
	Control Intervention 1: breast milk: breast milk only
**Outcomes**	Incidence of feed intolerance
	Incidence of NEC stage 2 or more
	Duration of hospital stay
	Days on TPN
	Weight gain
	Mortality
**Starting date**	31‐08‐2012
**Contact information**	Senior resident Dept.of Neonatology St.Johns Medical College Koramangala Bangalore
	Bangalore
	KARNATAKA
	560034
	India
	shashiishere@gmail.com
**Notes**	

Sinha, 2019

**Table:** 

**Study name**	Phase III, multicentre, randomized, double‐blind, placebo‐controlled study to evaluate efficacy of probiotic supplementation for prevention of neonatal sepsis in 0–2 months old low birth weight infants in India
**Methods**	Randomized, parallel group, placebo controlled trial
**Participants**	Inclusion criteria:
	1. Birth weight: 1500 g to 500 g 2. Age of the new‐born Day 3‐7 on recruitment, that is, not later than 7th day 3. Stable clinical condition as assessed by physician and accepting feeds orally (where stable is defined as, does not require intravenous fluids and vasopressor medication to maintain circulation and accepts oral feeding or breastfeeding). 4. The mother (with the new‐born) is planning to stay in study area for a period of at least 2 months
	Exclusion criteria:
	1. New born with extreme prematurity, that is, <34 weeks 2. New born with illness requiring prolonged hospitalisation and interference with oral feeding 3. Presence of a gross congenital malformation incompatible with life 4. Parent or Legally authorized representative (LAR) not providing written consent
**Interventions**	Intervention 1: Vivomixx Drops, that is, Lactic acid bacteria and Bifidobacteria drop with medium chain triglyderide oil.
	1. Each bottle cap contains at least 50 Billion Lactic acid bacteria and Bifidobacteria a. *Streptococcus thermophilus* DSM 24731 b. *Bifidobacterium longum* DSM 24736, *B. breve* DSM 24732 and *B. infantis* DSM 24737) c. *Lactobacillus acidophilus* DSM 24735, *L. plantarum* DSM 24730, *L. paracasei* DSM 24733, and *L. delbrueckii* subs. bulgaricus DSM 24734 2. Each bottle contains MCT, that is, Medium chain triglyceride oil 5 ml.
	The contents of the cap should be mixed with MCT oil and shaken well prior to administration. Store in refrigerator at 2–8°C: 1 ml per day for 30 days (corresponding to NLT 10 billion CFU per day)
	Control Intervention 1: Placebo Drops
	1. Each Bottle cap contains Maltodextrin 2. Each bottle contains MCT, that is, Medium chain triglyceride oil 5 ml
	The contents of the cap should be mixed with MCT oil and shaken well prior to administration. Store in refrigerator at 2‐ 8 degrees centigrade.: 1 ml per day for 30 days
**Outcomes**	1. Sepsis 2. Possible serious bacterial infections (PSBIs). Timepoint: 60 days
**Starting date**	Date of first enrolment: 01‐12‐2019
**Contact information**	Division of Reproductive, Maternal and Child Health
	Ansari Nagar, New Delhi
	South
	DELHI
	110029
	India
**Notes**	Study started in Jan 2019

Summary of findings tables

**Additional tables**

## DATA AND ANALYSES

1. Vitamin A versus control

**Table:** 

**Outcome or subgroup**	**Studies**	**Participants**	**Statistical method**	**Effect estimate**
11. All‐cause neonatal mortality	6	126548	Risk ratio (IV, Random, 95% CI)	0.99 [0.90, 1.08]
12. All‐cause infant mortality at 6 months	12	154940	Risk ratio (IV, Random, 95% CI)	0.98 [0.89, 1.07]
13. All‐cause infant mortality at 12 months	8	118376	Risk ratio (IV, Random, 95% CI)	1.04 [0.94, 1.14]
14. Adverse events: bulging fontanelle	6	100256	Risk ratio (IV, Random, 95% CI)	1.53 [1.12, 2.09]
15. Adverse events: vomiting	5	99582	Risk ratio (IV, Random, 95% CI)	1.00 [0.93, 1.07]
16. All‐cause neonatal mortality: sensitivity analysis: fixed effect model	5	126242	Risk ratio (IV, Fixed, 95% CI)	0.99 [0.90, 1.08]
17. All‐cause infant mortality at 6 months: sensitivity analysis: fixed effect model	12	154940	Risk ratio (IV, Fixed, 95% CI)	0.97 [0.91, 1.03]
18. All‐cause infant mortality at 12 months: sensitivity analysis: fixed effect model	8	118376	Risk ratio (IV, Fixed, 95% CI)	1.02 [0.96, 1.08]

2. Probiotics versus control

**Table:** 

**Outcome or subgroup**	**Studies**	**Participants**	**Statistical method**	**Effect estimate**
21. All‐cause mortality	26	10998	Risk ratio (IV, Random, 95% CI)	0.80 [0.66, 0.96]
22. All‐cause mortality: subgroup analysis: settings	26	10998	Risk ratio (IV, Random, 95% CI)	0.80 [0.66, 0.96]
221. Hospital based	23	4691	Risk ratio (IV, Random, 95% CI)	0.78 [0.65, 0.94]
222. Community based	3	6307	Risk ratio (IV, Random, 95% CI)	1.25 [0.51, 3.05]
23. All‐cause mortality: subgroup analysis: type of probiotics	26	10998	Risk ratio (IV, Random, 95% CI)	0.80 [0.66, 0.96]
231. Preparation contain a single strain of probiotics	9	2242	Risk ratio (IV, Random, 95% CI)	0.80 [0.61, 1.05]
232. Preparation contained multiple strains of probiotics	12	3050	Risk ratio (IV, Random, 95% CI)	0.80 [0.58, 1.09]
233. Preparation contained synbiotics (prebiotics + probiotics)	5	5706	Risk ratio (IV, Random, 95% CI)	0.69 [0.29, 1.61]
24. All‐cause mortality: subgroup analysis: type of participants	26	10998	Risk ratio (IV, Random, 95% CI)	0.80 [0.66, 0.96]
241. Study include preterm/low birth weight babies	25	10587	Risk ratio (IV, Random, 95% CI)	0.79 [0.65, 0.95]
242. Study included term infants only	1	411	Risk ratio (IV, Random, 95% CI)	1.38 [0.31, 6.08]
25. All‐cause mortality: subgroup analysis: type of feedings	26	10998	Risk ratio (IV, Random, 95% CI)	0.80 [0.66, 0.96]
251. Baby received breastmilk only	14	7721	Risk ratio (IV, Random, 95% CI)	0.81 [0.62, 1.05]
252. Baby received formula milk only	1	411	Risk ratio (IV, Random, 95% CI)	1.38 [0.31, 6.08]
253. Baby recieved both both breastmilk and formula milk	8	2385	Risk ratio (IV, Random, 95% CI)	0.69 [0.48, 0.99]
254. Type of feeding was unclear	3	481	Risk ratio (IV, Random, 95% CI)	1.33 [0.63, 2.81]
26. All‐cause mortality: subgroup analysis: probiotics preparation	26	10998	Risk ratio (IV, Random, 95% CI)	0.80 [0.66, 0.96]
261. Preparation contained *Lactobacillus*	10	7002	Risk ratio (IV, Random, 95% CI)	0.82 [0.63, 1.05]
262. Preparation contained *Bifidobacterium*	1	400	Risk ratio (IV, Random, 95% CI)	0.43 [0.17, 1.09]
263. Preparation contained both *Lactobacillus* and *Bifidobacterium*	13	3110	Risk ratio (IV, Random, 95% CI)	0.71 [0.47, 1.08]
264. Preparation contained *Saccharomyces boulardii* only	2	486	Risk ratio (IV, Random, 95% CI)	1.12 [0.46, 2.71]
27. All‐cause mortality: sensitivity analysis: fixed effect models	26	10998	Risk ratio (IV, Fixed, 95% CI)	0.80 [0.66, 0.96]
28. All‐cause mortality: sensitivity analysis: risk of bias	26	10998	Risk ratio (IV, Random, 95% CI)	0.80 [0.66, 0.96]
281. High Risk of bias for randomisation/allocation concealment	3	285	Risk ratio (IV, Random, 95% CI)	0.27 [0.05, 1.40]
282. Low or Unclear Risk of bias for randomisation/allocation concealment	23	10713	Risk ratio (IV, Random, 95% CI)	0.82 [0.68, 0.99]
29. Necrotizing enterocolitis (any type)	30	5574	Risk ratio (IV, Random, 95% CI)	0.46 [0.35, 0.59]
210. Necrotizing enterocolitis: subgroup analysis: probiotic preparation	30	5574	Risk ratio (IV, Random, 95% CI)	0.46 [0.35, 0.59]
2101. Preparation contained *Lactobacillus*	13	2738	Risk ratio (IV, Random, 95% CI)	0.39 [0.25, 0.61]
2102. Preparation contained *Bifidobacterium*	1	400	Risk ratio (IV, Random, 95% CI)	0.20 [0.09, 0.47]
2103. Preparation contained both *Lactobacillus* and *Bifidobacterium*	14	1950	Risk ratio (IV, Random, 95% CI)	0.49 [0.36, 0.68]
2104. Preparation contained *Saccharomyces boulardii* only	2	486	Risk ratio (IV, Random, 95% CI)	0.94 [0.45, 1.95]
211. Necrotizing enterocolitis: subgroup analysis: type of feeding	30	5574	Risk ratio (IV, Random, 95% CI)	0.46 [0.35, 0.59]
2111. Baby receieved breastmilk only	13	1945	Risk ratio (IV, Random, 95% CI)	0.43 [0.31, 0.59]
2112. Baby receieved formula milk only	1	93	Risk ratio (IV, Random, 95% CI)	0.21 [0.03, 1.76]
2113. Baby received both breastmilk and formula milk	9	2445	Risk ratio (IV, Random, 95% CI)	0.55 [0.33, 0.92]
2114. Type of feeding was unclear	7	1091	Risk ratio (IV, Random, 95% CI)	0.41 [0.17, 1.00]
212. Necrotizing enterocolitis: subgroup analysis: type of probiotics	30	5574	Risk ratio (IV, Random, 95% CI)	0.46 [0.35, 0.59]
2121. Preparation contained a single strain of probiotics	12	2679	Risk ratio (IV, Random, 95% CI)	0.48 [0.30, 0.76]
2122. Preparation contained multiple strains of probiotics	15	2156	Risk ratio (IV, Random, 95% CI)	0.48 [0.35, 0.67]
2123. Preparation contained synbiotics (prebiotics + probiotics)	3	739	Risk ratio (IV, Random, 95% CI)	0.28 [0.12, 0.67]
213. Necrotizing enterocolitis: sensitivity analysis: fixed effect models	30	5574	Risk ratio (IV, Fixed, 95% CI)	0.45 [0.37, 0.56]
214. Necrotizing enterocolitis: sensitivity analysis: risk of bias	30	5574	Risk ratio (IV, Random, 95% CI)	0.46 [0.35, 0.59]
2141. Low or unclear risk of bias for randomisation/allocation concealment	27	5289	Risk ratio (IV, Random, 95% CI)	0.46 [0.35, 0.61]
2142. High risk of bias for randomisation/allocation concealment	3	285	Risk ratio (IV, Random, 95% CI)	0.33 [0.09, 1.13]
215. Neonatal sepsis	21	9105	Risk ratio (IV, Random, 95% CI)	0.78 [0.70, 0.86]
216. Neonatal sepsis: subgroup analysis: settings	21	9105	Risk ratio (IV, Random, 95% CI)	0.78 [0.70, 0.86]
2161. Hospital based	19	3209	Risk ratio (IV, Random, 95% CI)	0.83 [0.76, 0.91]
2162. Community based	2	5896	Risk ratio (IV, Random, 95% CI)	0.67 [0.49, 0.91]
217. Neonatal sepsis: type of probiotics	21	9105	Risk ratio (IV, Random, 95% CI)	0.78 [0.70, 0.86]
2171. Preparation contained single strain of probiotics	8	1328	Risk ratio (IV, Random, 95% CI)	0.84 [0.74, 0.96]
2172. Preparation contained multiple strains of probiotics	9	2482	Risk ratio (IV, Random, 95% CI)	0.81 [0.68, 0.97]
2173. Preparation contained synbiotics (prebiotics + probiotics)	4	5295	Risk ratio (IV, Random, 95% CI)	0.67 [0.54, 0.83]
218. Neonatal sepsis: subgroup analysis: type of feeding	21	9105	Risk ratio (IV, Random, 95% CI)	0.78 [0.70, 0.86]
2181. Baby received breastmilk only	8	6961	Risk ratio (IV, Random, 95% CI)	0.71 [0.61, 0.83]
2182. Baby received formula milk o nly	2	218	Risk ratio (IV, Random, 95% CI)	0.59 [0.22, 1.56]
2183. Baby received both formula and breastmilk only	7	1401	Risk ratio (IV, Random, 95% CI)	0.76 [0.64, 0.90]
2184. Type of feeding was unclear	4	525	Risk ratio (IV, Random, 95% CI)	0.95 [0.82, 1.09]
219. Neonatal sepsis: subgroup analysis: probiotic preparation	21	9105	Risk ratio (IV, Random, 95% CI)	0.78 [0.70, 0.86]
2191. Preparation contained *Lactobacillus*	11	7068	Risk ratio (IV, Random, 95% CI)	0.74 [0.62, 0.87]
2192. Preparation contained *Bifidobacterium*	1	400	Risk ratio (IV, Random, 95% CI)	0.81 [0.60, 1.09]
2193. Preparation contained both *Bifidobacterium* and *Lactobacillus*	6	1026	Risk ratio (IV, Random, 95% CI)	0.83 [0.68, 1.02]
2194. Preparation contained *Saccharomyces boulardii* only	3	611	Risk ratio (IV, Random, 95% CI)	0.73 [0.57, 0.94]
220. Neonatal Sepsis: Sensitivity analysis: Fixed Effect Model	21	9105	Risk ratio (IV, Fixed, 95% CI)	0.79 [0.73, 0.85]
221. Neonatal sepsis: sensitivity analysis: risk of bias	21	9105	Risk ratio (IV, Random, 95% CI)	0.78 [0.70, 0.86]
2211. Low or unclear risk of bias for randomization/allocation concealment	19	8892	Risk ratio (IV, Random, 95% CI)	0.79 [0.71, 0.87]
2212. High risk of bias due to randomization/allocation concealment	2	213	Risk ratio (IV, Random, 95% CI)	0.47 [0.25, 0.90]
222. Sepsis specifc mortality	2	4672	Risk ratio (IV, Random, 95% CI)	0.21 [0.04, 1.01]

## SOURCES OF SUPPORT

### Internal sources

- No sources of support provided

### External sources

- Funding, USA

a. Funding for this review came from a grant from the Bill & Melinda Gates Foundation to the Centre for Global Child Health at The Hospital for Sick Children (Grant No. OPP1137750).

**Feedback**

## APPENDIX A

### Literature Search Strategy

Medline Strategy using PubMed

**Vitamin A**

((((("Vitamin A"[Mesh]) OR (Vitamin A[tiab] OR Aquasol A[tiab] OR Retinol[tiab] OR All Trans Retinol[tiab] OR All‐Trans‐Retinol[tiab] OR Vitamin A1[tiab] OR Vitamin A 1[tiab] OR 11‐cis‐Retinol[tiab] OR 11 cis Retinol[tiab] OR Tretinoin[tiab]) AND Supplement*[tiab])) AND (("Infant"[Mesh] OR "Premature Birth"[Mesh]) OR (Neonat*[tiab] OR neo nat*[tiab]) OR (newborn* OR new Born*[tiab] OR newly born*[tiab]) OR (preterm[tiab] OR preterms[tiab] OR pre term[tiab] OR pre terms[tiab]) OR (premature*[tiab] AND (birth*[tiab] OR born[tiab] OR deliver*[tiab])) OR (low[tiab] AND (birthweight*[tiab] OR birth weight*[tiab])) OR (lbw[tiab] OR vlbw[tiab] OR elbw[tiab]) OR infant*[tiab] OR (baby[tiab] OR babies[tiab])))) NOT ("Animals"[Mesh] NOT ("Animals"[Mesh] AND "Humans"[Mesh]))

**Glucose**

(((((("Glucose"[Mesh]) OR (Dextrose OR Glucose[tiab]) AND supplement*)))) AND (("Infant"[Mesh] OR "Premature Birth"[Mesh]) OR (Neonat*[tiab] OR neo nat*[tiab]) OR (newborn* OR new Born*[tiab] OR newly born*[tiab]) OR (preterm[tiab] OR preterms[tiab] OR pre term[tiab] OR pre terms[tiab]) OR (premature*[tiab] AND (birth*[tiab] OR born[tiab] OR deliver*[tiab])) OR (low[tiab] AND (birthweight*[tiab] OR birth weight*[tiab])) OR (lbw[tiab] OR vlbw[tiab] OR elbw[tiab]) OR infant*[tiab] OR (baby[tiab] OR babies[tiab])))) NOT ("Animals"[Mesh] NOT ("Animals"[Mesh] AND "Humans"[Mesh]))

**Probiotics**

((((("Probiotics"[Mesh] OR "Prebiotics"[Mesh] OR "Synbiotics"[Mesh]) OR (Probiotic*[tiab] OR prebiotic*[tiab] OR synbiotic*[tiab]))) AND (("Infant"[Mesh]OR "Premature Birth"[Mesh]) OR (Neonat*[tiab] OR neo nat*[tiab]) OR (newborn* OR new Born*[tiab] OR newly born*[tiab]) OR (preterm[tiab] OR preterms[tiab] OR pre term[tiab] OR pre terms[tiab]) OR (premature*[tiab] AND (birth*[tiab] OR born[tiab] OR deliver*[tiab])) OR (low[tiab] AND (birthweight*[tiab] OR birth weight*[tiab])) OR (lbw[tiab] OR vlbw[tiab] OR elbw[tiab]) OR infant*[tiab] OR (baby[tiab] OR babies[tiab])))) NOT ("Animals"[Mesh] NOT ("Animals"[Mesh] AND "Humans"[Mesh]))

CINAHL Strategies

**Vitamin A**

(MH”Vitamin A") OR TI ("Vitamin A" OR "Aquasol A" OR Retinol OR "All Trans Retinol" OR "All‐Trans‐Retinol" OR "Vitamin A1" OR "Vitamin A 1" OR "11‐cis‐Retinol" OR "11 cis Retinol" OR Tretinoin) OR AB ("Vitamin A" OR "Aquasol A" OR Retinol OR "All Trans Retinol" OR "All‐Trans‐Retinol" OR "Vitamin A1" OR "Vitamin A 1" OR "11‐cis‐Retinol" OR "11 cis retinol" OR Tretinoin)

AND

TI (Supplement*) OR AB (Supplement*) OR MH "Dietary Supplementation" OR MH "Dietary Supplements"

AND

(MH "Infant" OR MH "Infant, Premature" OR MH "Infant, Newborn") OR TI ((Neonat* OR neo nat*) OR (newborn* OR new Born* OR newly born*) OR (preterm OR preterms OR pre term OR pre terms) OR (premature* AND (birth* OR born OR deliver*)) OR (low AND (birthweight* OR birth weight*)) OR (lbw OR vlbw OR elbw) OR infant* OR (baby OR babies)) OR AB ((Neonat* OR neo nat*) OR (newborn* OR new Born* OR newly born*) OR (preterm OR preterms OR pre term OR pre terms) OR (premature* AND (birth* OR born OR deliver*)) OR (low AND (birthweight* OR birth weight*)) OR (lbw OR vlbw OR elbw) OR infant* OR (baby OR babies))

NOT

(MH "Animals" NOT (MH "Animals" AND MH "Humans"))

Limiter: Exclude MEDLINE records

**Glucose**

(MH "Glucose") OR TI (Dextrose OR Glucose) OR AB (Dextrose OR Glucose)

AND

TI (Supplement*) OR AB (Supplement*) OR MH "Dietary Supplementation" OR MH "Dietary Supplements"

AND

(MH "Infant" OR MH "Infant, Premature" OR MH "Infant, Newborn") OR TI ((Neonat* OR neo nat*) OR (newborn* OR new Born* OR newly born*) OR (preterm OR preterms OR pre term OR pre terms) OR (premature* AND (birth* OR born OR deliver*)) OR (low AND (birthweight* OR birth weight*)) OR (lbw OR vlbw OR elbw) OR infant* OR (baby OR babies)) OR AB ((Neonat* OR neo nat*) OR (newborn* OR new Born* OR newly born*) OR (preterm OR preterms OR pre term OR pre terms) OR (premature* AND (birth* OR born OR deliver*)) OR (low AND (birthweight* OR birth weight*)) OR (lbw OR vlbw OR elbw) OR infant* OR (baby OR babies))

NOT

(MH "Animals" NOT (MH "Animals" AND MH "Humans"))

Limiter: Exclude MEDLINE records

**Probiotics**

‘(MH "Probiotics") OR (MH "Prebiotics") OR TI (probiotic* OR prebiotic* OR synbiotic*) OR AB (probiotic* OR prebiotic* OR synbiotic*)

AND

(MH "Infant" OR MH "Infant, Premature" OR MH "Infant, Newborn") OR TI ((Neonat* OR neo nat*) OR (newborn* OR new Born* OR newly born*) OR (preterm OR preterms OR pre term OR pre terms) OR (premature* AND (birth* OR born OR deliver*)) OR (low AND (birthweight* OR birth weight*)) OR (lbw OR vlbw OR elbw) OR infant* OR (baby OR babies)) OR AB ((Neonat* OR neo nat*) OR (newborn* OR new Born* OR newly born*) OR (preterm OR preterms OR pre term OR pre terms) OR (premature* AND (birth* OR born OR deliver*)) OR (low AND (birthweight* OR birth weight*)) OR (lbw OR vlbw OR elbw) OR infant* OR (baby OR babies))

NOT

(MH "Animals" NOT (MH "Animals" AND MH "Humans"))

Limiter: Exclude MEDLINE records

Scopus Strategies

**Vitamin A**

(TITLE‐ABS("Vitamin A" OR "Aquasol A" OR retinol OR "All Trans Retinol" OR "Vitamin A1" OR "11‐cis‐Retinol" OR tretinoin)) AND (TITLE‐ABS(Supplement*)) AND (TITLE‐ABS ((neonat* OR "neo nat*") OR (newborn* OR "new born*" OR "newly born*") OR (preterm OR preterms OR "pre term" OR "pre terms") OR (premature*) AND (birth* OR born OR deliver*) OR (low AND (birthweight* OR "birth weight*")) OR (lbw OR vlbw OR elbow) OR infant* OR (baby OR babies))) AND NOT INDEX(medline)

**Glucose**

TITLE‐ABS (Glucose OR Dextrose) AND TITLE‐ABS (supplement*) AND TITLE‐ABS ((neonat* OR "neo nat*") OR (newborn* OR "new born*" OR "newly born*") OR (preterm OR preterms OR "pre term" OR "pre terms") OR (premature*) AND (birth* OR born OR deliver*) OR (low AND (birthweight* OR "birth weight*")) OR (lbw OR vlbw OR elbow) OR infant* OR (baby OR babies)) AND NOT INDEX (medline)

**Probiotics**

TITLE‐ABS (Probiotic* OR Prebiotic* OR Synbiotic*) AND TITLE‐ABS ((neonat* OR "neo nat*") OR (newborn* OR "new born*" OR "newly born*") OR (preterm OR preterms OR "pre term" OR "pre terms") OR (premature*) AND (birth* OR born OR deliver*) OR (low AND (birthweight* OR "birth weight*")) OR (lbw OR vlbw OR elbow) OR infant* OR (baby OR babies)) AND NOT INDEX (medline)

CENTRAL

**Vitamin A**

1. 1 MeSH descriptor: [infant] explode all trees
2. MeSH descriptor: [Premature Birth] explode all trees
3. (Neonat*:ti,ab OR neo nat*:ti,ab OR (newborn*:ti,ab OR new Born*:ti,ab OR newly born*:ti,ab) OR (preterm:ti,ab OR preterms:ti,ab OR pre term:ti,ab OR pre terms:ti,ab) OR (premature*:ti,ab AND (birth*:ti,ab OR born:ti,ab OR deliver*:ti,ab)) OR (low:ti,ab AND (birthweight*:ti,ab OR birth weight*:ti,ab)) OR (lbw:ti,ab OR vlbw:ti,ab OR elbw:ti,ab) OR infant*:ti,ab OR (baby:ti,ab OR babies:ti,ab)
4. #1 OR #2 OR #3
5. MeSH descriptor: [Animals] explode all trees
6. MeSH descriptor: [Humans] explode all trees
7. (#5 NOT (#5 AND #6))
8. supplement*:ti,ab
9. MeSH descriptor: [Vitamin A] explode all trees
10. "Vitamin A":ti,ab OR "Aquasol A":ti,ab OR Retinol:ti,ab OR "All Trans Retinol":ti,ab OR "All‐Trans‐Retinol":ti,ab OR "Vitamin A1":ti,ab OR "Vitamin A 1":ti,ab OR "11 cis Retinol":ti,ab OR "11‐cis‐Retinol":ti,ab OR Tretinoin:ti,ab
11. #9 OR #10
12. #11 AND #8
13. #12 AND #4 NOT #7
14. "accession number" near pubmed
15. #13 NOT #14

**Glucose**

1. 1 MeSH descriptor: [infant] explode all trees
2. MeSH descriptor: [Premature Birth] explode all trees
3. (Neonat*:ti,ab OR neo nat*:ti,ab) OR (newborn*:ti,ab OR new Born*:ti,ab OR newly born*:ti,ab) OR (preterm:ti,ab OR preterms:ti,ab OR pre term:ti,ab OR pre terms:ti,ab) OR (premature*:ti,ab AND (birth*:ti,ab OR born:ti,ab OR deliver*:ti,ab)) OR (low:ti,ab AND (birthweight*:ti,ab OR birth weight*:ti,ab)) OR (lbw:ti,ab OR vlbw:ti,ab OR elbw:ti,ab) OR infant*:ti,ab OR (baby:ti,ab OR babies:ti,ab)
4. #1 OR #2 OR #3
5. MeSH descriptor: [Animals] explode all trees
6. MeSH descriptor: [Humans] explode all trees
7. (#5 NOT (#5 AND #6))
8. supplement*:ti,ab
9. MeSH descriptor: [Glucose] explode all trees
10. Dextrose:ti,ab OR Glucose:ti,ab
11. #9 OR #10
12. #11 AND #8
13. #12 AND #4 NOT #7
14. "accession number" near pubmed
15. #13 NOT #14

**Probiotics**

1. 1 MeSH descriptor: [infant] explode all trees
2. MeSH descriptor: [Premature Birth] explode all trees
3. (Neonat*:ti,ab OR neo nat*:ti,ab) OR (newborn*:ti,ab OR new Born*:ti,ab OR newly born*:ti,ab) OR (preterm:ti,ab OR preterms:ti,ab OR pre term:ti,ab OR pre terms:ti,ab) OR (premature*:ti,ab AND (birth*:ti,ab OR born:ti,ab OR deliver*:ti,ab)) OR (low:ti,ab AND (birthweight*:ti,ab OR birth weight*:ti,ab)) OR (lbw:ti,ab OR vlbw:ti,ab OR elbw:ti,ab) OR infant*:ti,ab OR (baby:ti,ab OR babies:ti,ab)
4. #1 OR #2 OR #3
5. MeSH descriptor: [Animals] explode all trees
6. MeSH descriptor: [Humans] explode all trees
7. (#5 NOT (#5 AND #6))
8. MeSH descriptor: [Probiotics] explode all trees
9. MeSH descriptor: [Prebiotics] explode all trees
10. MeSH descriptor: [Synbiotics] explode all trees
11. #8 OR #9 OR #10
12. Probiotic*:ti,ab OR prebiotic*:ti,ab OR synbiotic*:ti,ab
13. #11 or #12
14. #13 AND #4 NOT #7
15. "accession number" near pubmed
16. #14 NOT #15

LILACS

**Vitamin A**

(tw:(("Vitamin A"))) OR (ti:(("Aquasol A" OR retinol OR "All Trans Retinol" OR "Vitamin A1" OR "11‐cis‐Retinol" OR tretinoin))) OR (ab:(("Aquasol A" OR retinol OR "All Trans Retinol" OR "Vitamin A1" OR "11‐cis‐Retinol" OR tretinoin))) AND (ti:(supplement*)) OR (ab:(supplement*)) AND (tw:(Infant)) OR (tw:("Premature Birth")) OR (ti:(((neonat* OR "neo nat*") OR (newborn* OR "new born*" OR "newly born*") OR (preterm OR preterms OR "pre term" OR "pre terms") OR (premature*) AND (born OR deliver*) OR (low AND (birthweight* OR "birth weight*")) OR (lbw OR vlbw OR elbw) OR (baby OR babies)))) OR (ab:(((neonat* OR "neo nat*") OR (newborn* OR "new born*" OR "newly born*") OR (preterm OR preterms OR "pre term" OR "pre terms") OR (premature*) AND (born OR deliver*) OR (low AND (birthweight* OR "birth weight*")) OR (lbw OR vlbw OR elbw) OR (baby OR babies)))) AND db:("LILACS")

**Glucose**

((tw:(glucose)) OR (ti:(dextrose)) OR (ab:(dextrose)) AND (ti:(supplement*)) OR (ab:(supplement*))) AND ((tw:(infant)) OR (tw:(“premature birth”)) OR (ti:((neonat* OR "neo nat*") OR (newborn* OR "new born*" OR "newly born*") OR (preterm OR preterms OR "pre term" OR "pre terms") OR (premature*))) AND (ti:((born OR deliver*) OR (low AND (birthweight* OR "birth weight*")) OR (lbw OR vlbw OR elbw) OR (baby OR babies))) OR (ab:((neonat* OR "neo nat*") OR (newborn* OR "new born*" OR "newly born*") OR (preterm OR preterms OR "pre term" OR "pre terms") OR (premature*))) AND (ab:((born OR deliver*) OR (low AND (birthweight* OR "birth weight*")) OR (lbw OR vlbw OR elbw) OR (baby OR babies)))) AND (instance:"regional") AND (db:("LILACS"))

**Probiotics**

((tw:(probiotics OR prebiotics OR synbiotics)) OR (ti:(probiotic* OR prebiotic* OR synbiotic*)) OR (ab:(probiotic* OR prebiotic* OR synbiotic*))) AND ((tw:(infant)) OR (tw:(“premature birth”)) OR (ti:((neonat* OR "neo nat*") OR (newborn* OR "new born*" OR "newly born*") OR (preterm OR preterms OR "pre term" OR "pre terms") OR (premature*))) AND (ti:((born OR deliver*) OR (low AND (birthweight* OR "birth weight*")) OR (lbw OR vlbw OR elbw) OR (baby OR babies))) OR (ab:((neonat* OR "neo nat*") OR (newborn* OR "new born*" OR "newly born*") OR (preterm OR preterms OR "pre term" OR "pre terms") OR (premature*))) AND (ab:((born OR deliver*) OR (low AND (birthweight* OR "birth weight*")) OR (lbw OR vlbw OR elbw) OR (baby OR babies)))) AND (instance:"regional") AND (db:("LILACS"))

EMBASE

**Vitamin A**

1. 1 'retinol'/exp OR 'retinol palmitate'/exp OR '11 cis retinol'/exp OR 'retinoic acid'/exp
2. 'vitamin a':ti,ab OR 'aquasol a':ti,ab OR retinol:ti,ab OR 'all trans retinol':ti,ab OR 'all‐trans‐retinol':ti,ab OR 'vitamin a1':ti,ab OR 'vitamin a 1':ti,ab OR '11‐cis‐retinol':ti,ab OR '11 cis retinol':ti,ab OR tretinoin:ti,ab
3. supplement*:ti,ab
4. 'supplementation'/exp
5. #1 OR #2
6. #3 OR #4
7. #5 AND #6
8. 'infant'/exp OR 'prematurity'/exp OR 'newborn'/exp OR 'low birth weight'/exp OR 'very low birth weight'/exp OR 'extremely low birth weight'/exp OR 'premature labor'/exp
9. neonat*:ti,ab OR 'neo nat*':ti,ab OR newborn*:ti,ab OR 'new born*':ti,ab OR 'newly born*':ti,ab OR preterm:ti,ab OR preterms:ti,ab OR 'pre term':ti,ab OR 'pre terms':ti,ab OR (premature*:ti,ab AND (birth*:ti,ab OR born:ti,ab OR deliver*:ti,ab)) OR (low:ti,ab AND (birthweight*:ti,ab OR 'birth weight*':ti,ab)) OR lbw:ti,ab OR vlbw:ti,ab OR elbw:ti,ab OR infant*:ti,ab OR baby:ti,ab OR babies:ti,ab
10. #8 OR #9
11. #7 AND #10
12. #11 NOT ([animals]/lim NOT [humans]/lim)
13. #12 NOT [medline]/lim
